# Supplementary material for: Phylogeography of 27,000 SARS-CoV-2 Genomes: Europe as the Major Source of the COVID-19 Pandemic
Source: Microorganisms. 2020 Oct 29;8(11):1678. doi: 10.3390/microorganisms8111678 (PMC7693378; doi:10.3390/microorganisms8111678)
Supplement: Supplementary file 1 [file microorganisms-08-01678-s001.zip › microorganisms-972227-supplementary/SupplementaryMaterial.pdf]

Supplementary material

Phylogeography of 27,000 SARS-CoV-2 genomes: Europe as the major source of the COVID-19 pandemic

Teresa Rito[1,2], Martin B. Richards[3], Maria Pala[3], Margarida Correia-Neves[1,2], Pedro Soares[4,5*]

Affiliations

[1] Life and Health Sciences Research Institute (ICVS), School of Medicine, University of Minho, Portugal

[2] ICVS/3B's, PT Government Associate Laboratory, Braga/Guimarães, Portugal

[3] Department of Biological and Geographical Sciences, School of Applied Sciences, University of Huddersfield, UK

[4] Centre of Molecular and Environmental Biology (CBMA), Department of Biology, University of Minho, Portugal

[5] Institute of Science and Innovation for Bio-Sustainability (IB-S), University of Minho, Portugal

Supplementary Tables S2 to S3

Supplementary Figures S1 to S57

Table S2. Major recurrent mutations in the phylogeny of SARS-CoV-2. Occurrences are indicated within a total number of 20,247 mutations.

Mutations	Occurrences
11083T	170
21575	67
241	56
11074	42
3564T	35
3037	35
16887	32
21137	32
24933T	29
9474	28
27384	28
10323	25
19484	25
28253	23
14408	22
23403	22
9438	21
29614	21
28887	20
28851T	20
1912	19
1820	19
28881	19
28854	18
26681	17
29742	17
29095	16
14805	16
8782	16
9491	16
28077T	16
515	16
9430	15
28311	15
24034	15
17766	15
19017	15
10369	15
25916	15
25904	14
26735	14
14786	14
28826	14
583	14
11535T	14
21724T	14
10870T	14
28882	14
28657	13
28849	13
22468T	13
829	13
14724	13
27213	13
15720	13
17410	13
21846	13
21855	12
8790T	12
11572	12
15738	12
6255	12
26447	12
8917	12
683	12
5826	12
29253	12
9634T	12
21742	12
7420	12
1059	12
19524	12
13458	12
635	12
13517	12
3096	12
7765	12
16393	12
16289	12
22624	12
25521	11
26873	11
6402	11
18877	11
9479T	11
15324	11
28708	11
2110	11
29733	11
28093	11
19269	11
27741	11
20178	11
106	11
11962	11
22323	11
16846T	11
6026	11
24378	11
335	11
5869	11
27294	11
28378T	11
29474T	11
29769	11
28373T	11
15237	11
17678	11
27476	11
21304A	11
4158	10
4084	10
29200	10
21648	10
8090	10
28087	10
29700	10
26895	10
2939	10
8078	10
26456	10
29353	10
21305	10
21707	10
5986	10
29543T	10
313	10
7834	10
10582	10
27804	10
27005	10
26111	10
13402G	10
25906T	10
26951T	10
28975T	10
16260	10
11417T	10
3768	9
24023	9
18060	9
24370	9
12880	9
23525	9
1190	9
15960	9
6040	9
25844	9
25511	9
9223	9
29218	9
17747	9
25886	9
14925	9
18568	9
25614	9
7798T	9
20148	9
17304	9
11704	9
5730	9
3784	9
18788	9
1218	9
21058	9
21614	9
7423	9
25047	9
9924	9
9967	9
5183	9
29754	9
884	9
25658	9
9246	9
29296	9
18570	9
16111	9
26801	9
2091	9
5812	9
26062T	9
20762	9
13929	9
24095T	9
25690T	9
26882	9
18255T	9
21622	9
28086T	9
20703	9
19718	9
25688	9
29254T	9
29422T	9
20055	9
15380T	9
24368T	9
28812T	9
5512	9
26461	9
28985T	9
14184	9
25855T	9
1437	9
13536	9
20268	9
18744	9
241C	9
2536	8
3177	8
16466	8
11674	8
11109	8
9857	8
13019	8
26527	8
1594	8
24904	8
25587	8
16575	8
16457	8
16750	8
28957	8
5184	8
26013	8
22432	8
11750	8
12213	8
16329	8
1457	8
17010	8
6781	8
21711	8
6636	8
3619	8
14178	8
27046	8
15277	8
3787	8
3738	8
29625	8
6312A	8
23277	8
680	8
11747	8
29742T	8
29374	8
21789	8
7735	8
4586	8
7011	8
3903	8
25785T	8
21123T	8
1288	8
22225T	8
1149T	8
19086T	8
16375	8
10239A	8
5907	8
22713	8
27703T	8
16616	8
7279	8
9165	8
29585	8
23868T	8
26256	8
11653	8
23593T	8
25218T	8
12525	8
29640	8
7936T	8
29392T	8
25710	8
3602	8
28001T	8
28603	8
5497	8
15857	8
22444	8
8651C	8

Table S3. Potential inter-continental founders. Sink continent and probable origin of clade are indicated as well as the positioning in the phylogeny. Additionally the first detection of the clade in the sink and elsewhere is indicated.

Continent	Clade	Variants	Origin of clade	Frequency in sink dataset	First detection in sink		First detection worldwide	
					Country	Date	Country	Date
Africa	B7	8782, 28144, 28878, 29742	East Asia	2.42	Senegal	20/03/2020	China	23/01/2020
Africa	A1a	241, 3037, 14408, 23403	Europe	6.67	South Africa	07/03/2020	Italy	20/02/2020
Africa	A1a5	241, 3037, 14408, 15324, 23403	Europe	43.64	Senegal	13/03/2020	France/Thailand	03/03/2020
Africa	A1a5+3688	241, 3037, 3688, 14408, 15324, 23403	Europe	1.21	South Africa	31/03/2020	Luxembourg	17/03/2020
Africa	A1a5+8394, 15277	241, 3037, 8394, 14408, 15277, 15324, 23403	Europe	1.21	Democratic Republic of Congo	26/03/2020	England	21/03/2020
Africa	A1a9+8293, 18488, 24739	241, 3037, 8293, 14408, 18488, 23403, 24739, 26530	Europe	9.70	Democratic Republic of Congo	14/03/2020	Democratic Republic of Congo	14/03/2020
Africa	A1a2a1	241, 3037, 4002, 10097, 13536, 14408, 23403, 23731, 28881, 28882	Europe	7.88	Democratic Republic of Congo	21/03/2020	Denmark	02/03/2020
Africa	A1a1c	241, 2416, 3037, 14408, 23403, 25563T	Europe	3.64	Democratic Republic of Congo	09/03/2020	France	29/02/2020
Africa	A1a1c4	241, 2416, 3037, 8371T, 14408, 23403, 25563T	Europe	1.82	Senegal	20/03/2020	Slovakia	06/03/2020

Africa	A1a1a	241, 1059, 3037, 14408, 23403, 25563T	Europe	1.21	Senegal	17/03/2020	France	21/02/2020
Africa	A1a1a5a	241, 1059, 3037, 13006, 14408, 23403, 25563T, 25688	Europe	2.42	Senegal	28/02/2020	Senegal	28/02/2020
Africa	A1a1a+10582	241, 1059, 3037, 10582, 14408, 23403, 25563T	Europe	1.82	Algeria	02/03/2020	France	27/02/2020
Africa	A5a	6312A, 11083T, 13730, 23929, 28311	South Asia	1.21	Gambia	21/03/2020	Taiwan	04/03/2020
Africa	A1a1b2	241, 3037, 7765, 14408, 17690, 18877, 23403, 25563T	Western Asia	1.82	Democratic Republic of Congo	18/03/2020	Israel	18/03/2020
East Asia	A2a1a1	2480, 2558, 11083T, 14805, 26144T	Europe	0.28	Vietnam	17/03/2020	England	09/02/2020
East Asia	A2a1b	11083T, 14805, 17247, 26144T	Europe	0.28	Taiwan	13/03/2020	England	25/02/2020
East Asia	A2a1b1	1515, 9223, 11083T, 14805, 17247, 26144T	Europe	0.28	Thailand	11/03/2020	England	25/02/2020
East Asia	A3	1440, 2891	Europe	0.28	Singapore	17/03/2020	Germany	25/02/2020
East Asia	A1a3c	241, 3037, 14408, 20268, 23403, 29734C	Europe	0.38	Taiwan	17/03/2020	Spain/USA	05/03/2020
East Asia	A1a3a	241, 3037, 14408, 20268, 23403, 28854	Europe	0.19	Thailand	Uknown	USA	05/03/2020
East Asia	A1a3	241, 3037, 14408, 20268, 23403	Europe	0.38	Japan	23/03/2020	Switzerland	27/02/2020

East Asia	A1a	241, 3037, 14408, 23403	Europe	0.10	Vietnam	07/03/2020	Italy	20/02/2020
East Asia	A1a5	241, 3037, 14408, 15324, 23403	Europe	0.47	Thailand	03/03/2020	France/Thailand	03/03/2020
East Asia	A1a9a	241, 3037, 4255T, 14408, 23403, 26530	Europe	0.38	China	14/03/2020	USA	05/03/2020
East Asia	A1a2	241, 3037, 14408, 23403, 28881, 28882	Europe	4.33	Thailand	03/03/2020	England	23/02/2020
East Asia	A1a2b	241, 3037, 14408, 23403, 27046, 28881, 28882	Europe	0.47	Thailand	14/03/2020	Netherlands	24/02/2020
East Asia	A1a2d	241, 313, 3037, 14408, 23403, 28881, 28882	Europe	1.88	Vietnam	13/03/2020	Switzerland	27/02/2020
East Asia	A1a1c	241, 2416, 3037, 14408, 23403, 25563T	Europe	0.28	Vietnam	15/03/2020	France	29/02/2020
East Asia	A1a1a	241, 1059, 3037, 14408, 23403, 25563T	Europe	3.30	China/Vietnam	13/03/2020	France	21/02/2020
East Asia	A1a1a+14786	241, 1059, 3037, 14408, 14786, 23403, 25563T	Europe	0.38	Thailand	18/03/2020	Iceland	09/03/2020
East Asia	B1a1a	8782, 17747, 17858, 18060, 24694T, 28144	North America	0.38	Taiwan	19/03/2020	Canada/Australia	05/03/2020
East Asia	A1a1b	241, 3037, 14408, 18877, 23403, 25563T	North America	1.13	Taiwan	02/03/2020	USA/Canada	29/02/2020
East Asia	A1a1a+ 1380	241, 1059, 1380, 3037, 14408, 23403, 25563T	North America	0.19	Thailand	28/03/2020	USA	14/03/2020

East Asia	A1a2+ 11638, 29708	241, 3037, 11638, 14408, 23403, 28881, 28882, 29708	Oceania	0.19	Singapore	18/03/2020	Australia	10/03/2020
East Asia	A1a2+25088T	241, 3037, 14408, 23403, 25088T, 28881, 28882	South America	0.19	China	15/03/2020	Brazil	05/03/2020
East Asia	A5a	6312A, 11083T, 13730, 23929, 28311	South Asia	6.97	Taiwan	04/03/2020	Taiwan	04/03/2020
East Asia	A5a1	6310A, 6312A, 11083T, 13730, 19524, 23929, 28311	South Asia	5.08	Singapore	16/03/2020	Australia	13/03/2020
East Asia	A5b+9514	1397, 9514, 11083T, 28688, 29742T	Western Asia	0.47	China	13/03/2020	United Arab Emirates	25/02/2020
Europe	B7	8782, 28144, 28878, 29742	East Asia	0.03	Netherlands	08/03/2020	China	23/01/2020
Europe	B	8782, 28144	East Asia	0.69	England	29/01/2020	China	05/01/2020
Europe	Pre-B2	8782, 9477A, 28144	East Asia	1.24	France	25/02/2020	China	01/02/2020
Europe	A	0	East Asia	10.45	Finland/France	29/01/2020	China	24/12/2019
Europe	A2	26144T	East Asia	0.05	France	23/01/2020	China	21/01/2020
Europe	A2a	11083T, , 26144T	East Asia	0.21	Italy	29/01/2020	China	23/01/2020
Europe	A2a1	11083T, 14805, 26144T	East Asia	13.00	England	25/02/2020	Korea	18/02/2020
Europe	A5+1190	1190, 11083T	East Asia	0.01	France	29/01/2020	France	29/01/2020

Europe	A5b	1397, 11083T, 28688, 29742T	East Asia	0.03	Sweden	27/02/2020	China	18/01/2020
Europe	A7	21707	East Asia	0.01	Poland	29/03/2020	China	17/01/2020
Europe	A1	241, 3037, 23403	East Asia	60.52	Germany	28/01/2020	China/Germany	28/01/2020
Europe	B1a1	8782, 17747, 17858, 18060, 28144	North America	0.04	England	13/03/2020	USA	20/02/2020
Europe	B1a	8782, 17747, 17858, 18060, 24694T, 28144	North America	0.10	Iceland	11/03/2020	Canada/Australia	05/03/2020
Europe	B3a2	490A, 3177, 8782, 18736, 19684T, 24034, 26729, 27635, 28077C, 28144, 29700	North America	0.07	England	15/03/2020	USA	05/03/2020
Europe	B7+22468T	8782, 22468T, 28144, 28878, 29742	North America	0.01	Russia	23/03/2020	USA	06/03/2020
Europe	A2a1a1+ 21575	2480, 2558, 11083T, 14805, 21575, 26144T	North America	0.05	England	18/03/2020	USA	04/03/2020
Europe	A6	29711T	North America	0.07	Iceland	16/03/2020	USA	28/02/2020
Europe	A1a3+17639, 28638	241, 3037, 14408, 17639, 20268, 23403, 28638	North America	0.08	Spain	12/03/2020	Mexico	11/03/2020
Europe	A1c1c3	241, 2416, 3037, 14408, 23403, 25563T, 26233T	North America	0.01	Slovakia	07/03/2020	USA	05/03/2020
Europe	A1a1b	241, 3037, 14408, 18877, 23403, 25563T	North America	0.16	Greece	05/03/2020	USA/Canada	29/02/2020

Europe	A1a1b + 8290	241, 3037, 8290, 14408, 18877, 23403, 25563T	North America	0.01	Romania	30/04/2020	USA	02/04/2020
Europe	A1a1b + 1918	241, 1918, 3037, 14408, 18877, 23403, 25563T	North America	0.03	Wales	04/04/2020	USA	24/03/2020
Europe	A1a1a+3253	241, 1059, 3037, 3253, 14408, 23403, 25563T	North America	0.01	England	28/03/2020	USA	12/03/2020
Europe	A1a1a+ 29614	241, 1059, 3037, 14408, 23403, 25563T, 29614	North America	0.01	France	02/04/2020	USA	19/03/2020
Europe	A1a1a3	241, 1059, 3037, 11916, 14408, 23403, 25563T	North America	0.09	England	30/03/2020	USA	05/03/2020
Europe	A1a1a+27384	241, 1059, 3037, 14408, 23403, 25563T, 27384	North America	0.01	Luxembourg	14/04/2020	USA	05/04/2020
Europe	A1a1a+27294	241, 1059, 3037, 14408, 23403, 25563T, 27294	North America	0.01	Sweden	11/03/2020	Sweden	11/03/2020
Europe	A1a1a+ 21976	241, 1059, 3037, 14408, 21976, 23403, 25563T	North America	0.03	Denmark	27/03/2020	USA	16/03/2020
Europe	A1a1a+ 10449	241, 1059, 3037, 10449, 14408, 23403, 25563T	North America	0.01	Russia	23/03/2020	USA	18/03/2020
Europe	A1a1a8	241, 1059, 3037, 14408, 20755C, 23403, 25563T	North America	0.04	England	23/03/2020	Australia	09/03/2020
Europe	A1a1a+ 25217T	241, 1059, 3037, 14408, 23403, 25217T, 25563T	North America	0.01	England	30/03/2020	Canada	12/03/2020

Europe	A2a1a1+ 9170	2480, 2558, 9170, 11083T, 14805, 26144T	Oceania	0.01	England	19/03/2020	Australia	07/03/2020
Europe	A1a2+ 11638, 29708	241, 3037, 11638, 14408, 23403, 28881, 28882, 29708	Oceania	0.08	Netherlands	16/03/2020	Australia	10/03/2020
Europe	A1a1b+10507 + 476	241, 476, 3037, 10507, 14408, 18877, 23403, 25563T	South America	0.03	England	19/04/2020	Colombia	31/03/2020
Europe	A5a	6312A, 11083T, 13730, 23929, 28311	South Asia	0.03	England	24/03/2020	Taiwan	04/03/2020
Europe	A5b+884, 8653T	884, 1397, 8653T, 11083T, 28688, 29742T	Western Asia	0.04	Norway	26/02/2020	Norway	26/02/2020
Europe	A5b+9514	1397, 9514, 11083T, 28688, 29742T	Western Asia	0.01	Netherlands	06/03/2020	United Arab Emirates	25/02/2020
Europe	A1a1b2a	241, 2113, 3037, 7765, 14408, 17690, 18877, 23403, 25563T	Western Asia	0.20	Belgium	14/03/2020	Belgium	14/03/2020
Europe	A1a1b1	241, 3037, 14408, 18877, 23403, 25563T, 26735	Western Asia	0.31	Denmark	11/03/2020	Saudi Arabia	10/03/2020
North America	B	8782, 28144	East Asia	0.03	USA	22/01/2020	China	05/01/2020
North America	B+18060	8782, 28144, 18060	East Asia	0.10	USA	19/01/2020	USA	19/01/2020
North America	B+29095	8782, 28144, 29095	East Asia	0.03	USA	22/01/2020	China	10/01/2020

North America	B1	8782, 28144, 18060	East Asia	18.89	USA	19/01/2020	USA	19/01/2020
North America	B3	490A, 8782, 24034, 26729, 28077C, 28144	East Asia	2.65	USA	21/01/2020	China	15/01/2020
North America	B7	8782, 28144, 28878, 29742	East Asia	0.27	USA	06/02/2020	China	23/01/2020
North America	A	0	East Asia	5.04	Canada	23/01/2020	China	24/12/2019
North America	A2a1+5572T	5572T, 11083T, 14805, 26144T	East Asia	0.03	USA	27/02/2020	Korea	18/02/2020
North America	A2a1	11083T, 14805, 26144T,	East Asia	0.17	USA	27/02/2020	Korea	18/02/2020
North America	A5	11083T	East Asia	0.17	USA	17/02/2020	China	18/01/2020
North America	A5b	1397, 11083T, 28688, 29742T	East Asia	0.27	Canada	16/02/2020	China	18/01/2020
North America	A1	241, 3037, 23403	East Asia	0.03	USA	12/03/2020	China/Germany	28/01/2020
North America	B4	8782, 26088, 28144	Europe	0.05	Mexico	10/03/2020	Australia	23/02/2020
North America	B4a	8782, 17470, 26088, 28144	Europe	0.03	Mexico	04/03/2020	Chile	02/03/2020
North America	B2	8782, 9477A, 14805, 23280, 25979T, 28144, 28657, 28863	Europe	0.08	USA	04/03/2020	France	25/02/2020
North America	B7+ 16381	8782, 16381, 28144, 28878, 29742	Europe	0.40	USA	17/03/2020	Netherlands	08/03/2020
North America	A2a1c	11083T, 14805, 26144T, 28842T	Europe	0.03	USA	18/03/2020	Denmark	09/03/2020

North America	A2a1c+18086	11083T, 14805, 18086, 26144T, 28842T	Europe	0.07	USA	17/03/2020	Denmark	09/03/2020
North America	A2a1a	2558, 11083T, 14805, 26144T	Europe	0.27	USA	10/03/2020	England	28/02/2020
North America	A2a1a1	2480, 2558, 11083T, 14805, 26144T	Europe	0.32	USA	04/03/2020	England	09/02/2020
North America	A2a1b	11083T, 14805, 17247, 26144T	Europe	1.06	USA	05/03/2020	England	25/02/2020
North America	A2a1b1	1515, 9223, 11083T, 14805, 17247, 26144T	Europe	0.12	USA	11/03/2020	England	25/02/2020
North America	A3	1440, 2891	Europe	0.07	USA	05/03/2020	Germany	25/02/2020
North America	A3b	1440, 2891, 7011	Europe	0.05	USA	10/03/2020	Netherlands, Denmark, Austria	09/03/2020
North America	A4	1605delATG	Europe	0.03	USA	16/03/2020	England	05/02/2020
North America	A4a1	514, 1605delATG, 17410	Europe	0.07	USA	07/03/2020	Netherlands	27/02/2020
North America	A1a3c	241, 3037, 14408, 20268, 23403, 29734C	Europe	0.10	USA	05/03/2020	Spain/USA	05/03/2020
North America	A1a3a	241, 3037, 14408, 20268, 23403, 28854	Europe	0.24	USA	05/03/2020	USA	05/03/2020
North America	A1a3	241, 3037, 14408, 20268, 23403	Europe	0.49	USA	28/02/2020	Switzerland	27/02/2020
North America	A1a4b	241, 3037, 13730, 14408, 23403, 25429T	Europe	0.10	USA	02/03/2020	USA	02/03/2020
North America	A1a	241, 3037, 14408, 23403	Europe	4.58	USA	07/03/2020	Italy	20/02/2020

North America	A1a5	241, 3037, 14408, 15324, 23403	Europe	0.13	USA	22/03/2020	France/Thailand	03/03/2020
North America	A1a5+21137	241, 3037, 14408, 15324, 21137, 23403	Europe	0.12	USA	13/04/2020	France	21/03/2020
North America	A1a5a	241, 3037, 14408, 15324, 23403, 25433	Europe	0.56	Canada	09/03/2020	France	03/03/2020
North America	A1a6	241, 3037, 14408, 23403, 25350	Europe	0.10	USA	12/03/2020	Australia	22/02/2020
North America	A1a8	187, 241, 3037, 14408, 23403	Europe	0.24	Canada	24/02/2020	Switzerland	24/02/2020
North America	A1a9+ 8293, 18488, 24739	241, 3037, 8293, 14408, 18488, 23403, 24739, 26530	Europe	0.03	Canada	27/03/2020	Democratic Republic of Congo	14/03/2020
North America	A1a9a	241, 3037, 4255T, 14408, 23403, 26530	Europe	0.07	USA	05/03/2020	USA	05/03/2020
North America	A1a24	241, 3037, 14408, 23403, 23575, 23587C	Europe	0.08	USA	14/03/2020	Luxembourg	29/02/2020
North America	A1a31	241, 3037, 11083T, 14408, 23403, 25575C	Europe	0.05	USA	18/03/2020	Netherlands	01/03/2020
North America	A1a2+21575	241, 3037, 14408, 21575, 23403, 28881, 28882	Europe	0.03	USA	01/04/2020	Portugal	18/03/2020
North America	A1a2 + 10948	241, 3037, 10948, 14408, 23403, 28881, 28882	Europe	0.15	USA	14/03/2020	England	11/03/2020
North America	A1a2	241, 3037, 14408, 23403, 28881, 28882	Europe	2.01	USA	29/02/2020	England	23/02/2020

North America	A1a1	241, 3037, 14408, 23403, 25563T	Europe	4.38	USA	02/03/2020	France	26/02/2020
North America	A1a2b	241, 3037, 14408, 23403, 27046, 28881, 28882	Europe	0.07	USA	13/03/2020	Netherlands	24/02/2020
North America	A1a2b + 16075T 18689A 20031A	241, 3037, 14408, 16075T, 18689A, 20031A, 23403, 27046, 28881, 28882	Europe	0.03	USA	13/03/2020	Scotland	11/03/2020
North America	A1a2d	241, 313, 3037, 14408, 23403, 28881, 28882	Europe	0.47	USA	28/02/2020	Switzerland	27/02/2020
North America	A1a2d +18826T	241, 313, 3037, 14408, 18826T, 23403, 28881, 28882	Europe	0.03	USA	17/03/2020	Chile	10/03/2020
North America	A1a2b+29614	241, 313, 3037, 14408, 23403, 28881, 28882, 29164	Europe	0.07	USA	17/03/2020	USA	17/03/2020
North America	A1a2a1	241, 3037, 4002, 10097, 13536, 14408, 23403, 23731, 28881, 28882	Europe	0.07	USA	10/03/2020	Denmark	02/03/2020
North America	A1a2c	241, 3037, 14408, 19839, 23403, 28881, 28882	Europe	0.08	USA	02/03/2020	Switzerland	26/02/2020
North America	A1a2h + 28077T	241, 3037, 14408, 19170, 19509, 23403, 28077T, 28881, 28882	Europe	0.03	Canada	14/03/2020	Canada	14/03/2020

North America	A1a2+ 25521	241, 3037, 14408, 23403, 25521, 28881, 28882	Europe	0.03	USA	13/04/2020	England	02/04/2020
North America	A1a2g	241, 3037, 14408, 14786, 23403, 28881, 28882	Europe	0.08	USA	14/03/2020	USA	14/03/2020
North America	A1a2ak	241, 3037, 10265, 14408, 23403, 28881, 28882	Europe	0.17	USA	11/03/2020	Germany	25/02/2020
North America	A1a2e	241, 3037, 3373A, 14408, 23403, 28881, 28882	Europe	0.07	Canada	10/03/2020	Switzerland	27/02/2020
North America	A1a1c	241, 2416, 3037, 14408, 23403, 25563T	Europe	2.04	USA	04/03/2020	France	29/02/2020
North America	A1a1a	241, 1059, 3037, 14408, 23403, 25563T	Europe	49.15	USA	02/03/2020	France	21/02/2020
North America	A1a1a+25585	241, 1059, 3037, 14408, 23403, 25563T, 25585	Europe	0.19	USA	01/04/2020	Germany	11/03/2020
North America	A1a1a+14786	241, 1059, 3037, 14408, 14786, 23403, 25563T	Europe	0.03	USA	16/03/2020	Iceland	09/03/2020
North America	A1a1a5a	241, 1059, 3037, 13006, 14408, 23403, 25563T, 25688	Europe	0.07	USA	29/02/2020	Senegal	28/02/2020
North America	A1a1a+21697A	241, 1059, 3037, 14408, 21697A, 23403, 25563T	Europe	0.03	USA	05/04/2020	Germany	16/03/2020
North America	A1a1a+10582	241, 1059, 3037, 10582, 14408, 23403, 25563T	Europe	0.22	USA	27/03/2020	France	27/02/2020

North America	A1a1a14	241, 1059, 11083T, 14408, 23403, 25563T	Europe	0.42	USA	13/03/2020	Czech Republic	08/03/2020
North America	A5b+29742T!	1397, 11083T, 28688	Oceania	0.03	Canada	04/03/2020	Australia	03/03/2020
North America	A1a2w1	241, 3037, 14408, 23403, 27299, 28881, 28882, 29148	South America	0.13	Canada	07/03/2020	Canada	07/03/2020
North America	A5a	6312A, 11083T, 13730, 23929, 28311	South Asia	0.19	USA	11/03/2020	Taiwan	04/03/2020
North America	A5b+884, 8653T	884, 1397, 8653T, 11083T, 28688, 29742T	Western Asia	0.17	Canada	02/03/2020	Norway	26/02/2020
North America	A5b+9514	1397, 9514, 11083T, 28688, 29742T	Western Asia	0.05	Canada	11/03/2020	United Arab Emirates	25/02/2020
Oceania	B7	8782, 28144, 28878, 29742	East Asia	0.50	Australia	28/01/2020	China	23/01/2020
Oceania	B	8782, 28144	East Asia	0.25	Australia	24/01/2020	China	05/01/2020
Oceania	A	0	East Asia	0.99	Australia	22/01/2020	China	24/12/2019
Oceania	A2	26144T	East Asia	0.33	Australia	24/01/2020	China	21/01/2020
Oceania	A5	11083T	East Asia	0.25	Australia	21/02/2020	China	18/01/2020
Oceania	A5b	1397, 11083T, 28688, 29742T	East Asia	5.96	New Zealand	05/03/2020	China	18/01/2020
Oceania	B2	8782, 9477A, 14805, 23280, 25979T, 28144, 28657, 28863	Europe	0.25	Australia	20/03/2020	France	25/02/2020

Oceania	B2a1	4540, 8782, 9477A, 14805, 25979T, 28144, 28311, 28657, 28863	Europe	5.46	Australia	16/03/2020	England	26/03/2020
Oceania	B4	8782, 26088, 28144	Europe	0.33	Australia	23/02/2020	Australia	23/02/2020
Oceania	A2a1d+1368, 6990	1368, 6990, 11083T, 14805, 23707, 26144T	Europe	0.66	Australia	13/03/2020	Australia	13/03/2020
Oceania	A2a1d	11083T, 14805, 23707, 26144T	Europe	0.17	Australia	23/03/2020	England	27/02/2020
Oceania	A2a1c	11083T, 14805, 26144T, 28842T	Europe	0.17	Australia	18/03/2020	Denmark	09/03/2020
Oceania	A2a1e	11083T, 13627T, 14805, 15540, 26144T, 28338	Europe	1.82	New Zealand	11/03/2020	England/New Zealand	11/03/2020
Oceania	A2a1a1	2480, 2558, 11083T, 14805, 26144T	Europe	2.56	Australia	07/03/2020	England	09/02/2020
Oceania	A2a1b	11083T, 14805, 17247, 26144T	Europe	1.41	Australia	08/03/2020	England	25/02/2020
Oceania	A2a1b1	1515, 9223, 11083T, 14805, 17247, 26144T	Europe	0.91	Australia	08/03/2020	England	25/02/2020
Oceania	A2a1b3	7479, 11083T, 14805, 17247, 25572, 26144T, 28887	Europe	0.17	Australia	20/03/2020	Scotland	17/03/2020
Oceania	A3	1440, 2891	Europe	0.25	Australia	23/03/2020	Germany	25/02/2020
Oceania	A3b	1440, 2891, 7011	Europe	0.58	Australia	10/03/2020	Netherlands, Denmark, Austria	09/03/2020
Oceania	A3c	1440, 2891, 27669	Europe	0.25	Australia	22/03/2020	England	12/03/2020

Oceania	A4	1605delATG	Europe	0.41	Australia	16/03/2020	England	05/02/2020
Oceania	A4+1904, 8090, 10789	1605delATG, 1904, 8090, 10789	Europe	0.58	Australia	27/03/2020	England	12/03/2020
Oceania	A1a3f	241, 2937, 3037, 14408, 20268, 23403	Europe	0.17	Australia	01/04/2020	England	21/03/2020
Oceania	A1a3+ 25483	241, 3037, 14408, 20268, 23403, 25483	Europe	0.33	Australia	13/03/2020	Australia	13/03/2020
Oceania	A1a3c	241, 3037, 14408, 20268, 23403, 29734C	Europe	0.33	Australia	15/03/2020	Spain/USA	05/03/2020
Oceania	A1a3a	241, 3037, 14408, 20268, 23403, 28854	Europe	0.66	Australia	15/03/2020	USA	05/03/2020
Oceania	A1a3	241, 3037, 14408, 20268, 23403	Europe	1.90	Australia	17/03/2020	Switzerland	27/02/2020
Oceania	A1a4	241, 3037, 14408, 23403, 25429T	Europe	0.33	Australia	20/03/2020	USA	02/03/2020
Oceania	A1a4a	241, 3037, 14408, 16289, 23403, 25429T	Europe	0.17	Australia	20/03/2020	England	12/03/2020
Oceania	A1a	241, 3037, 14408, 23403	Europe	7.28	Australia	10/03/2020	Italy	20/02/2020
Oceania	A1a5	241, 3037, 14408, 15324, 23403	Europe	1.16	Australia	18/03/2020	France/Thailand	03/03/2020
Oceania	A1a5+28690T	241, 3037, 14408, 15324, 23403, 28690T	Europe	0.33	Australia	23/03/2020	Luxembourg	17/03/2020
Oceania	A1a6	241, 3037, 14408, 23403, 25350	Europe	0.74	Australia	22/02/2020	Australia	22/02/2020
Oceania	A1a8	187, 241, 3037, 14408, 23403	Europe	0.25	Australia	22/03/2020	Switzerland	24/02/2020

Oceania	A1a9	241, 3037, 14408, 23403, 26530	Europe	1.08	Australia	11/03/2020	England	26/02/2020
Oceania	A1a11	241, 3037, 14408, 23403, 29353	Europe	5.96	Australia	18/03/2020	Australia	18/03/2020
Oceania	A1a20	241, 3037, 14408, 23403, 24862	Europe	0.25	Australia	24/03/2020	Netherlands	Netherlands
Oceania	A1a2	241, 3037, 14408, 23403, 28881, 28882	Europe	7.78	New Zealand	02/03/2020	England	23/02/2020
Oceania	A1a1+1594, 11109	241, 1594, 3037, 11109, 14408, 23403, 25563T	Europe	0.74	Australia	31/03/2020	Netherlands	16/03/2020
Oceania	A1a2b	241, 3037, 14408, 23403, 27046, 28881, 28882	Europe	0.66	Australia	21/03/2020	Netherlands	24/02/2020
Oceania	A1a2b+1514	241, 1514, 3037, 14408, 23403, 27046, 28881, 28882	Europe	0.17	Australia	28/03/2020	England	04/03/2020
Oceania	A1a2d	241, 313, 3037, 14408, 23403, 28881, 28882	Europe	0.50	Australia	16/03/2020	Switzerland	27/02/2020
Oceania	A1a2a1b	241, 3037, 4002, 10097, 13536, 14184, 14408, 21058, 23403, 23731, 28881, 28882	Europe	0.17	Australia	21/03/2020	Vietnam	15/03/2020
Oceania	A1a2a1	241, 3037, 4002, 10097, 13536, 14408, 23403, 23731, 28881, 28882	Europe	0.25	Australia	21/03/2020	Denmark	02/03/2020

Oceania	A1a2c	241, 3037, 14408, 19839, 23403, 28881, 28882	Europe	0.33	Australia	08/03/2020	Switzerland	26/02/2020
Oceania	A1a2h + 28077T	241, 3037, 14408, 19170, 19509, 23403, 28077T, 28881, 28882	Europe	0.17	Australia	27/03/2020	Canada	14/03/2020
Oceania	A1a2+8102T	241, 3037, 8102T, 14408, 23403, 28881, 28882	Europe	0.58	Australia	30/03/2020	Australia	30/03/2020
Oceania	A1a2g	241, 3037, 14408, 14786, 23403, 28881, 28882	Europe	0.17	Australia	20/03/2020	USA	14/03/2020
Oceania	A1a2ak	241, 3037, 10265, 14408, 23403, 28881, 28882	Europe	0.17	Australia	19/03/2020	Germany	25/02/2020
Oceania	A1a2e	241, 3037, 3373A, 14408, 23403, 28881, 28882	Europe	0.50	Australia	18/03/2020	Switzerland	27/02/2020
Oceania	A1a2e1	241, 3037, 3373A, 11195, 14408, 23403, 28881, 28882	Europe	0.17	Australia	23/03/2020	England	12/03/2020
Oceania	A1a2r	241, 3037, 14408, 23403, 27281T, 28881, 28882	Europe	0.17	Australia	21/03/2020	England	18/03/2020
Oceania	A1a2x	241, 3037, 6573, 14408, 23403, 25528, 28881, 28882	Europe	0.41	Australia	27/03/2020	England	18/03/2020
Oceania	A1a2v	241, 3037, 14408, 21724T, 23403, 28881, 28882,	Europe	0.25	Australia	22/03/2020	England	17/03/2020

Oceania	A1a1c	241, 2416, 3037, 14408, 23403, 25563T	Europe	0.17	Australia	17/03/2020	France	29/02/2020
Oceania	A1a1c1	241, 2416, 3037, 14408, 20578T, 23403, 25563T	Europe	0.66	Australia	19/03/2020	England	09/03/2020
Oceania	A1a1c1a	241, 2416, 3037, 14408, 14786, 20578T, 23403, 25563T	Europe	0.25	Australia	16/03/2020	Australia	16/03/2020
Oceania	A1a1c4	241, 2416, 3037, 8371T, 14408, 23403, 25563T	Europe	0.25	Australia	20/03/2020	Slovakia	06/03/2020
Oceania	A1a1a	241, 1059, 3037, 14408, 23403, 25563T	Europe	9.93	Australia	10/03/2020	France	21/02/2020
Oceania	A1a1a5a	241, 1059, 3037, 13006, 14408, 23403, 25563T, 25688	Europe	0.17	Australia	19/03/2020	Senegal	28/02/2020
Oceania	A1a1a7	241, 1059, 3037, 14408, 15380T, 23403, 25563T	Europe	0.17	Australia	20/03/2020	Denmark	08/03/2020
Oceania	A1a1a14	241, 1059, 11083T, 14408, 23403, 25563T	Europe	0.17	Australia	27/03/2020	Czech Republic	08/03/2020
Oceania	B1a1	8782, 17747, 17858, 18060, 28144	North America	2.23	Australia	11/03/2020	USA	20/02/2020
Oceania	B1a1+10771	8782, 10771, 17747, 17858, 18060, 28144	North America	0.17	Australia	11/03/2020	Canada	07/03/2020

Oceania	B1a1a	8782, 17747, 17858, 18060, 24694T, 28144	North America	4.71	Australia	05/03/2020	Canada/Australia	05/03/2020
Oceania	B3a2	490A, 3177, 8782, 18736, 19684T, 24034, 26729, 27635, 28077C, 28144, 29700	North America	2.56	Australia	07/03/2020	USA	05/03/2020
Oceania	B3a2a	490A, 3177, 6285, 8782, 15960, 18736, 19684T, 24034, 27635, 28077C, 28144, 29700	North America	0.91	Australia	24/03/2020	USA	17/03/2020
Oceania	B7 + 2676, 22606T	2676, 8782, 22606T, 28144, 28878, 29742	North America	0.17	Australia	17/03/2020	USA	12/03/2020
Oceania	A1a1+8389	241, 3037, 8389, 14408, 23403, 25563T	North America	0.25	Australia	26/03/2020	USA	02/03/2020
Oceania	A1c1c3 + 12112, 21974C	241, 2416, 3037, 12112, 14408, 21974C, 23403, 25563T, 26233T	North America	0.50	Australia	27/03/2020	USA	22/03/2020
Oceania	A1a1b	241, 3037, 14408, 18877, 23403, 25563T	North America	0.74	Australia	17/03/2020	USA/Canada	29/02/2020
Oceania	A1a1a2	241, 1059, 3037, 14408, 23403, 25563T, 27964	North America	1.08	Australia	24/03/2020	USA	08/03/2020
Oceania	A1a1a3	241, 1059, 3037, 11916, 14408, 23403, 25563T	North America	0.25	Australia	20/03/2020	USA	05/03/2020

Oceania	A1a1a+ 21648	241, 1059, 3037, 14408, 21648, 23403, 25563T	North America	0.58	Australia	21/03/2020	USA	09/03/2020
Oceania	A1a1a8	241, 1059, 3037, 14408, 20755C, 23403, 25563T	North America	0.58	Australia	09/03/2020	Australia	09/03/2020
Oceania	A1a1a+ 26625	241, 1059, 3037, 14408, 23403, 25563T, 26625	North America	0.41	Australia	24/03/2020	USA	15/03/2020
Oceania	A1a1a11	241, 379A, 1059, 3037, 14408, 23403, 25563T	North America	0.41	Australia	17/03/2020	USA	07/03/2020
Oceania	A1a1a12	241, 1059, 10851, 14408, 23403, 25563T	North America	2.81	Australia	21/03/2020	USA	09/03/2020
Oceania	A1a1+8389 +1319	241, 1319, 3037, 8389, 14408, 23403, 25563T	South America	0.25	Australia	26/03/2020	Chile	12/03/2020
Oceania	A1a2w1	241, 3037, 14408, 23403, 27299, 28881, 28882, 29148	South America	0.17	Australia	27/03/2020	Canada	07/03/2020
Oceania	A5a	6312A, 11083T, 13730, 23929, 28311	South Asia	3.47	Australia	05/03/2020	Taiwan	04/03/2020
Oceania	A5a1+12685T	6310A, 6312A, 11083T, 12685T, 13730, 19524, 23929, 28311	South Asia	0.17	Australia	23/03/2020	Australia	23/03/2020
Oceania	A5a1	6310A, 6312A, 11083T, 13730, 19524, 23929, 28311	South Asia	0.41	Australia	13/03/2020	Australia	13/03/2020

Oceania	A5b+884, 8653T	884, 1397, 8653T, 11083T, 28688, 29742T	Western Asia	0.83	Australia	02/03/2020	Norway	26/02/2020
Oceania	A5b+9514	1397, 9514, 11083T, 28688, 29742T	Western Asia	0.33	Australia	30/03/2020	United Arab Emirates	25/02/2020
South America	A	0	East Asia	0.72	Colombia	31/03/2020	China	24/12/2019
South America	B2	8782, 9477A, 14805, 23280, 25979T, 28144, 28657, 28863	Europe	4.32	Chile	03/03/2020	France	25/02/2020
South America	B4	8782, 26088, 28144	Europe	0.72	Chile	10/03/2020	Australia	23/02/2020
South America	B4+19983	8782, 19983, 26088, 28144	Europe	0.72	Chile	12/03/2020	Chile	12/03/2020
South America	B4a	8782, 17470, 126088, 28144	Europe	4.32	Chile	02/03/2020	Chile	02/03/2020
South America	A2a1b	11083T, 14805, 17247, 26144T	Europe	1.80	Brazil	28/02/2020	England	25/02/2020
South America	A4	1605delATG	Europe	0.72	Chile	15/03/2020	England	05/02/2020
South America	A1a3 + 5230T	241, 3037, 5230T, 14408, 20268, 23403	Europe	0.72	Chile	16/03/2020	Chile	16/03/2020
South America	A1a3c	241, 3037, 14408, 20268, 23403, 29734C	Europe	1.44	Chile	09/03/2020	Spain/USA	05/03/2020
South America	A1a3	241, 3037, 14408, 20268, 23403	Europe	14.03	Chile	09/03/2020	Switzerland	27/02/2020
South America	A1a	241, 3037, 14408, 23403	Europe	4.68	Brazil	04/03/2020	Italy	20/02/2020
South America	A1a5	241, 3037, 14408, 15324, 23403	Europe	0.72	Chile	17/03/2020	France/Thailand	03/03/2020

South America	A1a6	241, 3037, 14408, 23403, 25350	Europe	2.52	Chile	14/03/2020	Australia	22/02/2020
South America	A1a15	241, 3037, 14408, 23403, 29144	Europe	0.72	Chile	11/03/2020	Portugal	01/03/2020
South America	A1a2	241, 3037, 14408, 23403, 28881, 28882	Europe	8.99	Brazil/Chile	05/03/2020	England	23/02/2020
South America	A1a2w	241, 3037, 14408, 23403, 28881, 28882, 29148	Europe	8.27	Brazil	13/03/2020	Switzerland/Netherlands	28/02/2020
South America	A1a1	241, 3037, 14408, 23403, 25563T	Europe	0.72	Brazil	13/03/2020	France	26/02/2020
South America	A1a2b	241, 3037, 14408, 23403, 27046, 28881, 28882	Europe	1.08	Brazil	02/03/2020	Netherlands	24/02/2020
South America	A1a2d	241, 313, 3037, 14408, 23403, 28881, 28882	Europe	2.52	Brasil	04/03/2020	Switzerland	27/02/2020
South America	A1a2c	241, 3037, 14408, 19839, 23403, 28881, 28882	Europe	0.72	Colombia	06/03/2020	Switzerland	26/02/2020
South America	A1a2h	241, 3037, 14408, 19170, 19509, 23403, 28881, 28882	Europe	0.72	Chile	10/03/2020	England	06/03/2020
South America	A1a2+7728	241, 3037, 7728, 14408, 23403, 28881, 28882	Europe	0.72	Colombia	04/04/2020	Luxembourg	22/03/2020
South America	A1a2ak	241, 3037, 10265, 14408, 23403, 28881, 28882	Europe	4.32	Brazil	04/03/2020	Germany	25/02/2020
South America	A1a1a	241, 1059, 3037, 14408, 23403, 25563T	Europe	11.51	Brazil	10/03/2020	France	21/02/2020

South America	A1a2a1	241, 3037, 4002, 10097, 13536, 14408, 23403, 23731, 28881, 28882	Europe	3.24	Chile	01/04/2020	Denmark	02/03/2020
South America	B1a1a	8782, 17747, 17858, 18060, 24694T, 28144	North America	0.72	Uruguay	17/03/2020	Canada/Australia	05/03/2020
South America	A1a1b	241, 3037, 14408, 18877, 23403, 25563T	North America	9.71	Brazil	09/03/2020	USA/Canada	29/02/2020
South America	A1a1a3+19018	241, 1059, 3037, 11916, 14408, 19018, 23403, 25563T	North America	0.72	Colombia	04/04/2020	USA	14/03/2020
South America	A1a1a3a	241, 1059, 3037, 11916, 14408, 18998, 23403, 25563T, 29540	North America	1.08	Argentina	28/03/2020	USA	05/03/2020
South America	A1a1+8389	241, 3037, 8389, 14408, 23403, 25563T	North America	6.83	Chile	12/03/2020	USA	02/03/2020
South Asia	A	0	East Asia	2.31	India	27/01/2020	China	24/12/2019
South Asia	A5	11083T	East Asia	39.19	India	16/03/2020	China	18/01/2020
South Asia	A5b	1397, 11083T, 28688, 29742T	East Asia	1.15	India	10/03/2020	China	18/01/2020
South Asia	A2a1a1	2480, 2558, 11083T, 14805, 26144T	Europe	0.58	India	15/03/2020	England	09/02/2020
South Asia	A1a	241, 3037, 14408, 23403	Europe	20.17	India	11/03/2020	Italy	20/02/2020

South Asia	A1a9a	241, 3037, 4255T, 14408, 23403, 26530	Europe	0.58	India	17/03/2020	USA	05/03/2020
South Asia	A1a2	241, 3037, 14408, 23403, 28881, 28882	Europe	3.75	Sri Lanka	10/03/2020	England	23/02/2020
South Asia	A1a2d	241, 313, 3037, 14408, 23403, 28881, 28882	Europe	1.73	India	02/03/2020	Switzerland	27/02/2020
South Asia	A1a1a	241, 1059, 3037, 14408, 23403, 25563T	Europe	0.58	India	13/03/2020	France	21/02/2020
South Asia	A1a1a+ 28371T	241, 1059, 3037, 14408, , 23403, 25563T, 28371T	Europe	0.58	India	13/04/2020	Wales/Russia	27/03/2020
South Asia	B7+22468T	8782, 22468T, 28144, 28878, 29742	North America	2.02	India	26/03/2020	USA	06/03/2020
South Asia	A5b+884, 8653T	884, 1397, 8653T, 11083T, 28688, 29742T	Western Asia	5.19	India	10/03/2020	Norway	26/02/2020
South Asia	A1a1b1	241, 3037, 14408, 18877, 23403, 25563T, 26735	Western Asia	8.36	India	20/04/2020	Saudi Arabia	10/03/2020
South Asia	A1a1b1+22444, 28854	241, 3037, 14408, 18877, 22444, 23403, 25563T, 26735, 28854	Western Asia	10.37	India	15/03/2020	India	15/03/2020
Western Asia	B7	8782, 28144, 28878, 29742	China	0.67	Saudi Arabia	29/03/2020	China	23/01/2020
Western Asia	A2a	11083T, , 26144T	East Asia	0.89	Georgia	27/02/2020	China	23/01/2020
Western Asia	A5b	1397, 11083T, 28688, 29742T	East Asia	11.97	United Arab Emirates	25/02/2020	China	18/01/2020

Western Asia	A2a1c	11083T, 14805, 26144T, 28842T	Europe	1.33	Jordan	16/03/2020	Denmark	09/03/2020
Western Asia	A2a1	11083T, 14805, 26144T,	Europe	2.00	Qatar	23/03/2020	Korea	18/02/2020
Western Asia	A2a1b	11083T, 14805, 17247, 26144T	Europe	1.33	Israel	17/03/2020	England	25/02/2020
Western Asia	A2a1b3	7479, 11083T, 14805, 17247, 25572, 26144T, 28887	Europe	0.44	Jordan	30/03/2020	Scotland	17/03/2020
Western Asia	A1a3	241, 3037, 14408, 20268, 23403	Europe	0.44	Georgia	16/03/2020	Switzerland	27/02/2020
Western Asia	A1a	241, 3037, 14408, 23403	Europe	3.33	Georgia	10/03/2020	Italy	20/02/2020
Western Asia	A1a5	241, 3037, 14408, 15324, 23403	Europe	0.67	Kuwait	15/03/2020	France/Thailand	03/03/2020
Western Asia	A1a6	241, 3037, 14408, 23403, 25350	Europe	0.67	Israel	02/04/2020	Australia	22/02/2020
Western Asia	A1a+1059	241, 1059, 3037, 14408, 23403	Europe	0.44	Israel	23/03/2020	Netherlands	09/03/2020
Western Asia	A1a2	241, 3037, 14408, 23403, 28881, 28882	Europe	6.21	Israel	01/03/2020	England	23/02/2020
Western Asia	A1a2d	241, 313, 3037, 14408, 23403, 28881, 28882	Europe	3.99	Israel	01/03/2020	Switzerland	27/02/2020
Western Asia	A1a1c	241, 2416, 3037, 14408, 23403, 25563T	Europe	3.99	Israel	17/03/2020	France	29/02/2020
Western Asia	A1a1a	241, 1059, 3037, 14408, 23403, 25563T	Europe	10.86	United Arab Emirates	10/03/2020	France	21/02/2020
Western Asia	A1a1a5a	241, 1059, 3037, 13006, 14408,	Europe	0.44	Israel	29/03/2020	Senegal	28/02/2020

		23403, 25563T, 25688						
Western Asia	A1a1	241, 3037, 14408, 23403, 25563T	Europe	3.77	Lebanon	13/03/2020	France	26/02/2020
Western Asia	A1a1a14	241, 1059, 11083T, 14408, 23403, 25563T	Europe	0.44	Trukey	26/03/2020	Czech Republic	08/03/2020
Western Asia	B7+22468T	8782, 22468T, 28144, 28878, 29742	North America	4.66	Saudi Arabia	23/03/2020	USA	06/03/2020
Western Asia	A1a1b	241, 3037, 14408, 18877, 23403, 25563T	North America	27.27	Lebanon	04/03/2020	USA/Canada	29/02/2020
Western Asia	A1a1a+16616	241, 1059, 3037, 14408, 16616, 23403, 25563T	North America	0.44	Turkey	26/03/2020	USA	19/03/2020
Western Asia	A1a1a3a	241, 1059, 3037, 11916, 14408, 18998, 23403, 25563T, 29540	North America	6.43	Israel	26/03/2020	USA	05/03/2020
Western Asia	A1a1a+27549	241, 1059, 3037, 14408, 23403, 25563T, 27549	North America	0.67	Israel	26/03/2020	USA	09/03/2020
Western Asia	A1a1a8	241, 1059, 3037, 14408, 20755C, 23403, 25563T	North America	3.10	Israel	17/03/2020	Australia	09/03/2020
Western Asia	A1a1a22	241, 1059, 1917, 3037, 14408, 23403, 25563T	North America	0.44	Isreal	20/04/2020	USA	14/03/2020

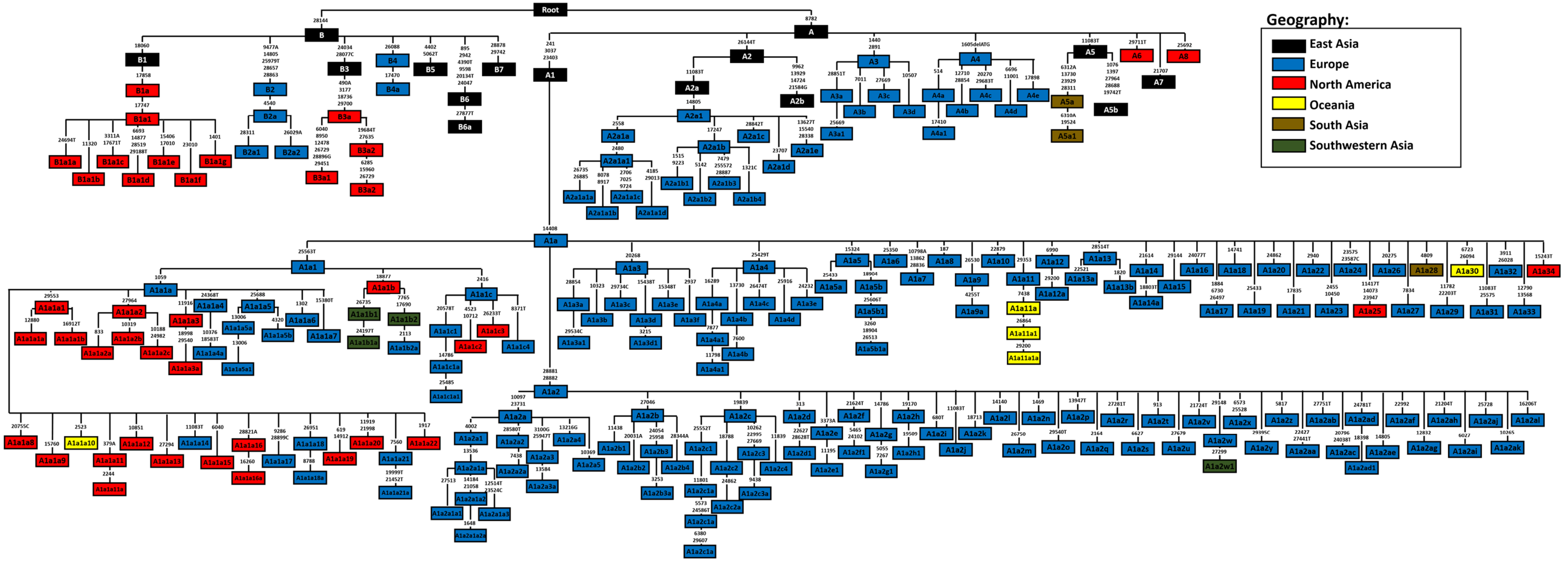

Figure S1. Schematic representation of the main branches in SARS-CoV-2 genomic diversity worldwide. Mutations are labelled considering the positions in the first SARS-CoV-2 genome. Position is followed by A, C, T or G only when the mutation is a transversion. Each clade is colored according the most probable place of origin of each clade. The nomenclature was developed for the purpose of this study following principles (using intercalating numbers and letters) developed for human mitochondrial DNA. One deletion (1605delATG) was important to define an epidemiological relevant clade and it was used together with the substitutions. Founders are evident at each point where there is a color change on the tree, moving away from the root.

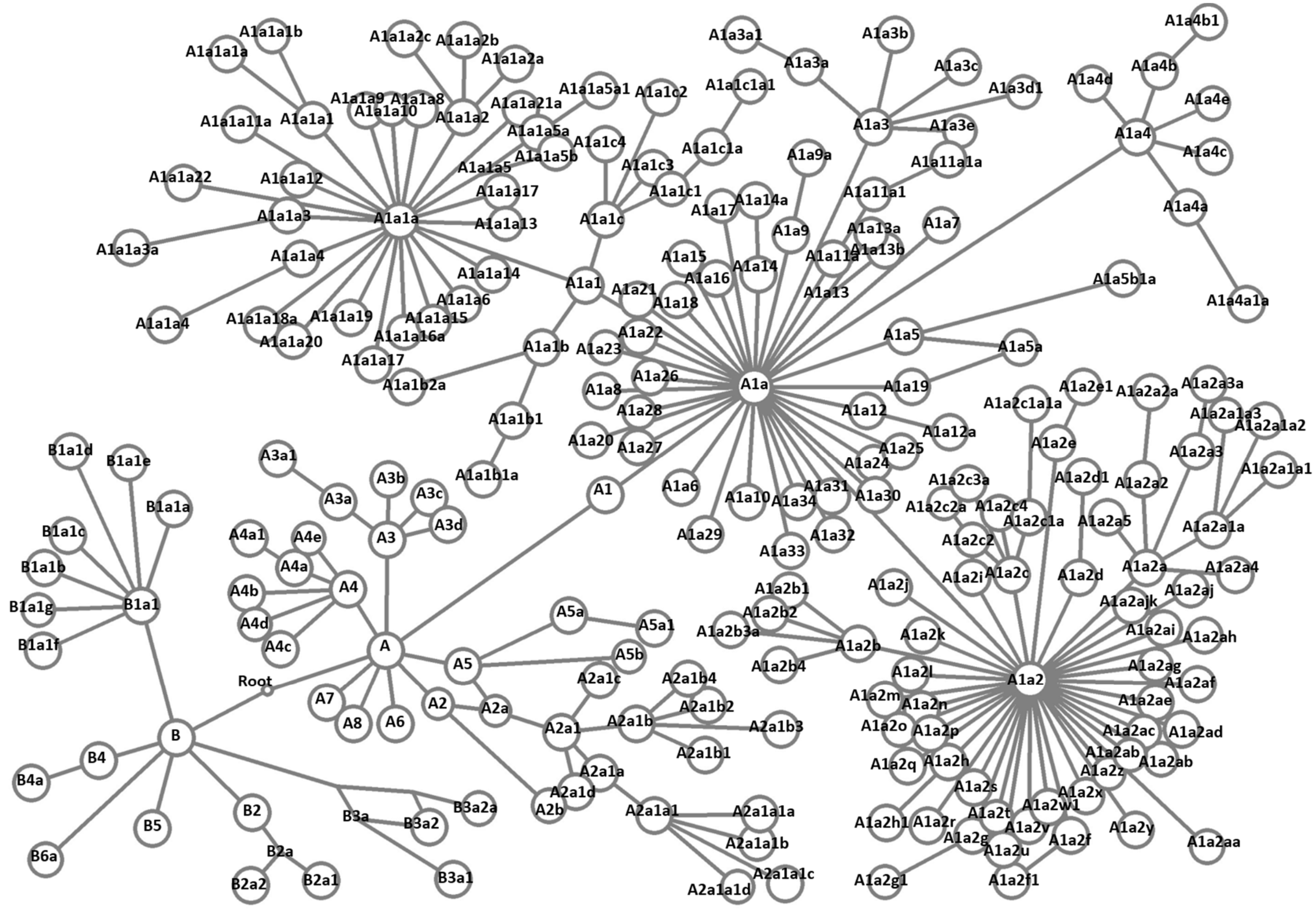

Figure S2. Reduced-median network of SARS-CoV-2 variation worldwide using only haplotypes that were detected at least ten times in the global database. This network provides the link between Figure 1 and the nomenclature displayed in Figure S1.

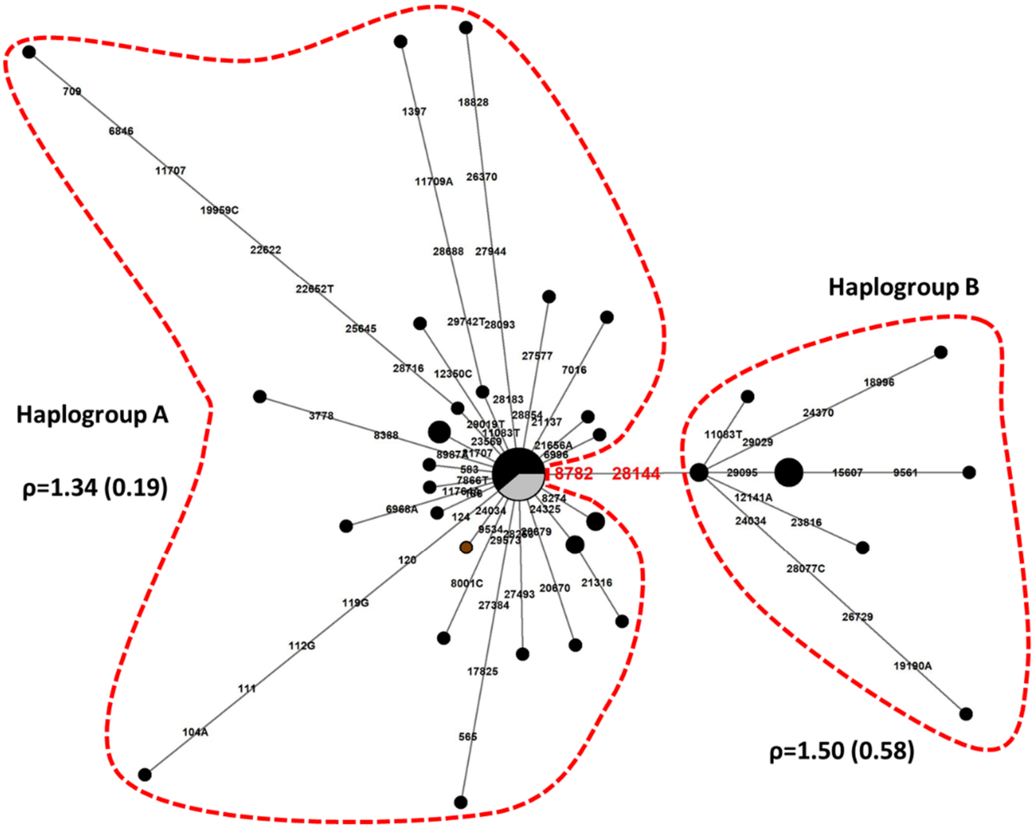

Figure S3. Reduced-median network displaying the accumulated genomic diversity of the virus in Asia (from 24th December 2019 to 18th January 2020) up until the appearance of the first sequenced case outside the continent. A diversity measure, ρ, was estimated for each major haplogroup A and B. Values between brackets indicate standard errors of the estimate. Samples in black are from China, samples in grey from Mainland Southeast Asia and samples in brown from South Asia.

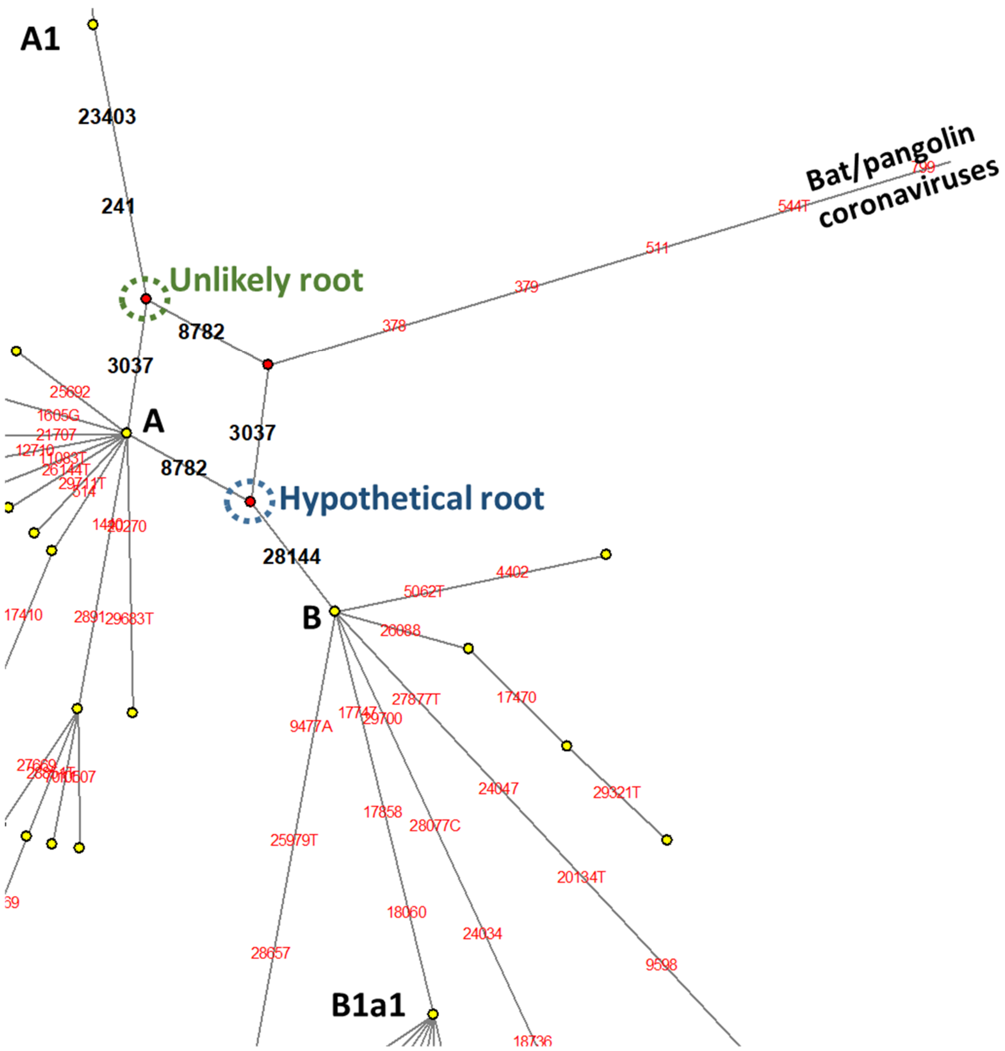

Figure S4. Portion of a reduced-median network showing the reticulation caused in the root of the tree with the three outgroups. Position 3037 is a fast site and likely to be involved in multiple occurrences, generating an "unlikely root". Haplogroups A, B, A1 and B1a1 are labelled according to Figure S1.

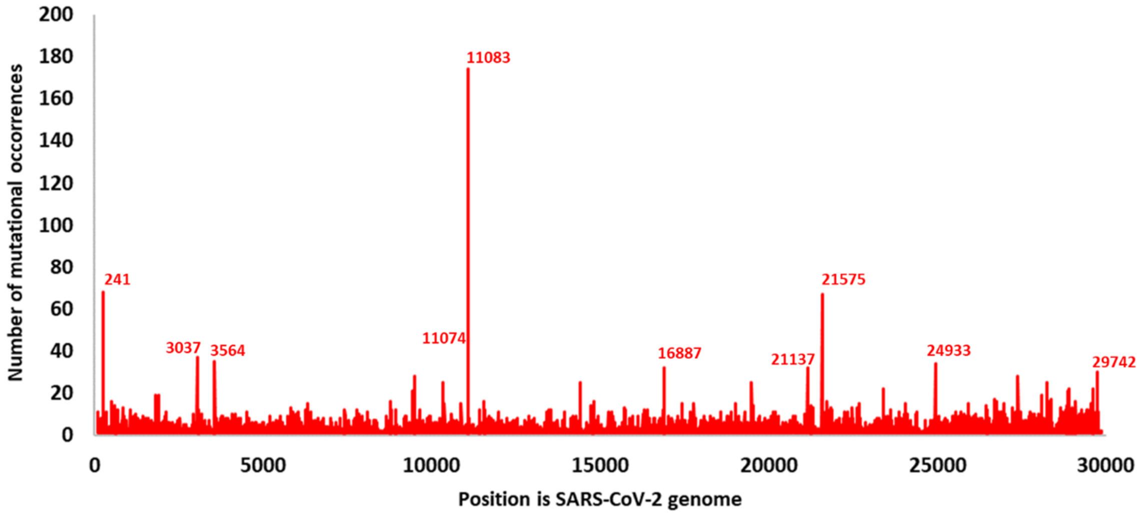

Figure S5. Mutational spectrum across the complete genome of SARS-CoV-2 based on 20247 detected mutations in the phylogenetic reconstruction. Positions that underwent more than 30 mutations are indicated.

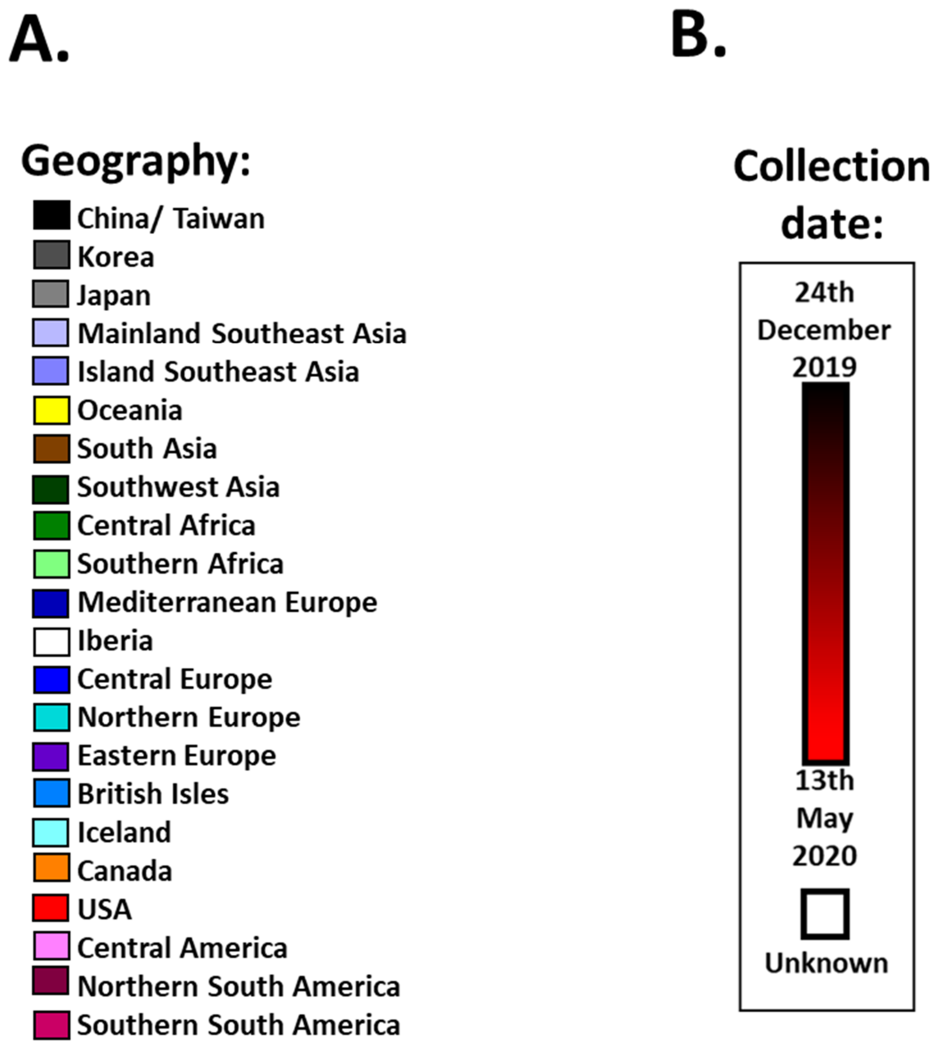

Figure S6. Color code to be employed throughout networks displayed in Figures S5 to S57, for: (A) geography and (B) time of collection.

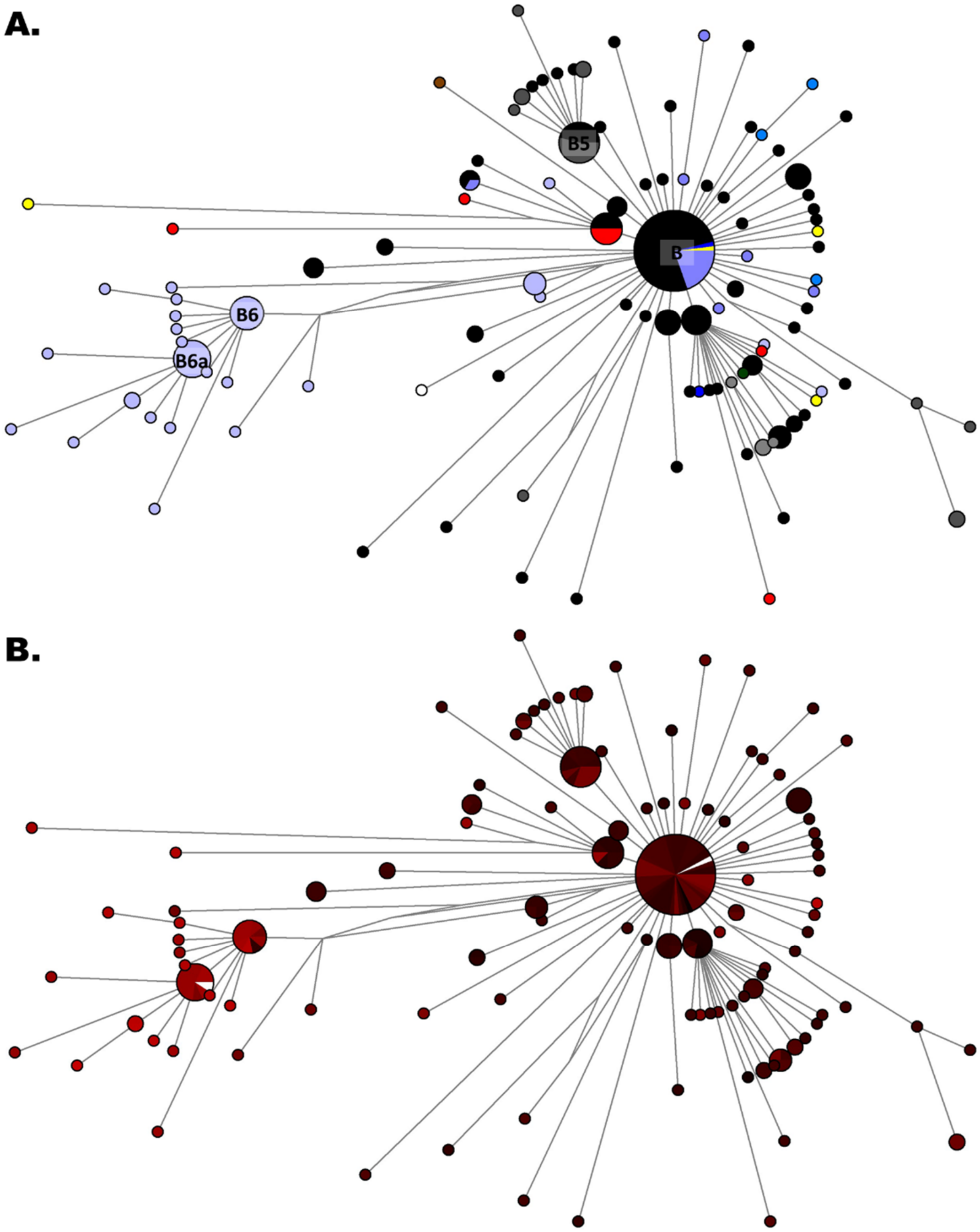

Figure S7. Reduced-median network of paragroup B (including B and B6). Samples are colored according to geography (A) and time of collection (B) following the legend in Figure S4.

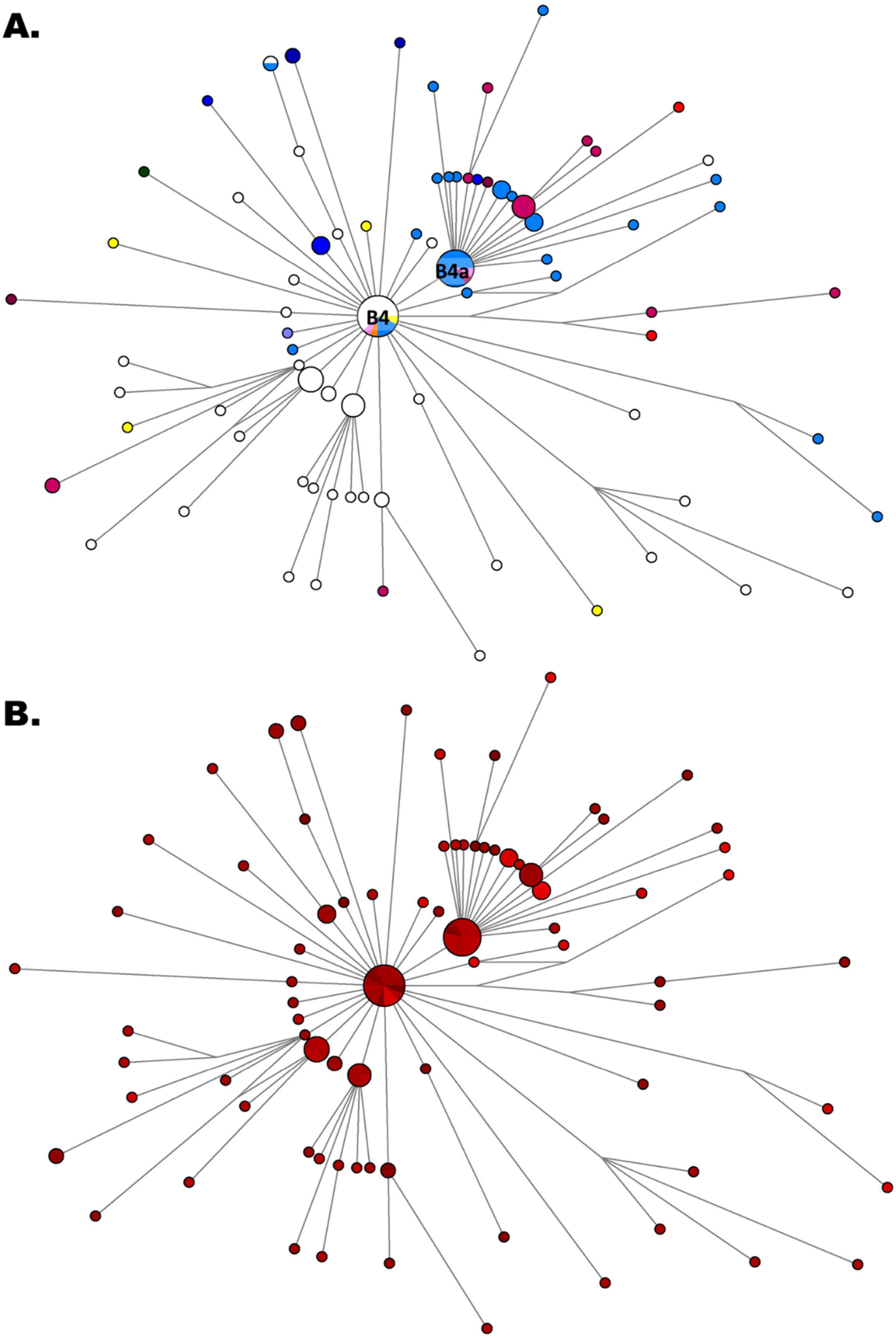

Figure S8. Reduced median network of clade B4. Samples are colored according to geography (A) and time of collection (B) following the legend in Figure S4.

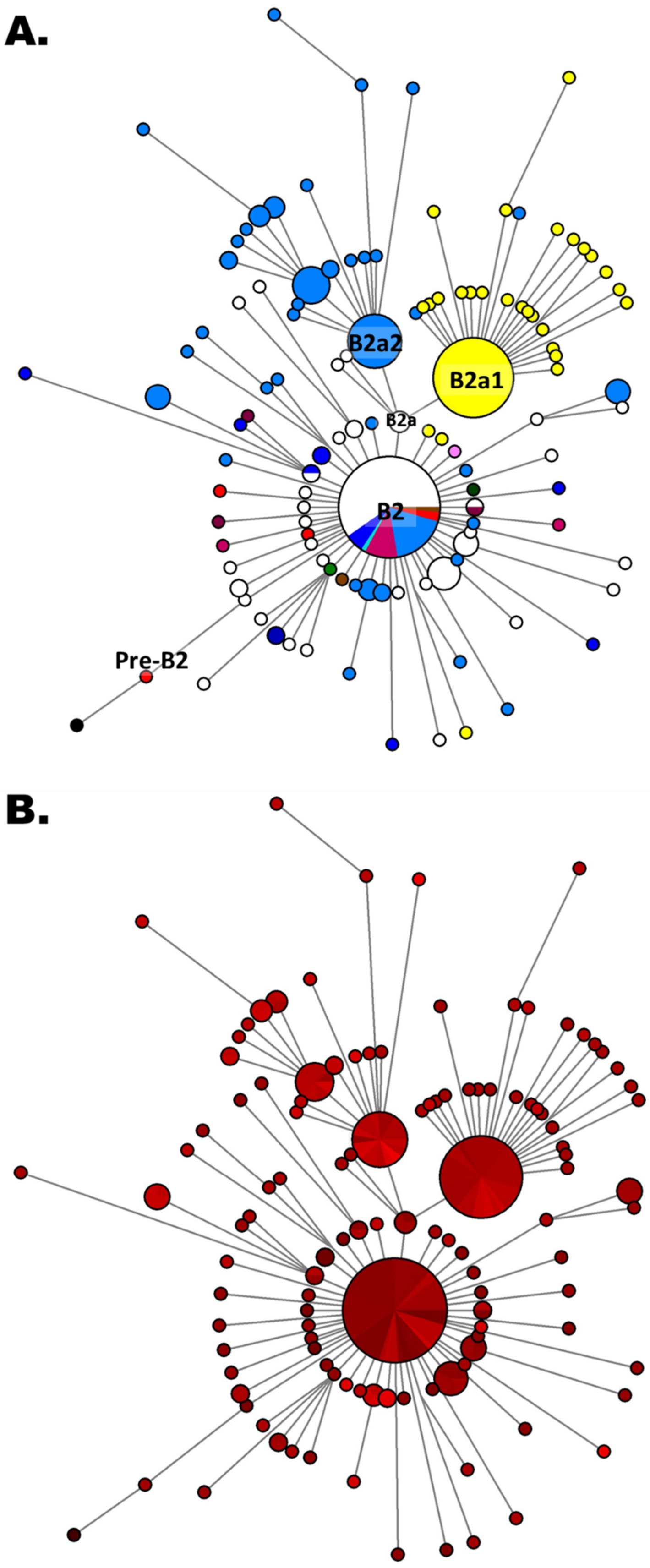

Figure S9. Reduced-median network of clade B2. Samples are colored according to geography (A) and time of collection (B) following the legend in Figure S4.

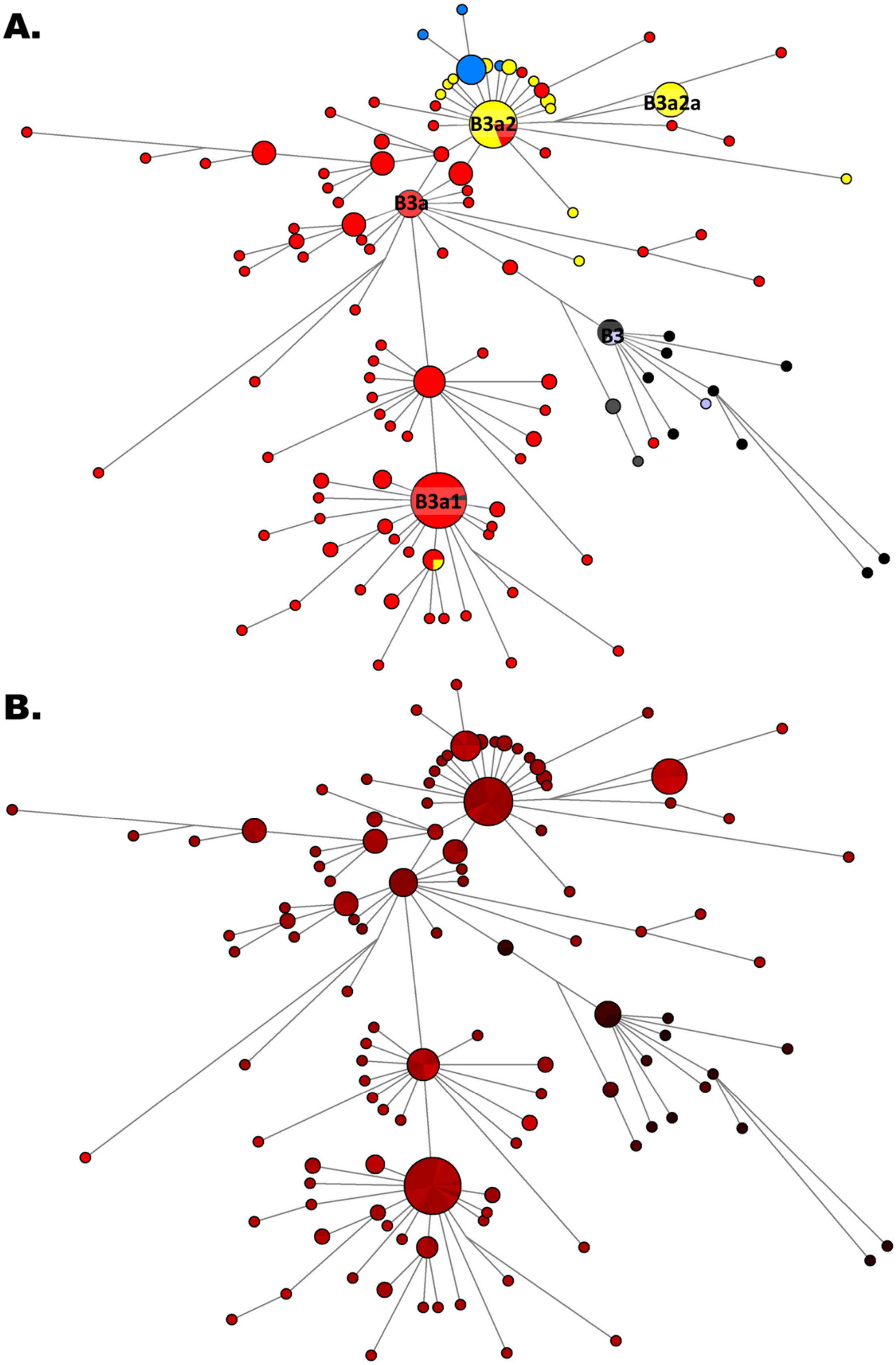

Figure S10. Reduced median network of clade B3. Samples are colored according to geography (A) and time of collection (B) following the legend in Figure S4.

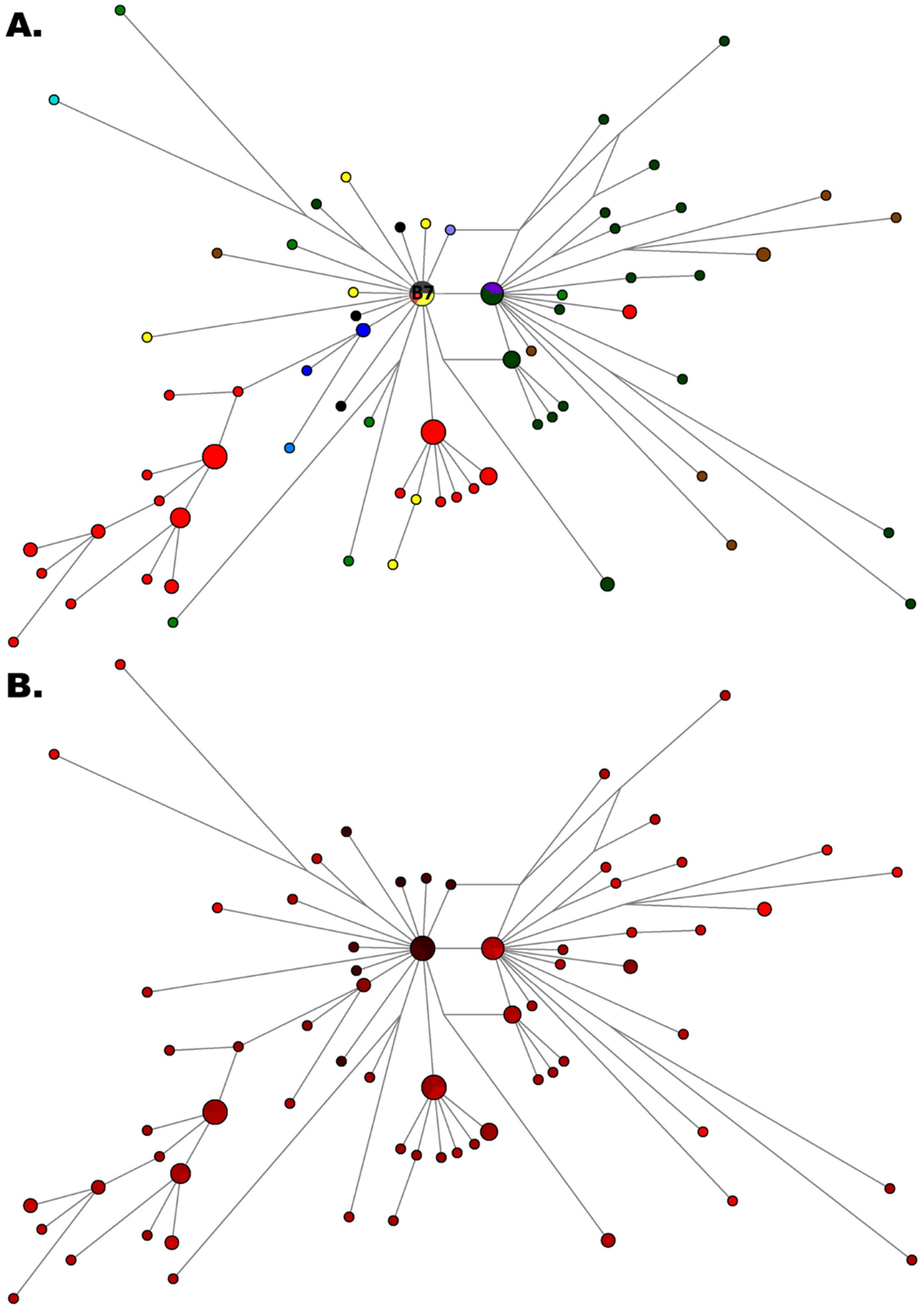

Figure S11. Reduced median network of clade B7. Samples are colored according to geography (A) and time of collection (B) following the legend in Figure S4.

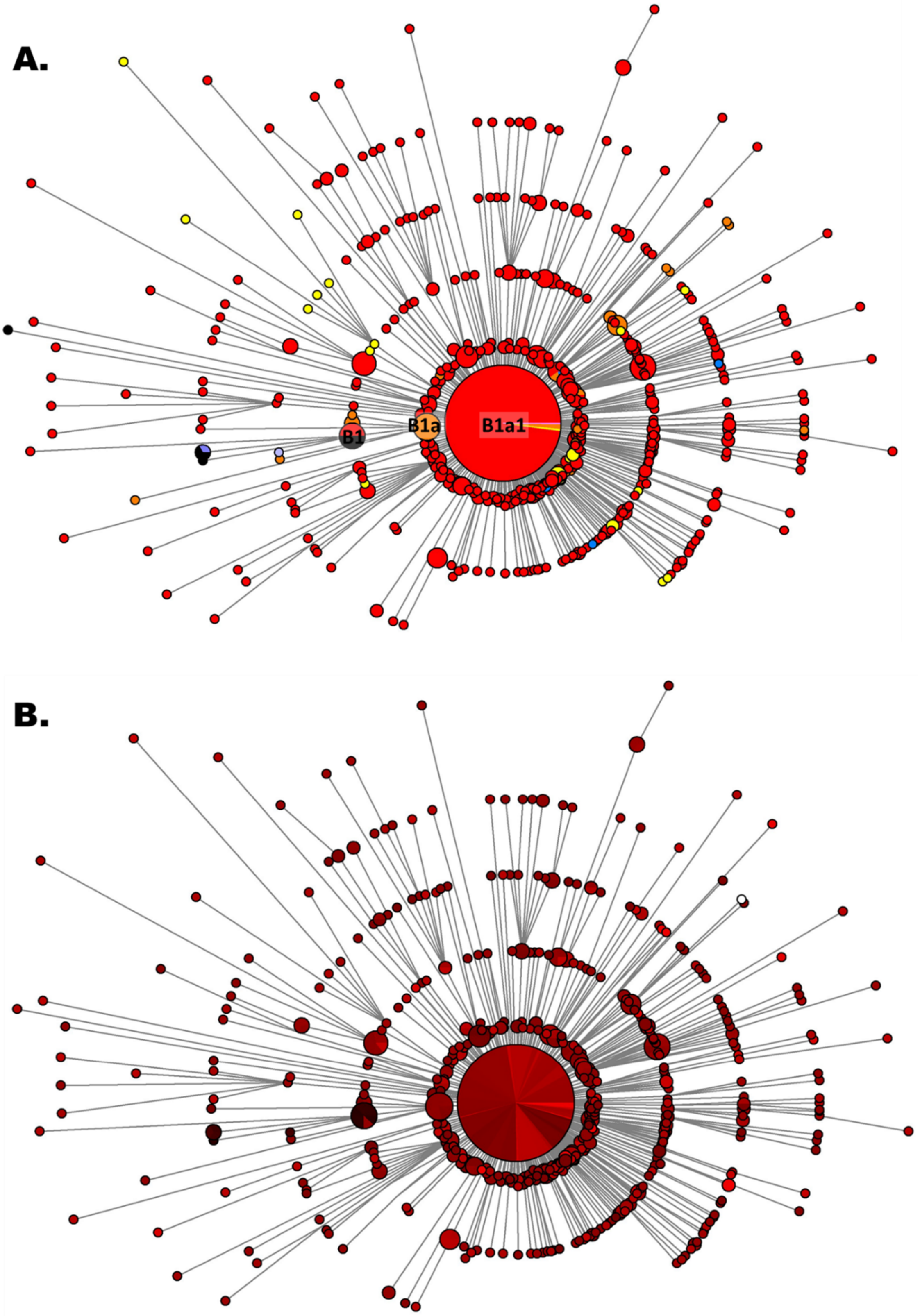

Figure S12. Reduced median network of clade B1 (containing B1a and B1a1). Some diversity of B1a1 are shown in Figure S11 and S12. Samples are colored according to geography (A) and time of collection (B) following the legend in Figure S4.

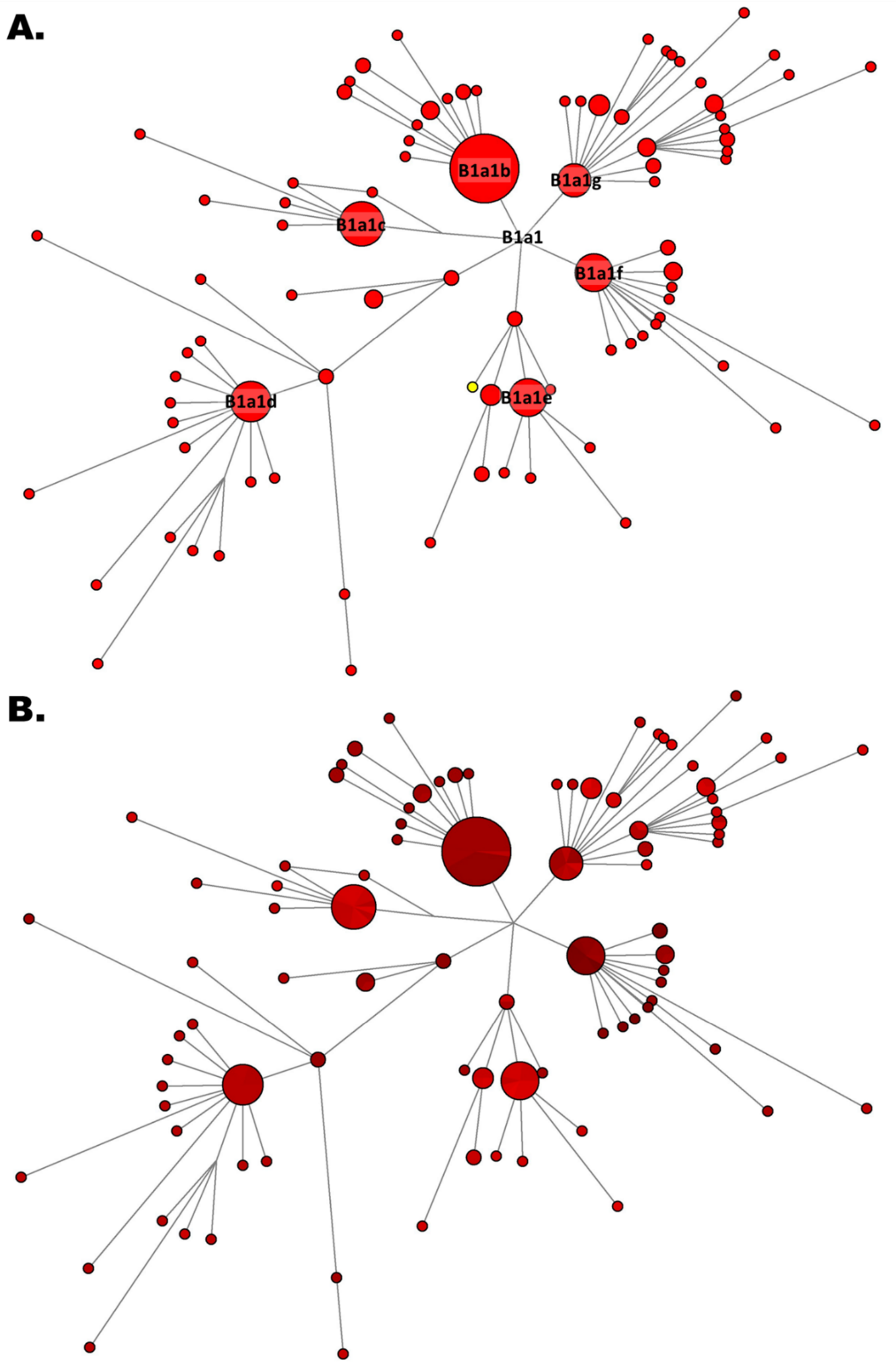

Figure S13. Reduced median network of subclades within B1a1. Samples are colored according to geography (A) and time of collection (B) following the legend in Figure S4.

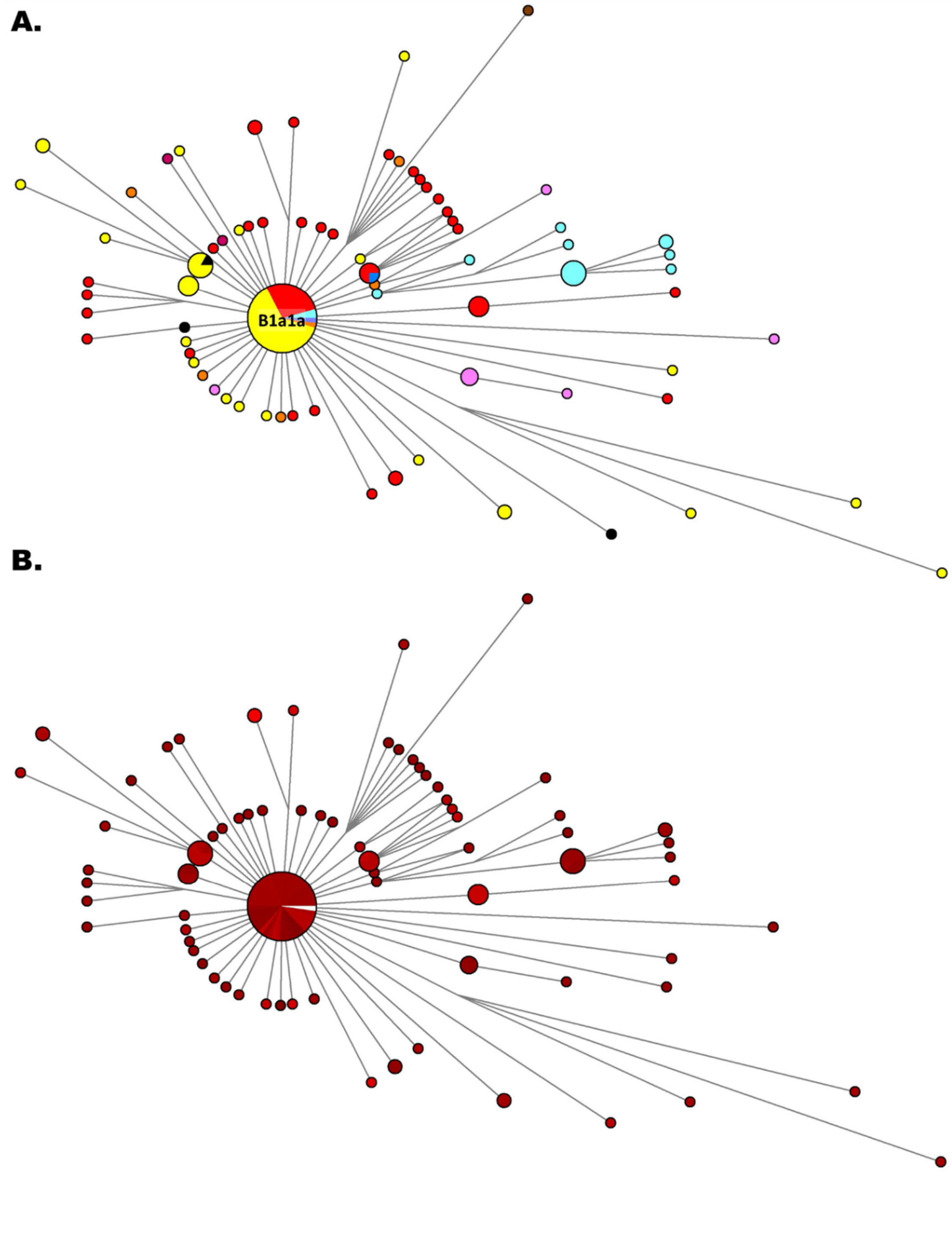

Figure S14. Reduced median network of clade B1a1a. Samples are colored according to geography (A) and time of collection (B) following the legend in Figure S4.

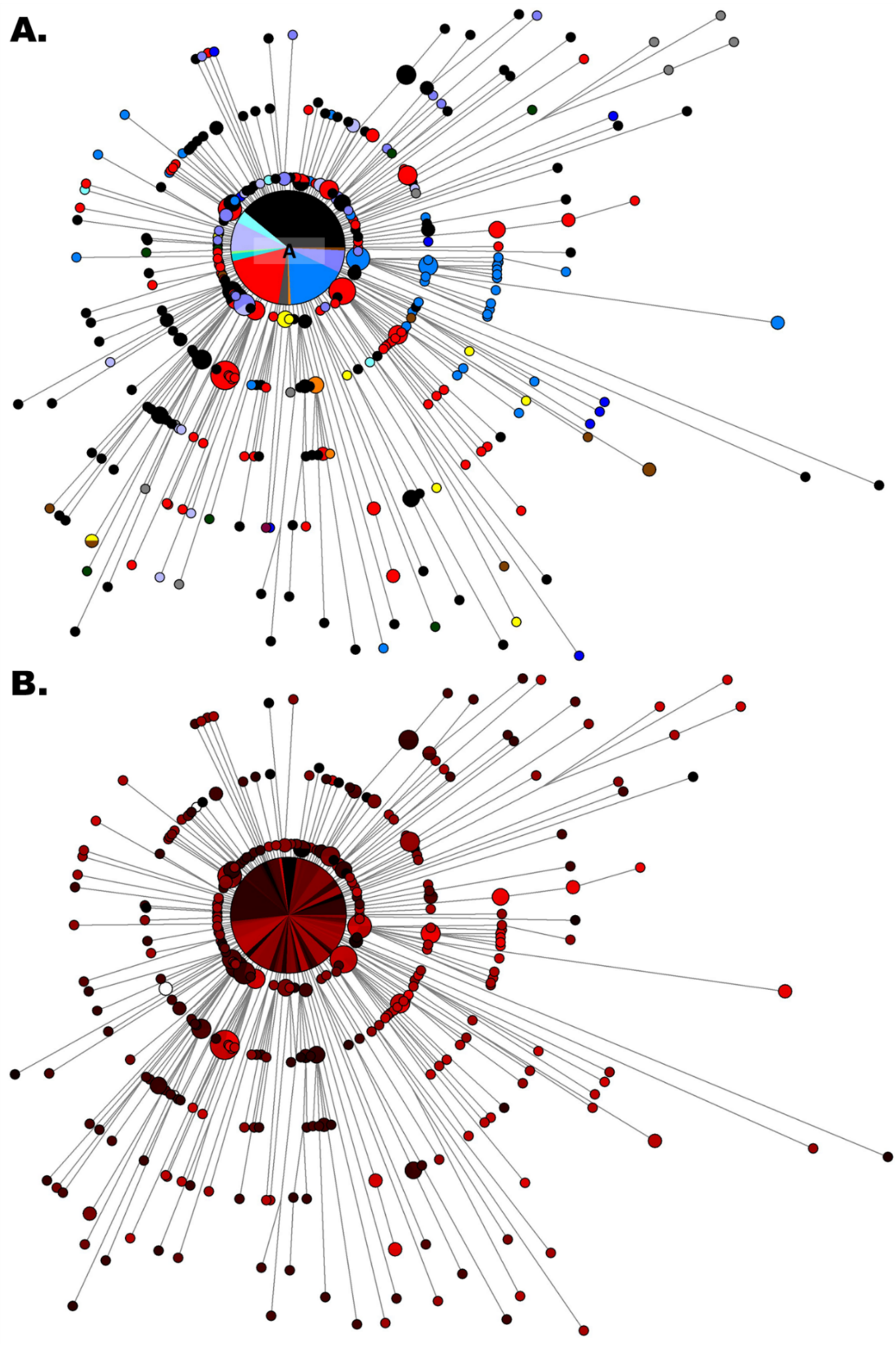

Figure S15. Reduced median network of paragroup A. Samples are colored according to geography (A) and time of collection (B) following the legend in Figure S4.

A.

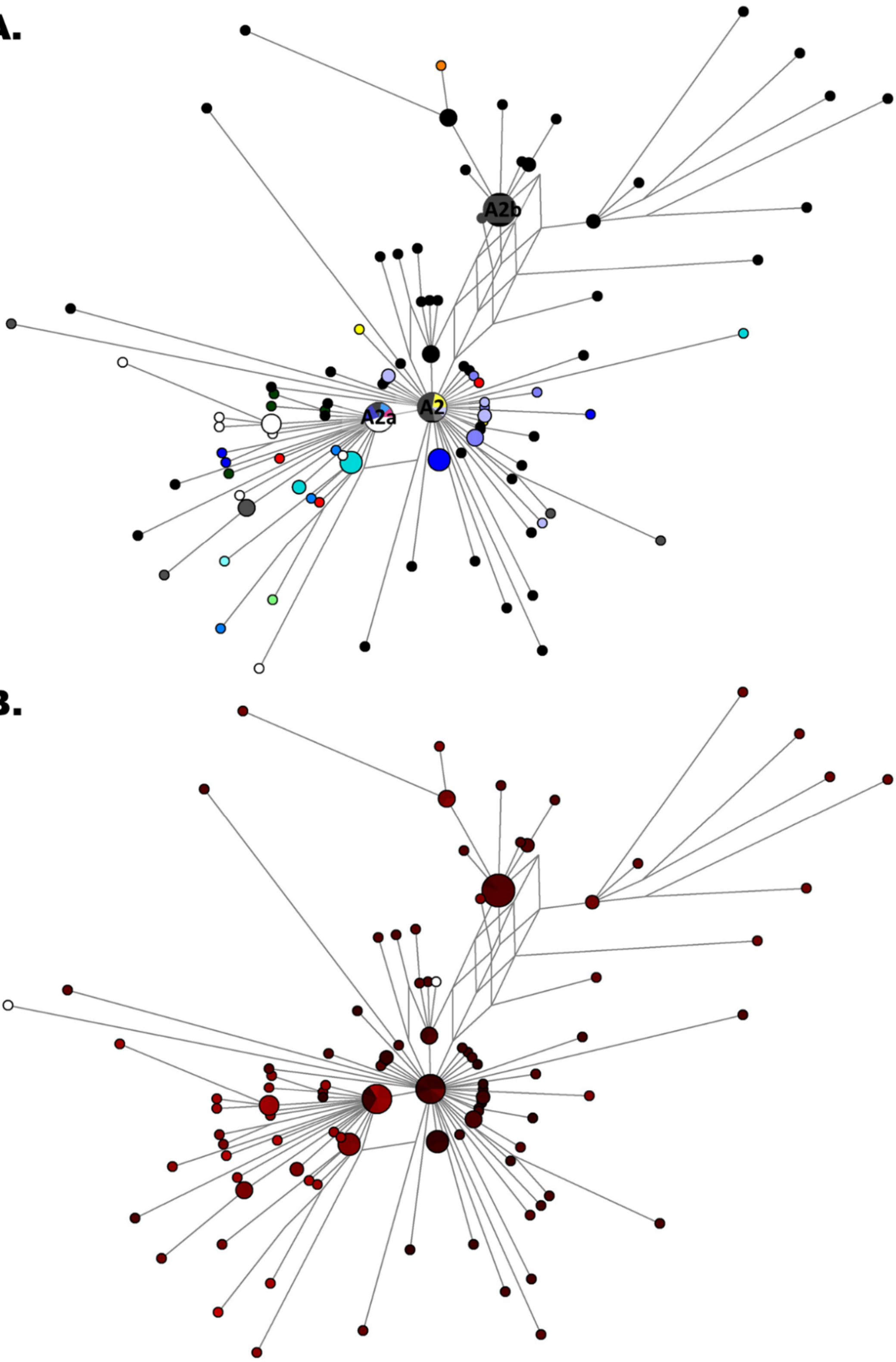

B.

Figure S16. Reduced median network of paragroup A2. Samples are colored according to geography (A) and time of collection (B) following the legend in Figure S4.

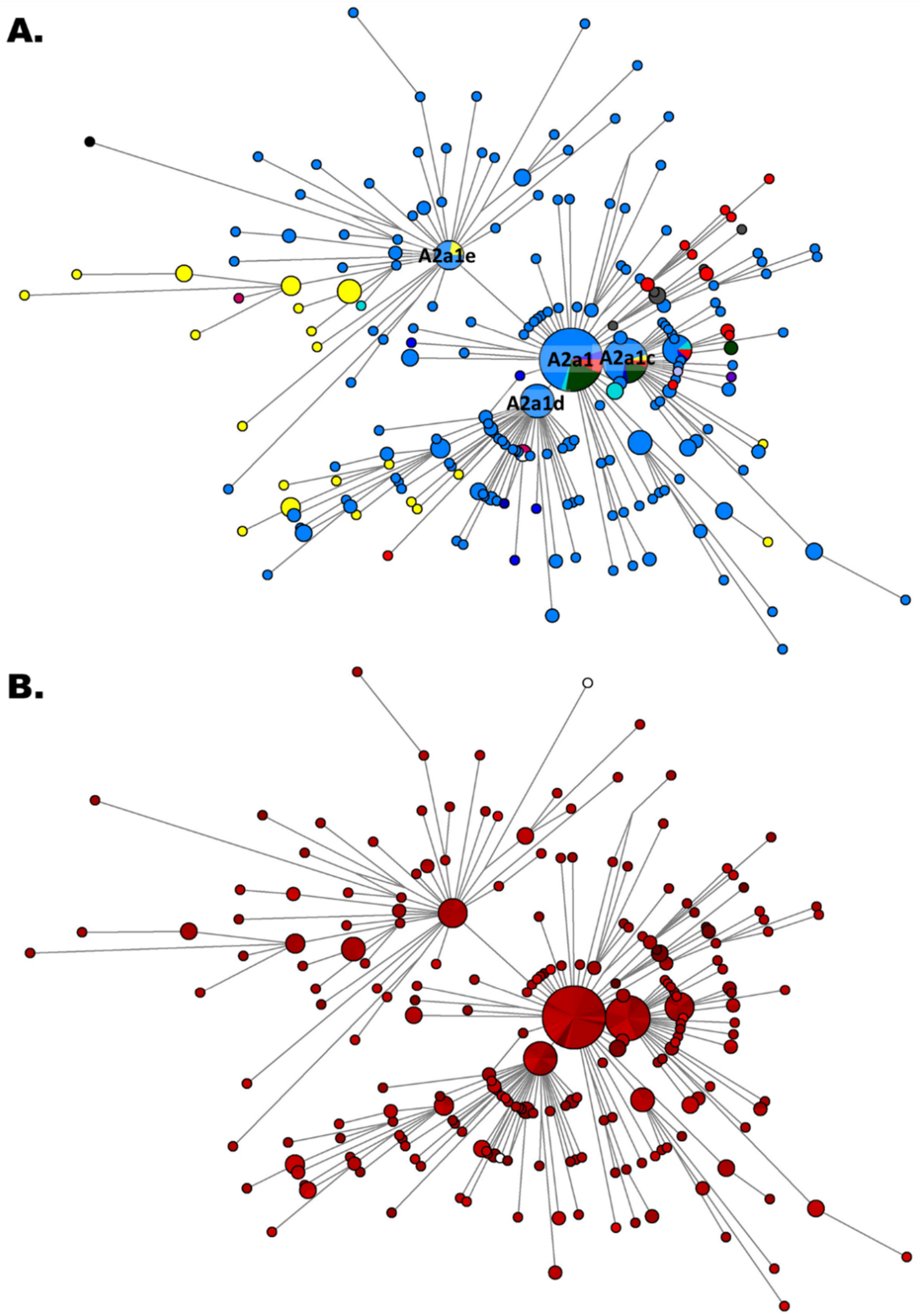

Figure S17. Reduced median network of paragroup A2a1. Samples are colored according to geography (A) and time of collection (B) following the legend in Figure S4.

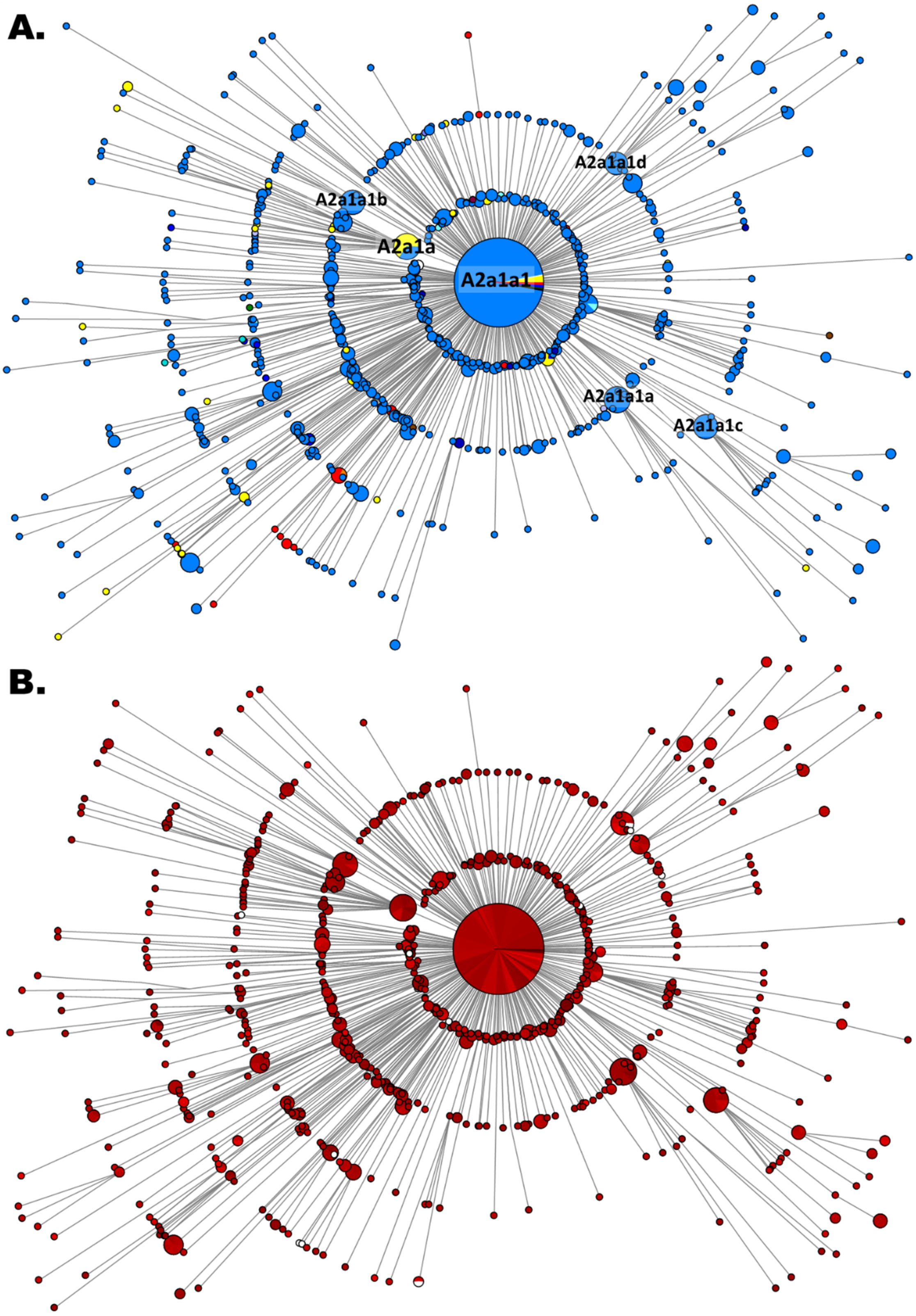

Figure S18. Reduced median network of clade A2a1a1. Samples are colored according to geography (A) and time of collection (B) following the legend in Figure S4.

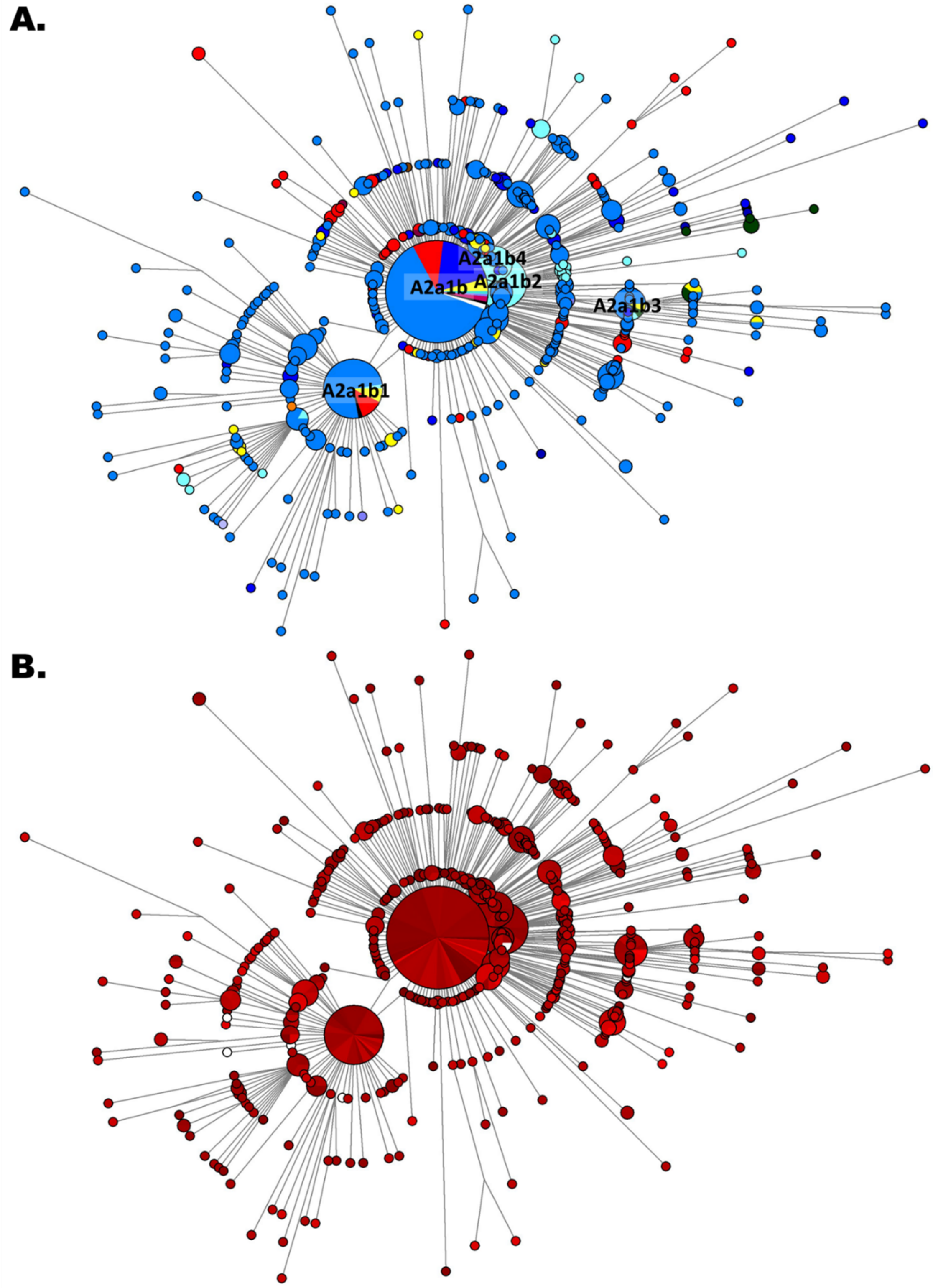

Figure S19. Reduced median network of clade A2a1b. Samples are colored according to geography (A) and time of collection (B) following the legend in Figure S4.

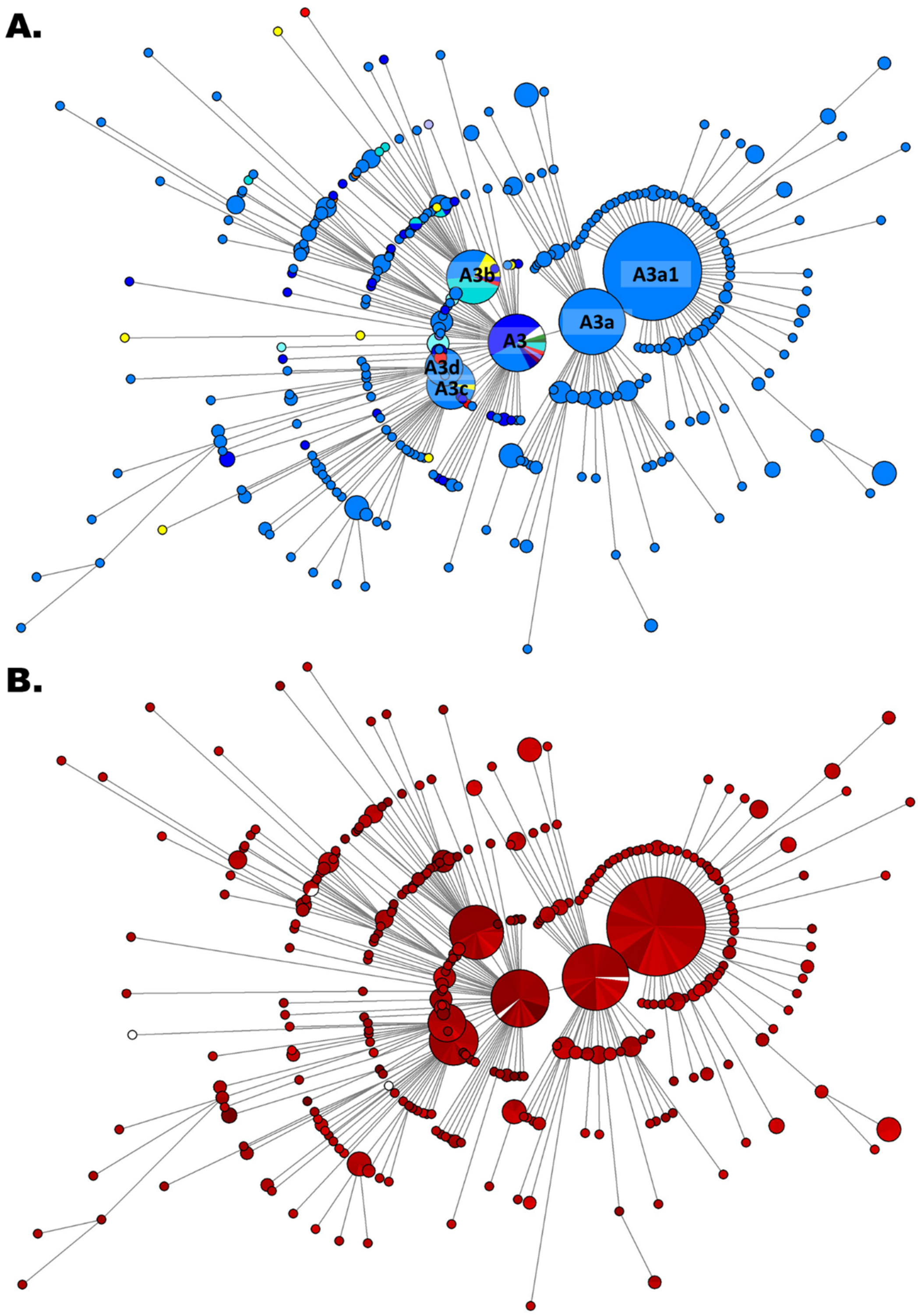

Figure S20. Reduced median network of clade A3. Samples are colored according to geography (A) and time of collection (B) following the legend in Figure S4.

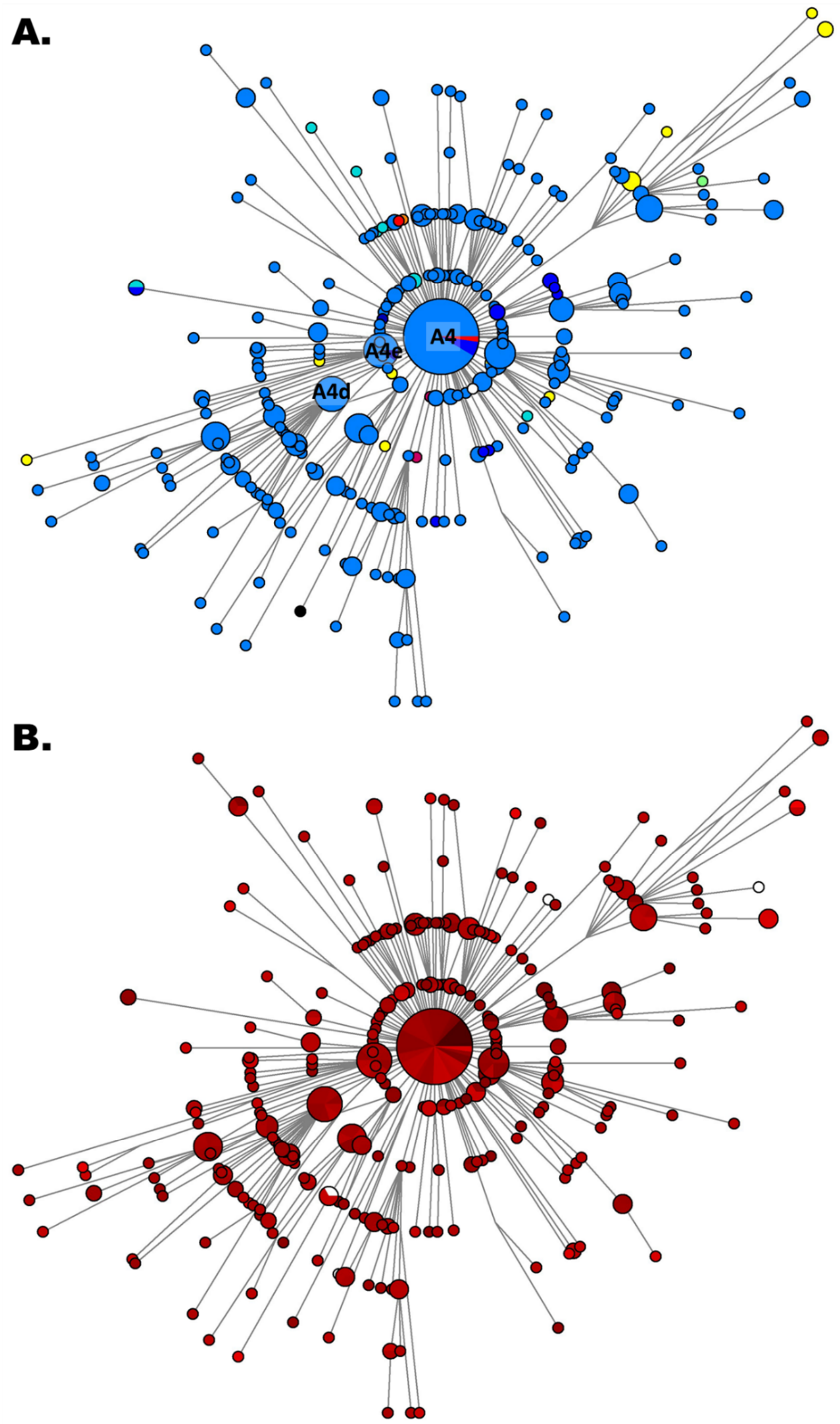

Figure S21. Reduced median network of paragroup A4. Samples are colored according to geography (A) and time of collection (B) following the legend in Figure S4.

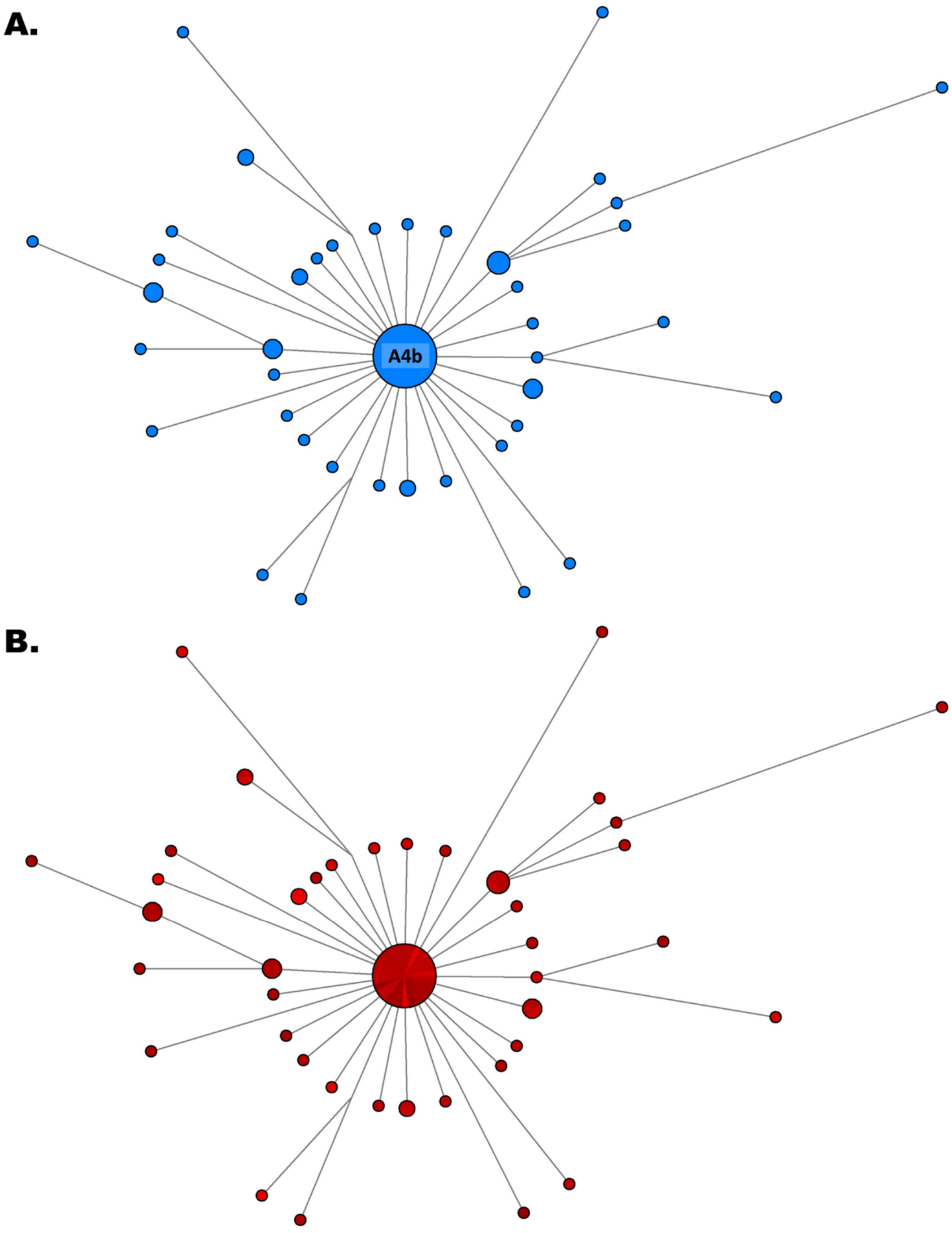

Figure S22. Reduced median network of clade A4b. Samples are colored according to geography (A) and time of collection (B) following the legend in Figure S4.

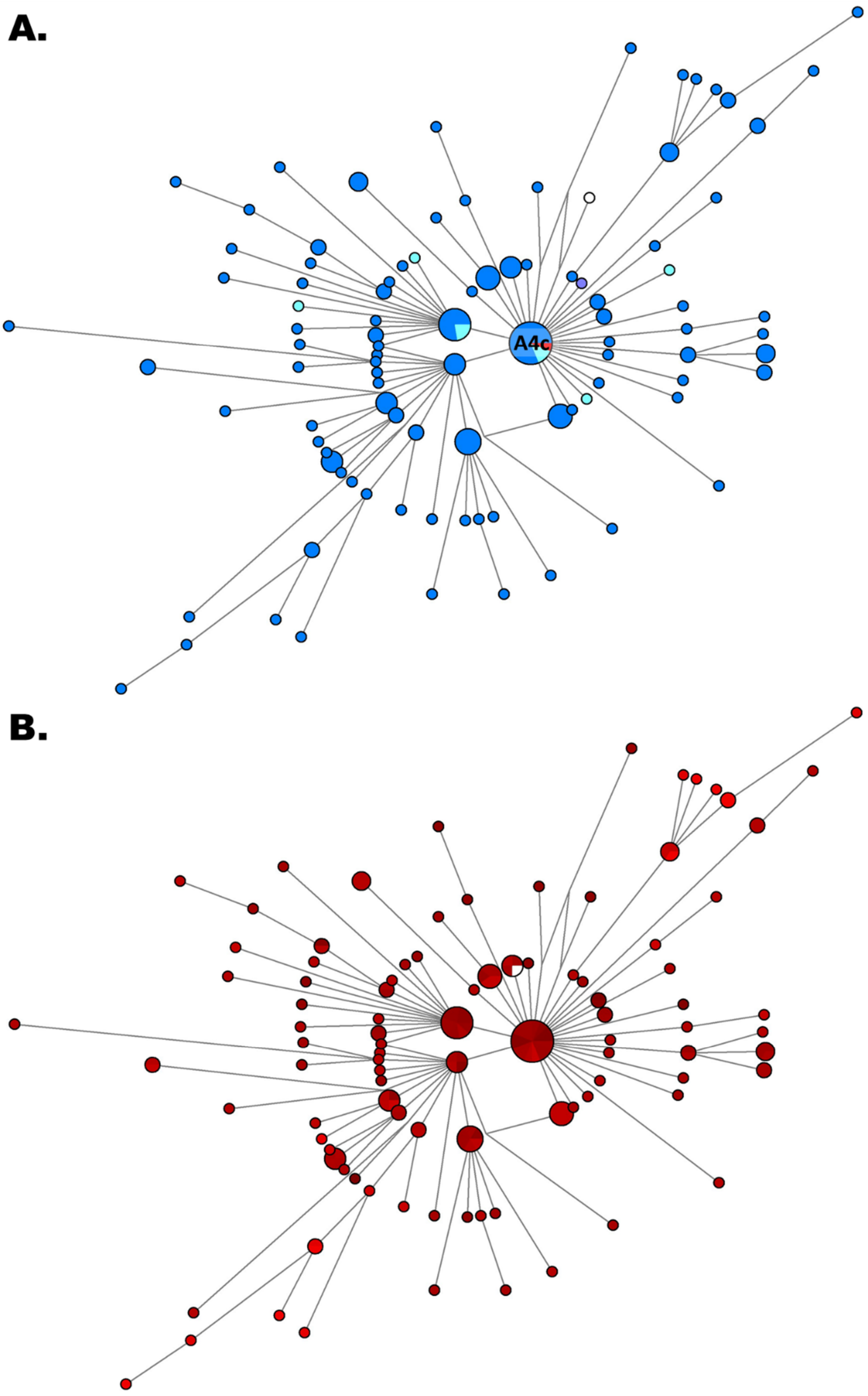

Figure S23. Reduced median network of clade A4c. Samples are colored according to geography (A) and time of collection (B) following the legend in Figure S4.

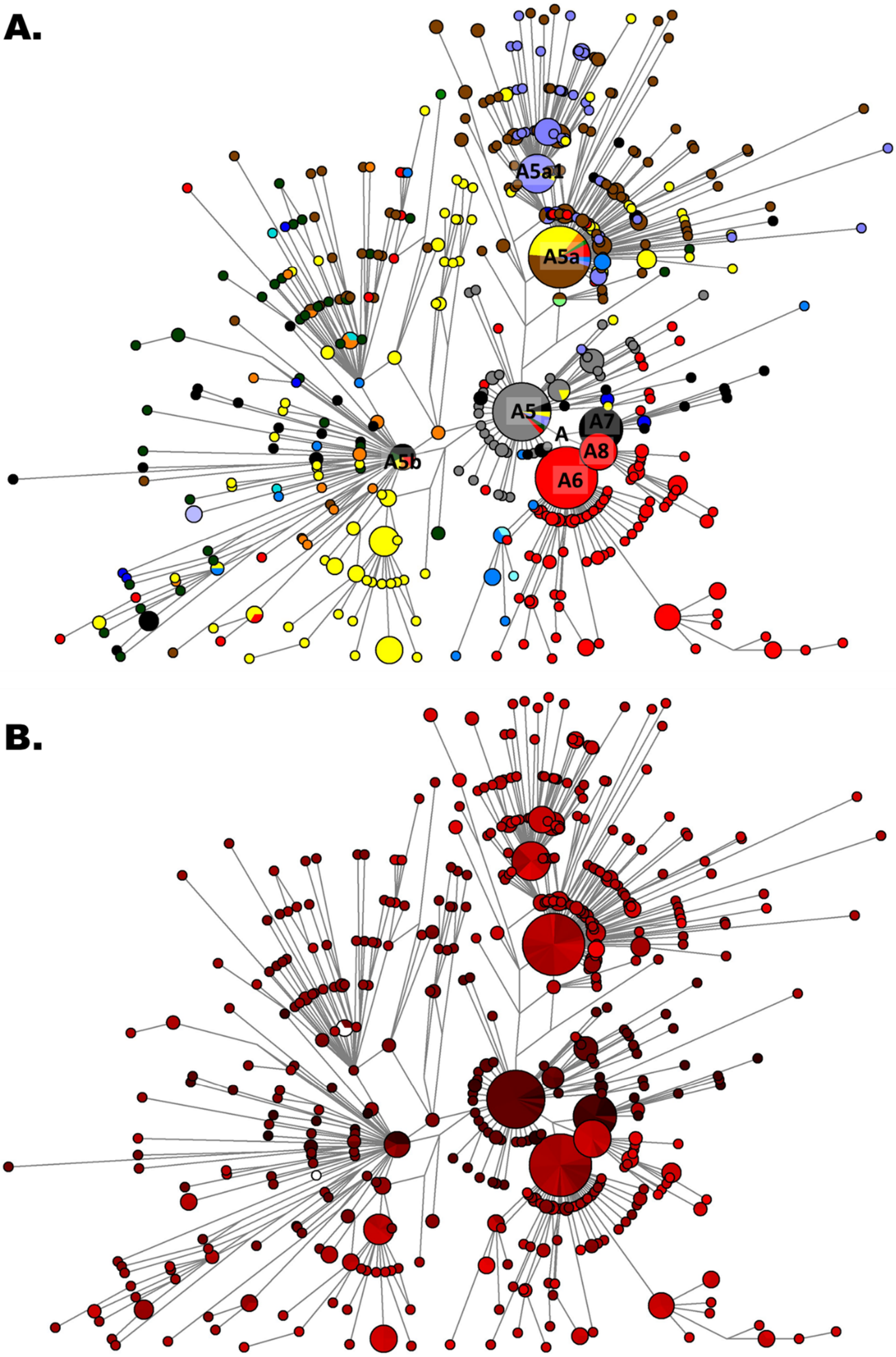

Figure S24. Reduced median network of subclade of A: A5, a6, A7 and A8. Samples are colored according to geography (A) and time of collection (B) following the legend in Figure S4.

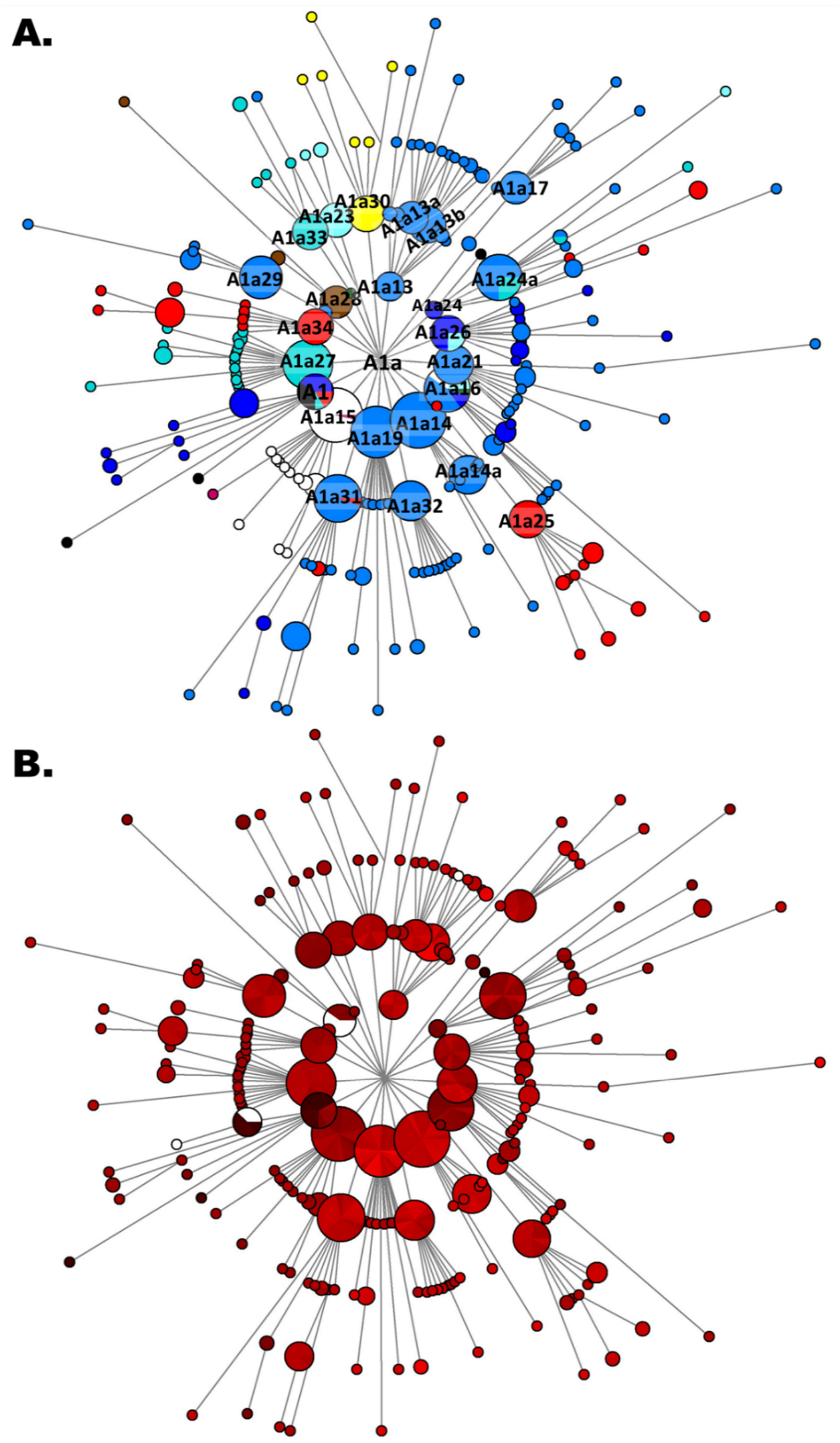

Figure S25. Reduced median network of paragroup A1 and subclade of A1a. Samples are colored according to geography (A) and time of collection (B) following the legend in Figure S4.

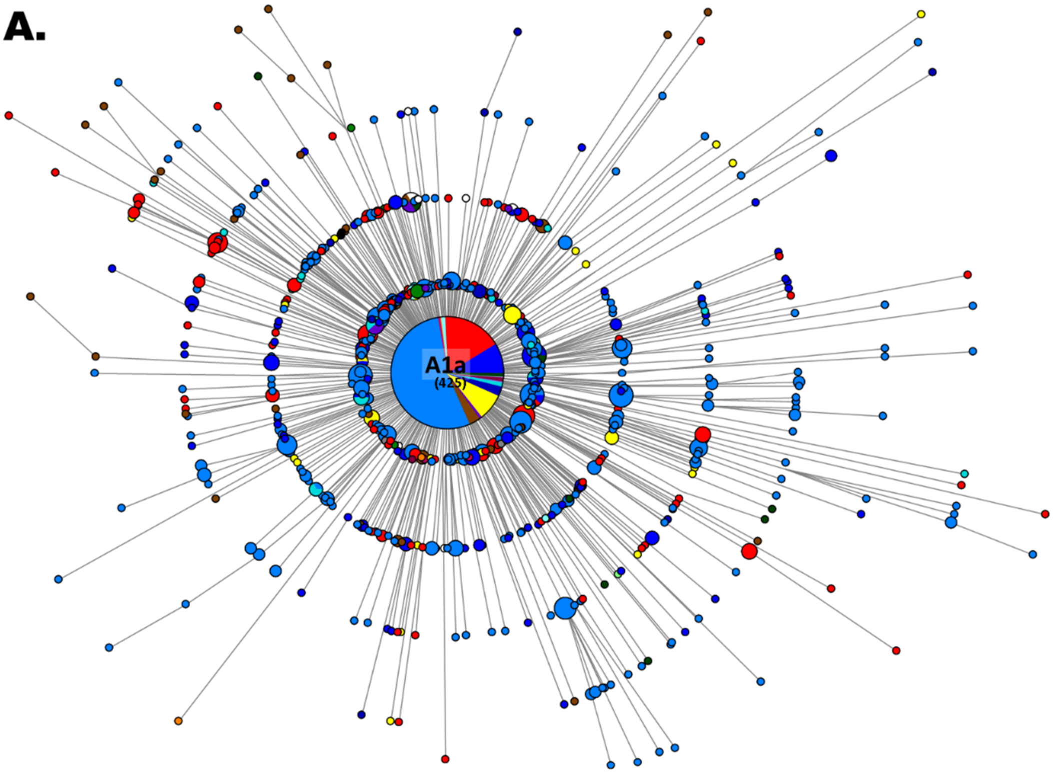

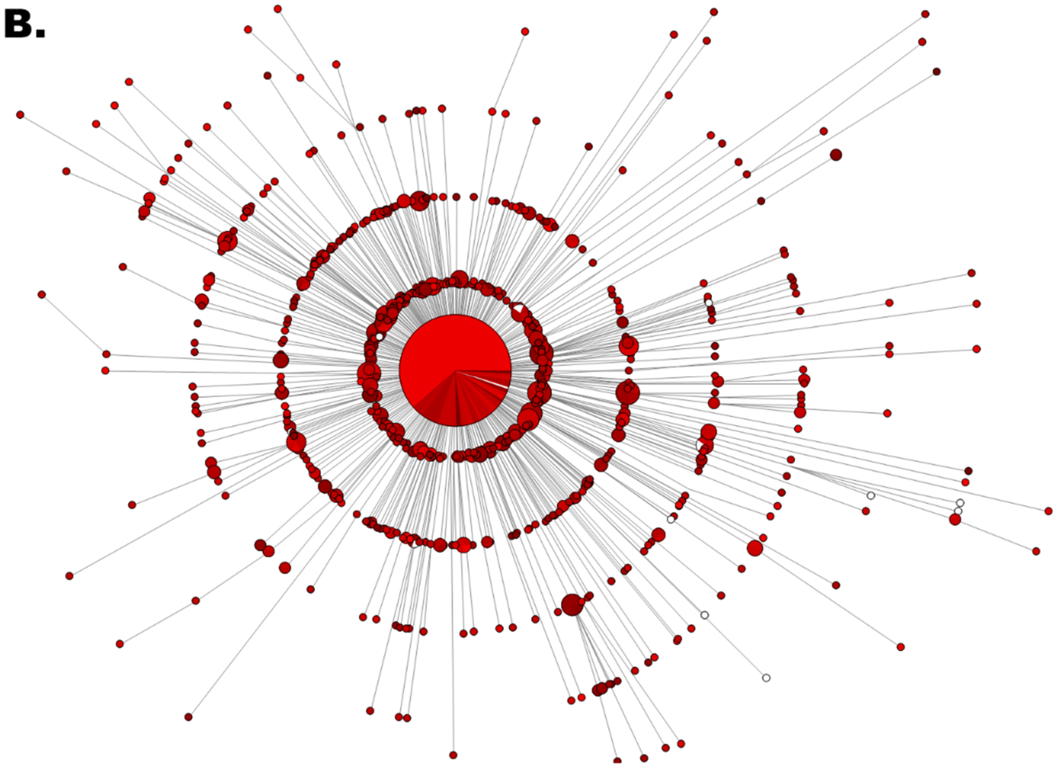

Figure S26. Reduced median network of paragroup A1a. The value between brackets refers to the total number of samples in the largest haplotype that is not proportional to the sample size. Samples are colored according to geography (A) and time of collection (B) following the legend in Figure S4.

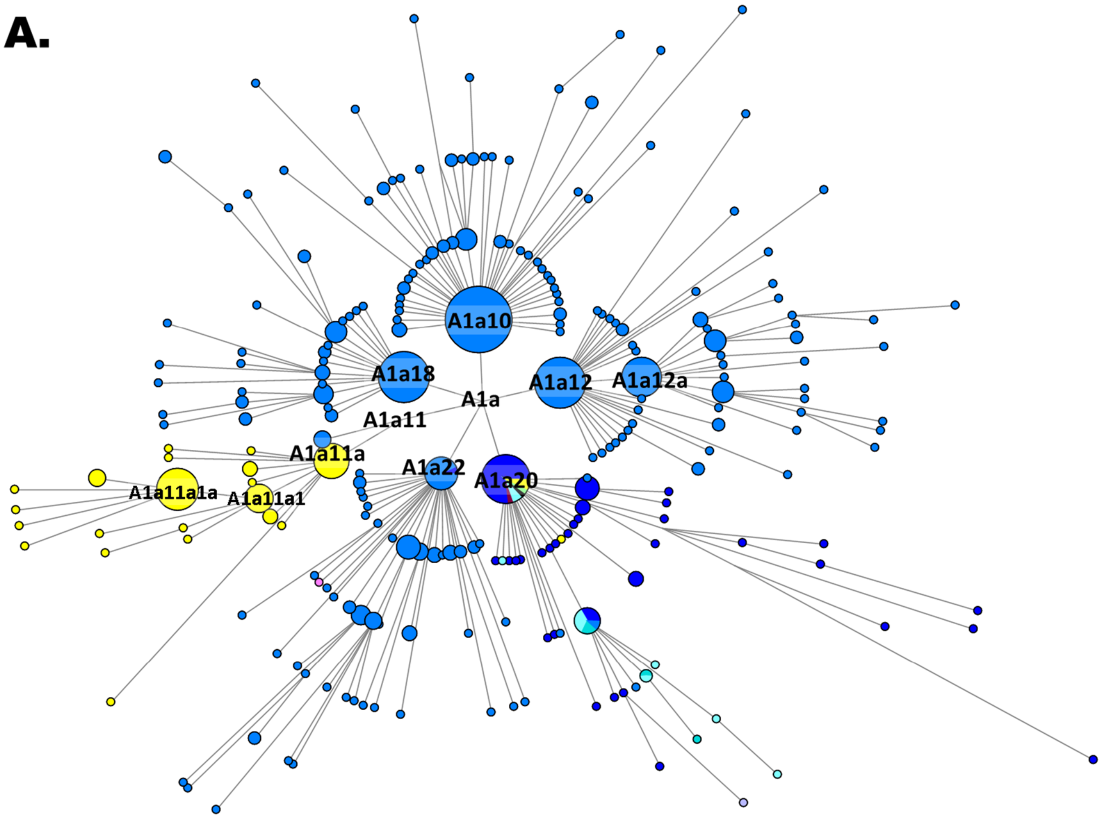

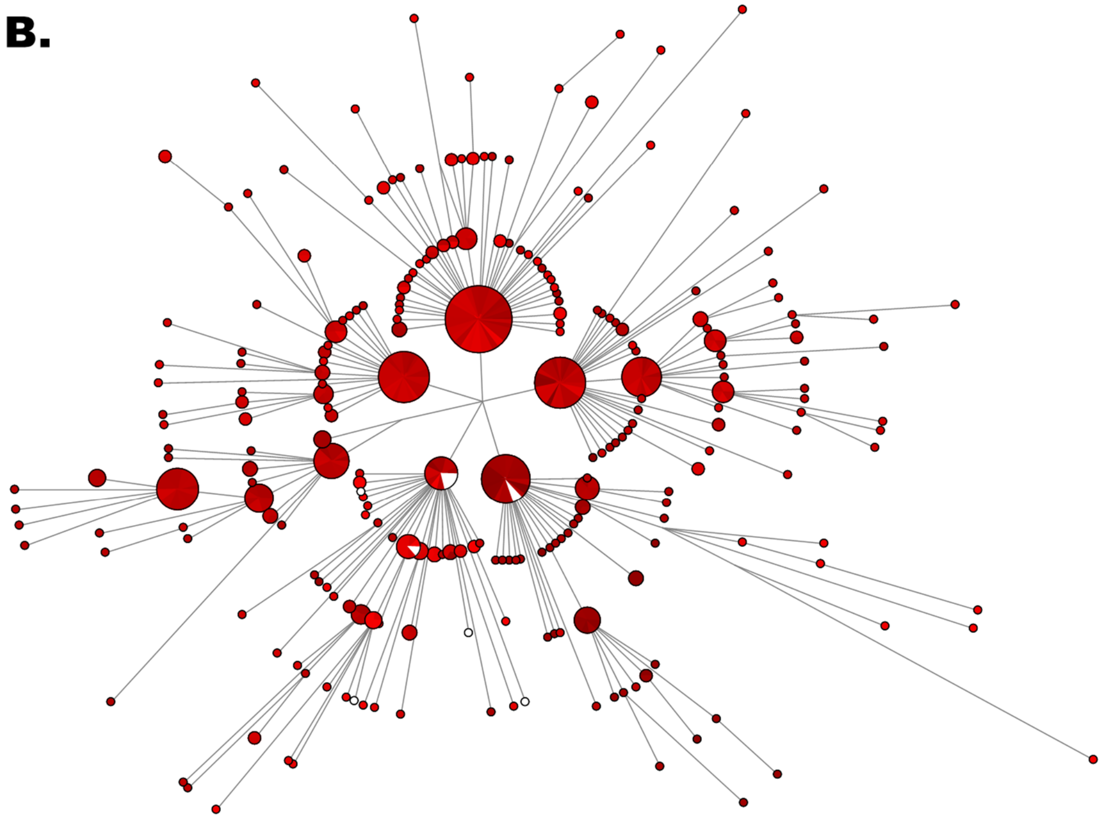

Figure S27. Reduced median network of subclades of A1a. Samples are colored according to geography (A) and time of collection (B) following the legend in Figure S4.

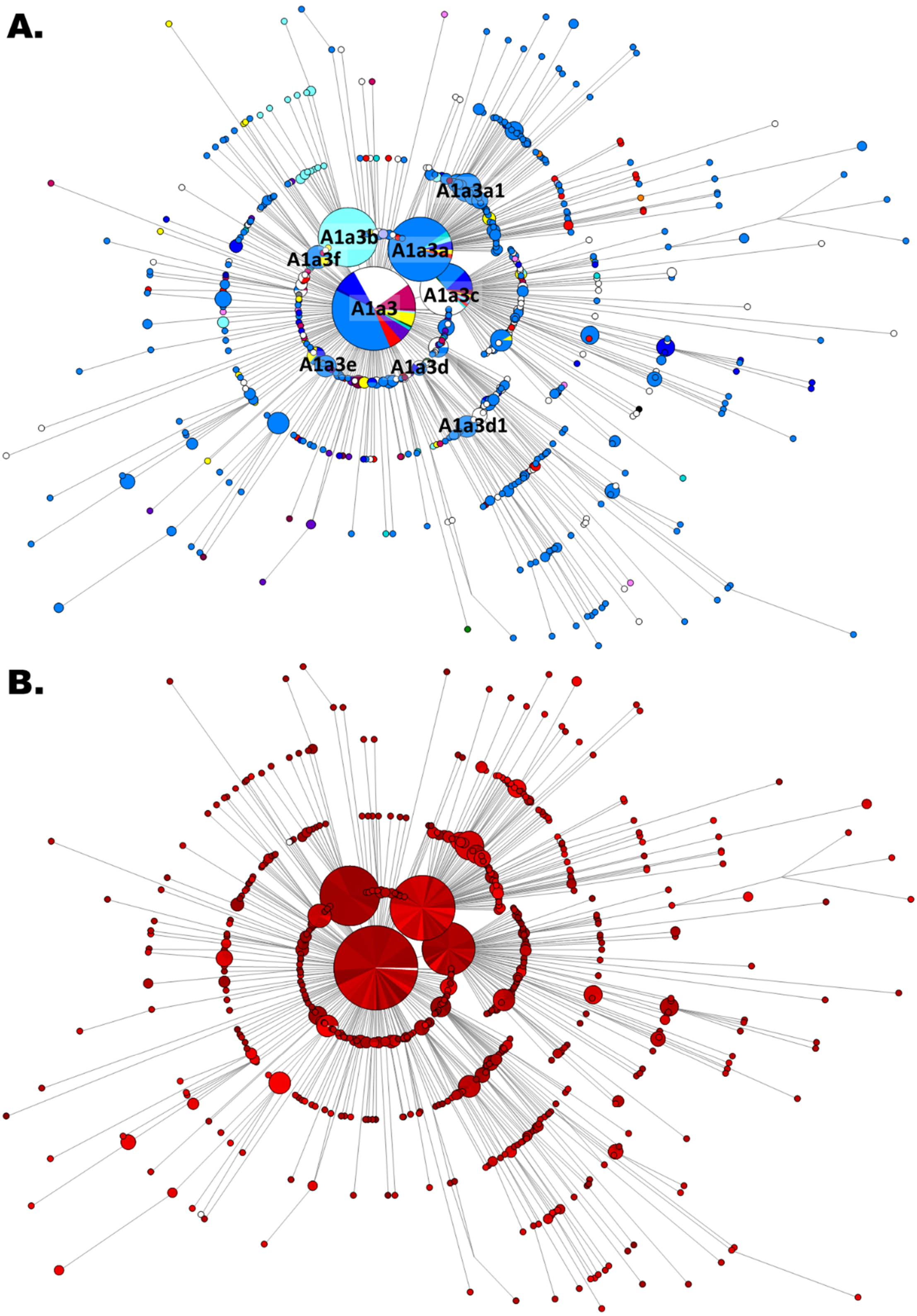

Figure S28. Reduced median network of clade A1a3. Samples are colored according to geography (A) and time of collection (B) following the legend in Figure S4.

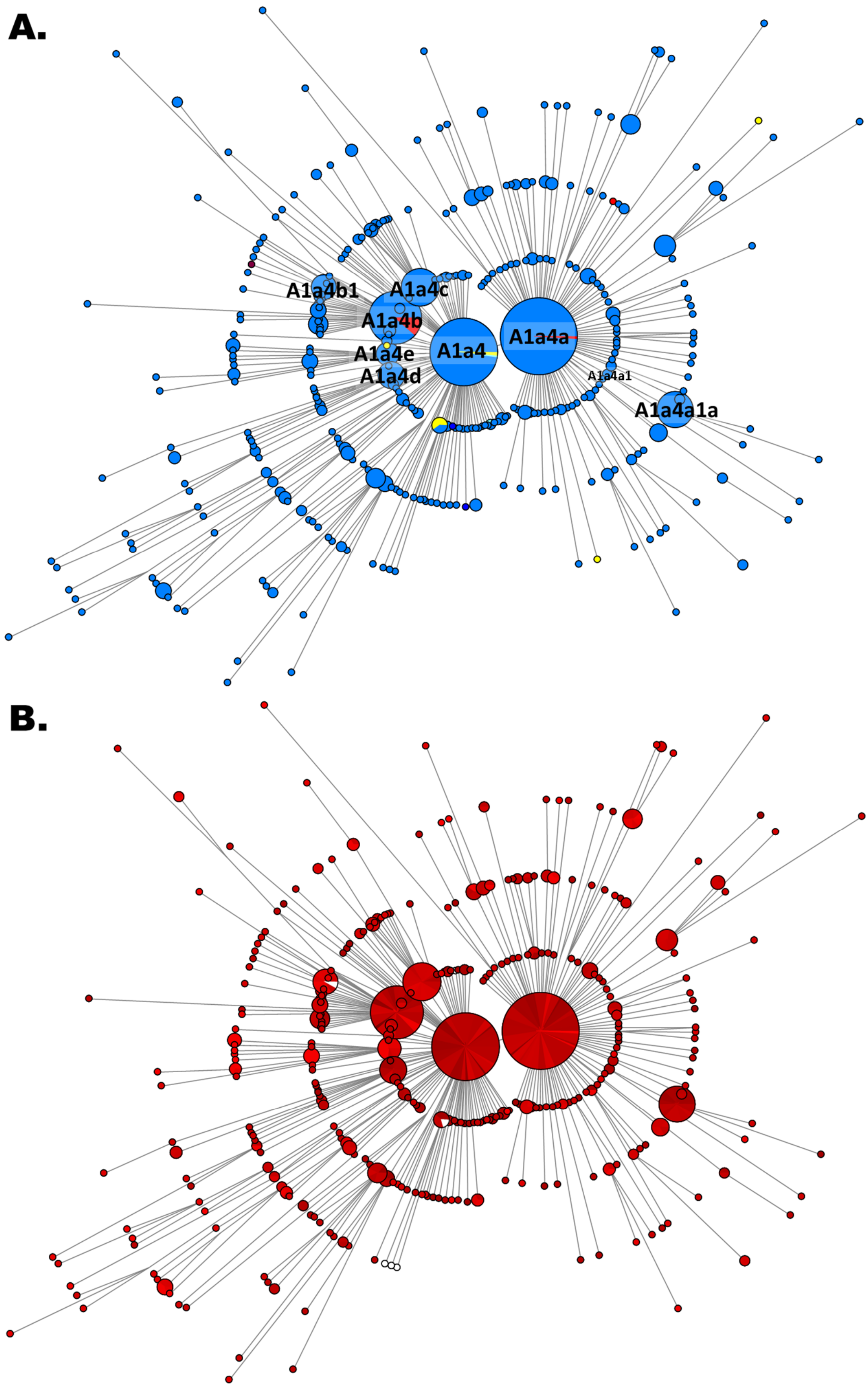

Figure S29. Reduced median network of clade A1a4. Samples are colored according to geography (A) and time of collection (B) following the legend in Figure S4.

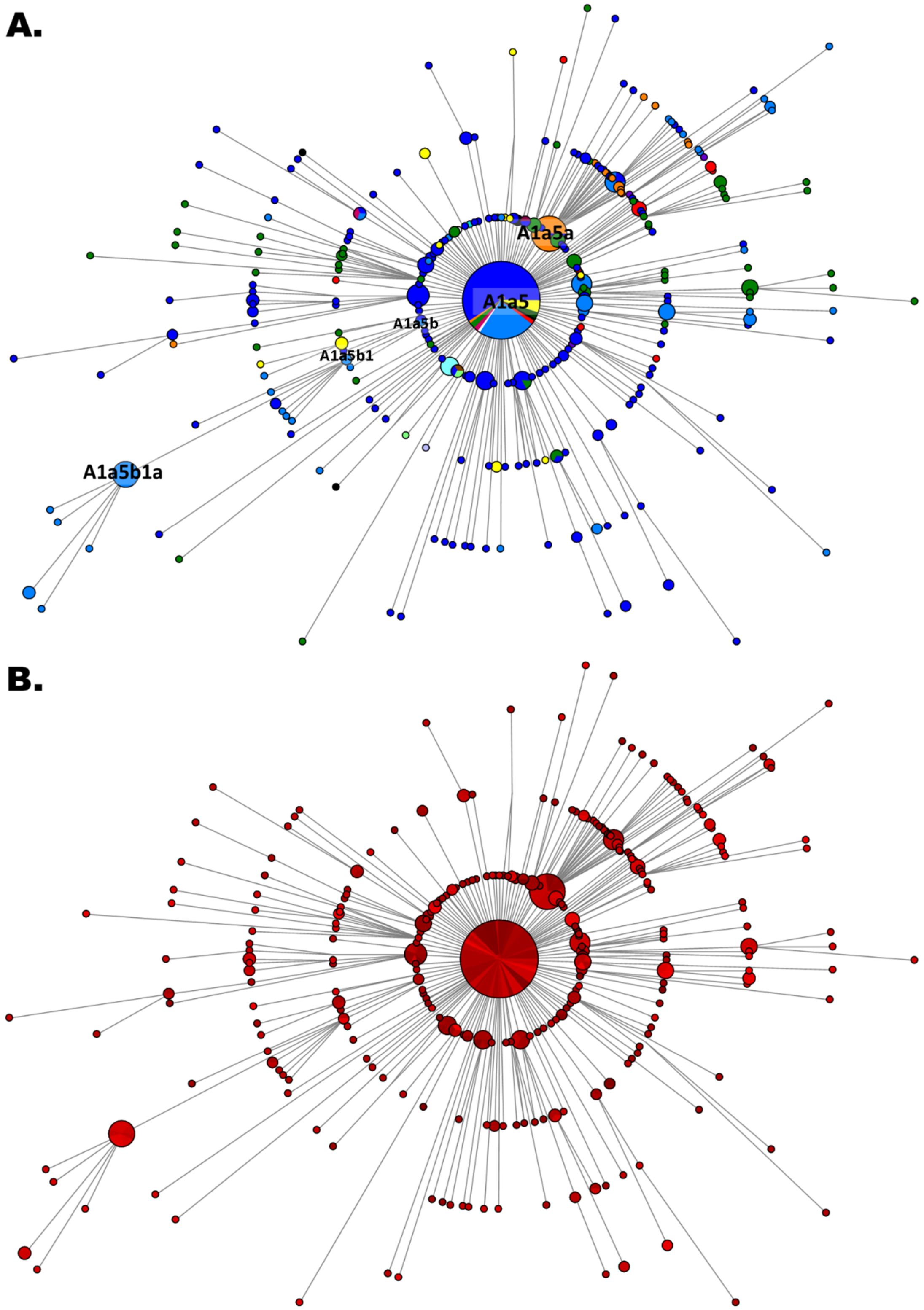

Figure S30. Reduced median network of clade A1a5. Samples are colored according to geography (A) and time of collection (B) following the legend in Figure S4.

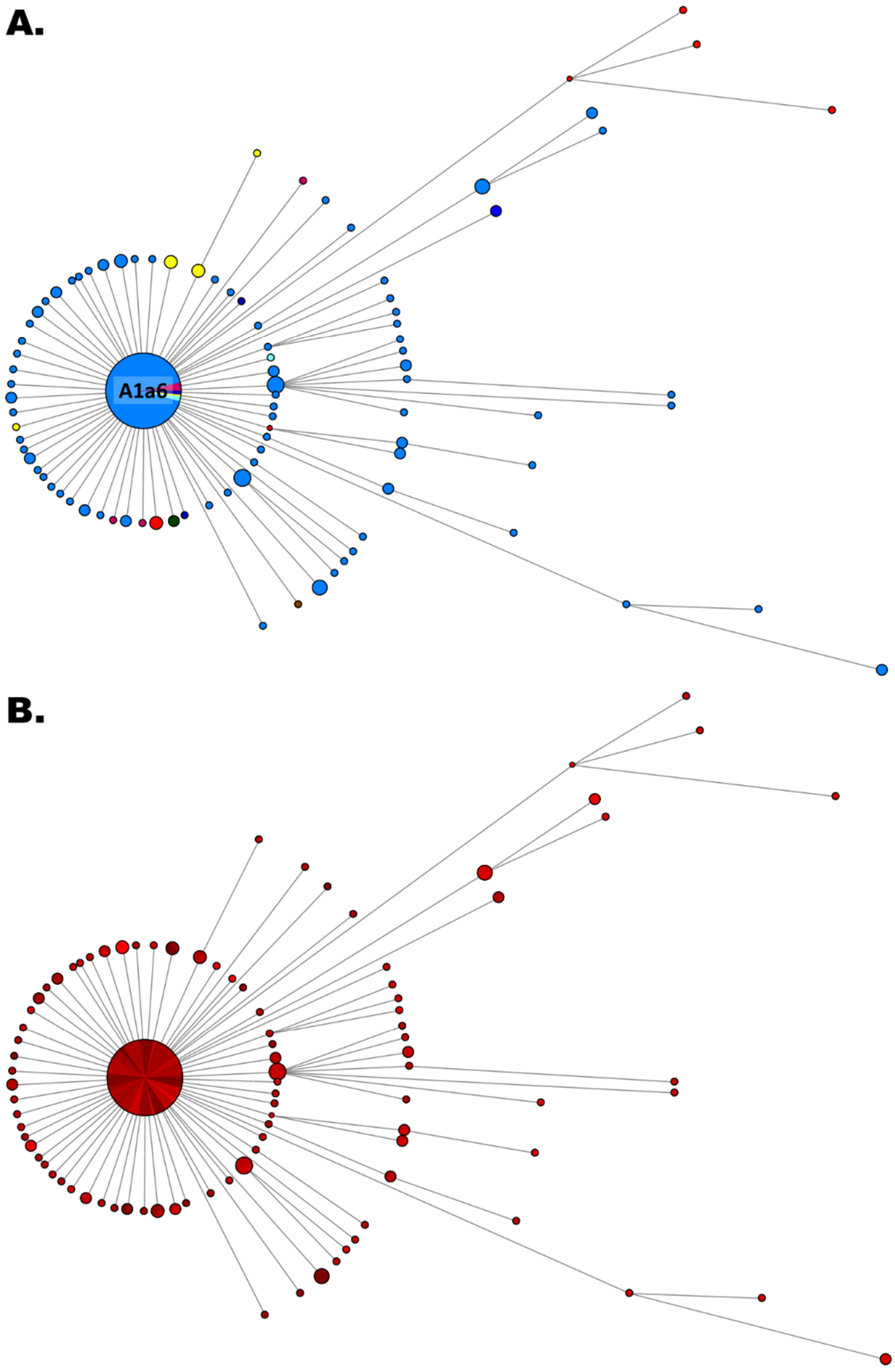

Figure S31. Reduced median network of clade A1a6. Samples are colored according to geography (A) and time of collection (B) following the legend in Figure S4.

A.

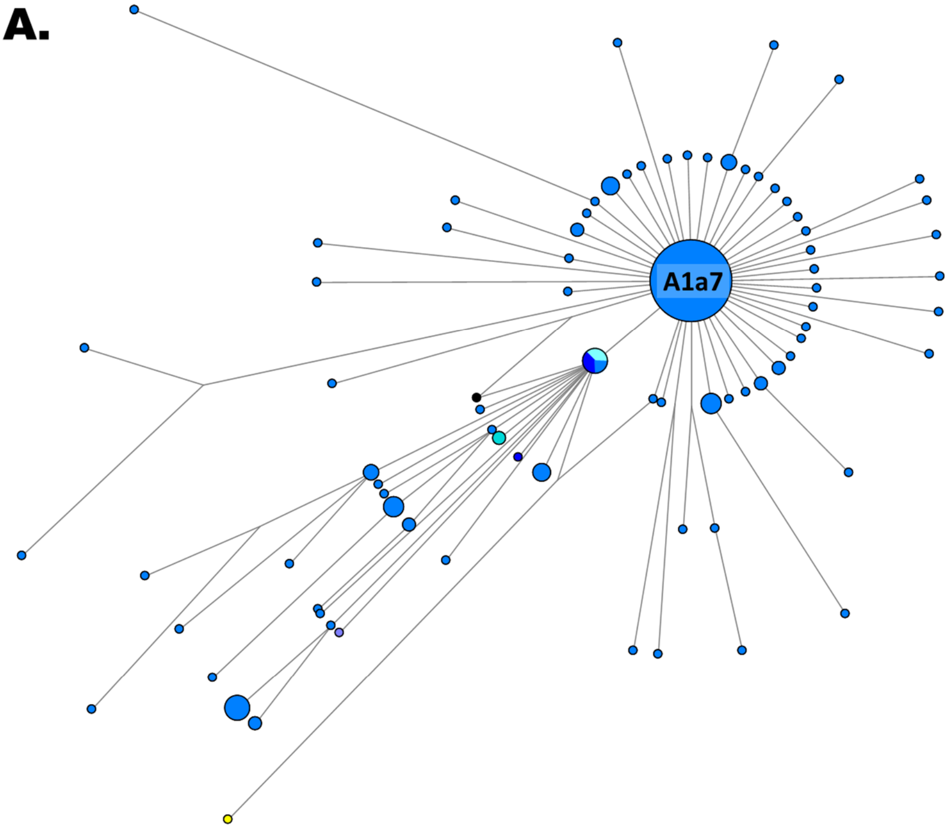

B.

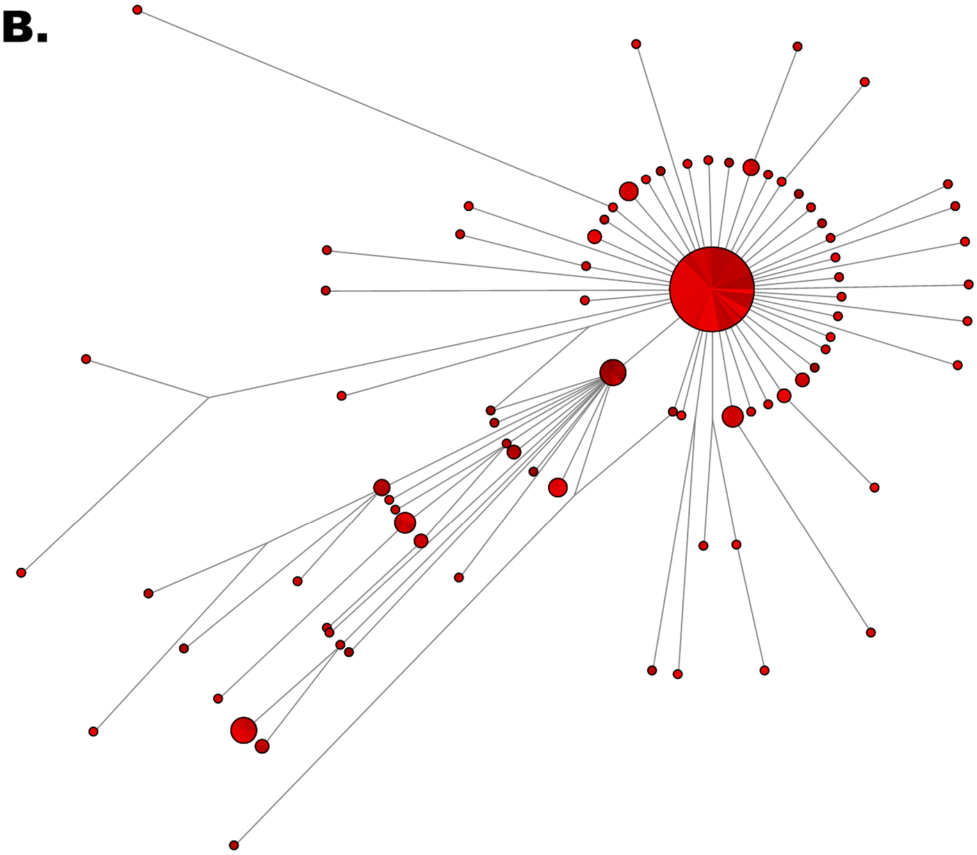

Figure S32. Reduced median network of clade A1a7. Samples are colored according to geography (A) and time of collection (B) following the legend in Figure S4.

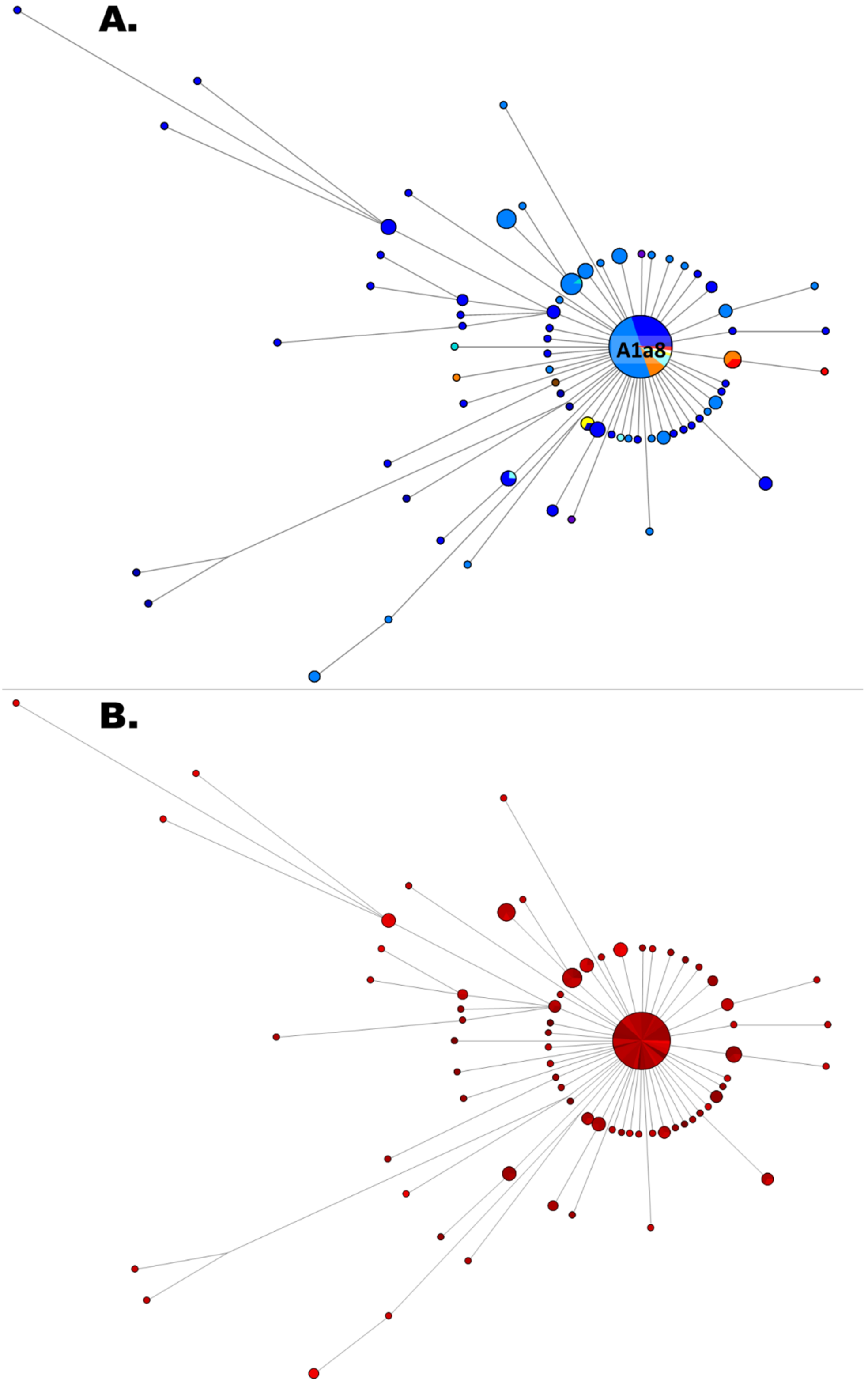

Figure S33. Reduced median network of clade A1a8. Samples are colored according to geography (A) and time of collection (B) following the legend in Figure S4.

A.

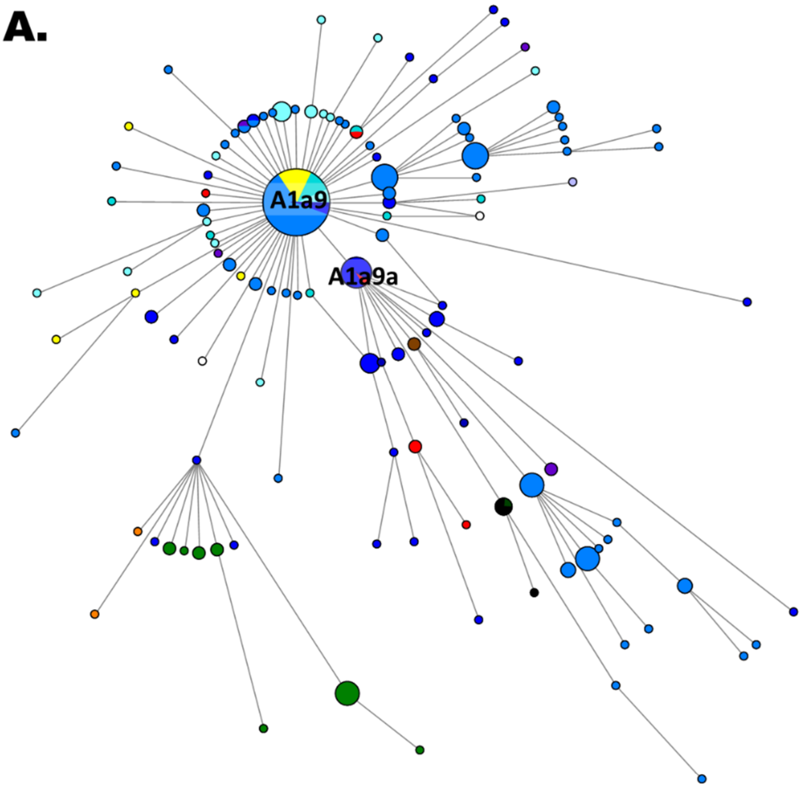

B.

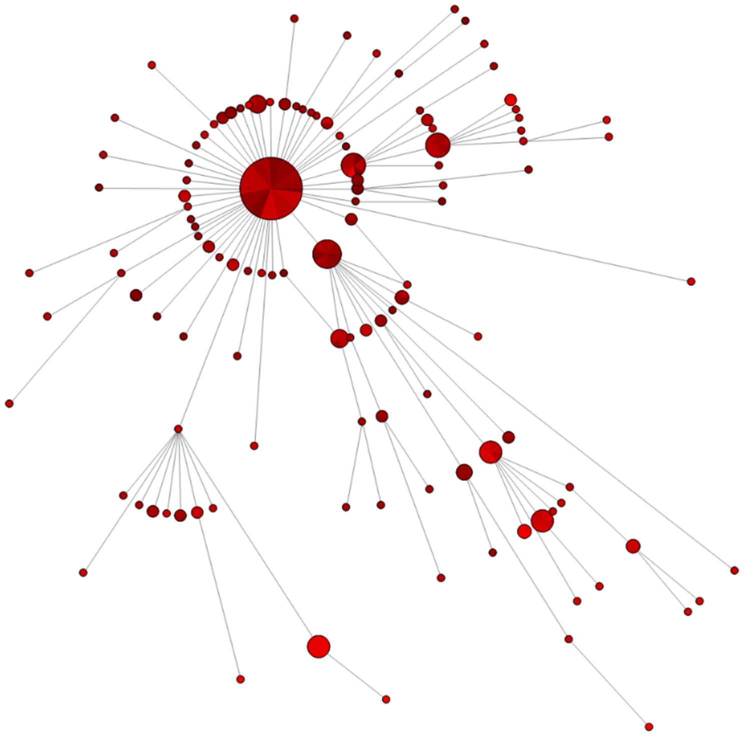

Figure S34. Reduced median network of clade A1a9. Samples are colored according to geography (A) and time of collection (B) following the legend in Figure S4.

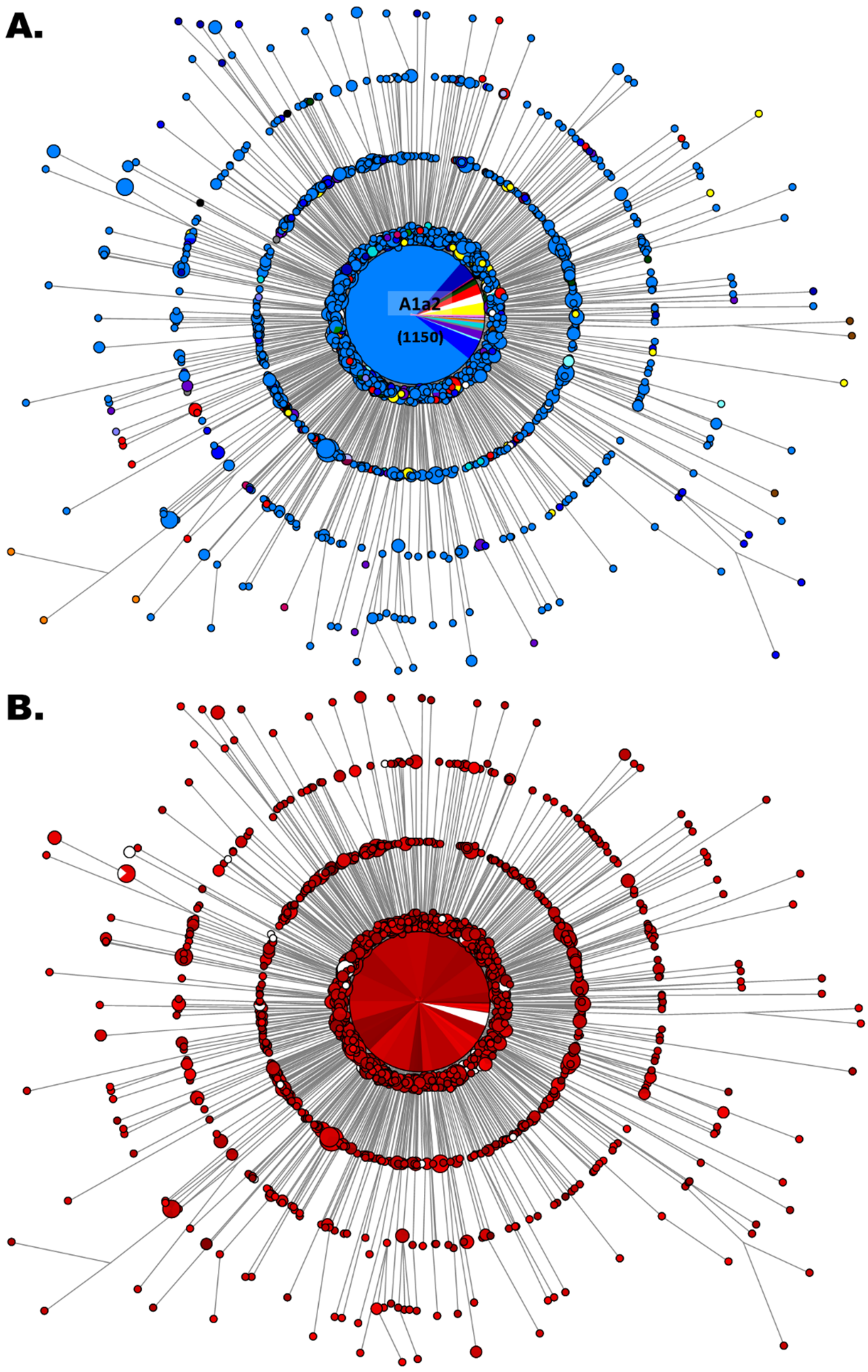

Figure S35. Reduced median network of paragroup A1a2. The value between brackets refers to the total number of samples in the largest haplotype that is not proportional to the sample size. Samples are colored according to geography (A) and time of collection (B) following the legend in Figure S4.

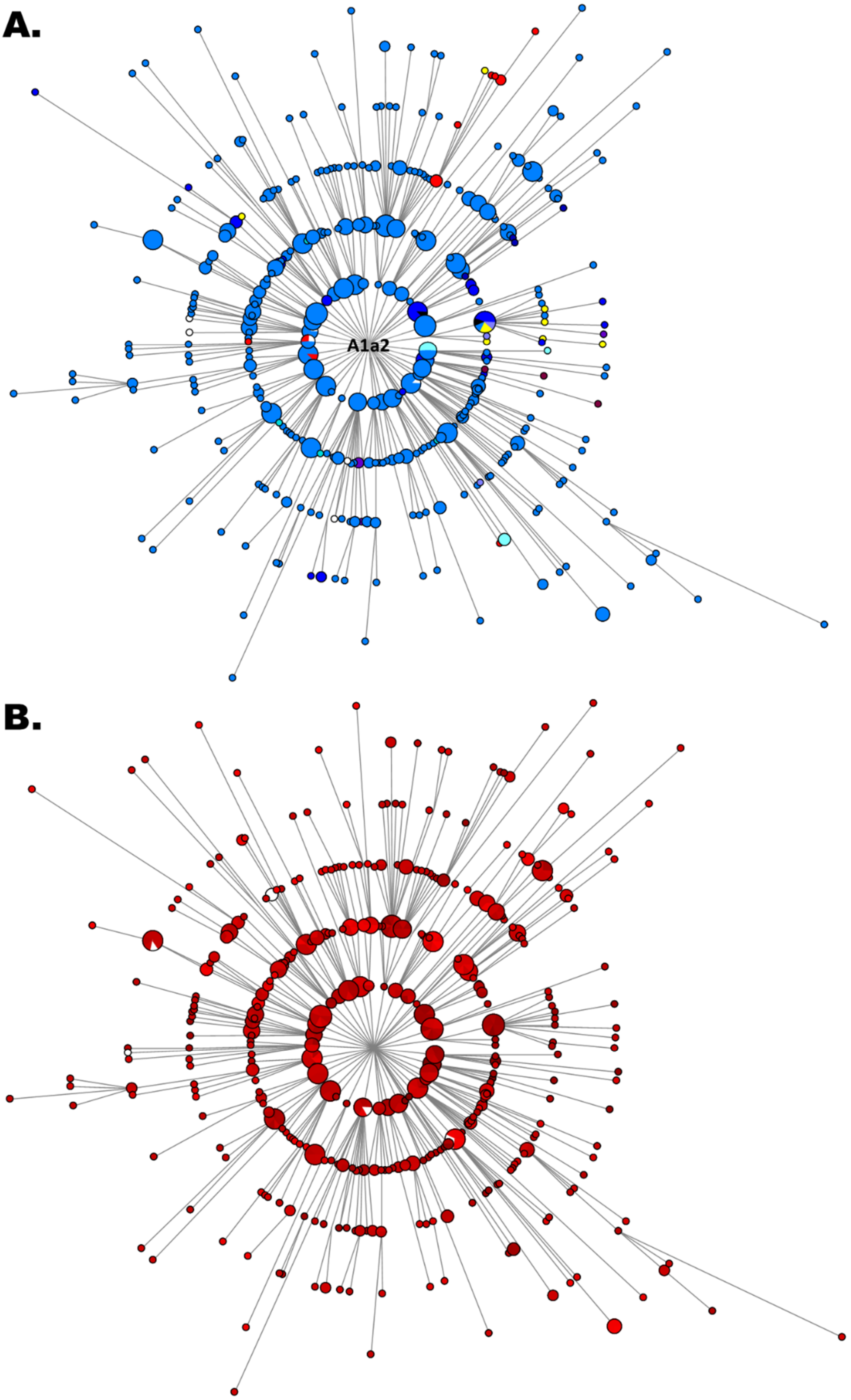

Figure S36. Reduced median network of minor subclades of A1a2. Samples are colored according to geography (A) and time of collection (B) following the legend in Figure S4.

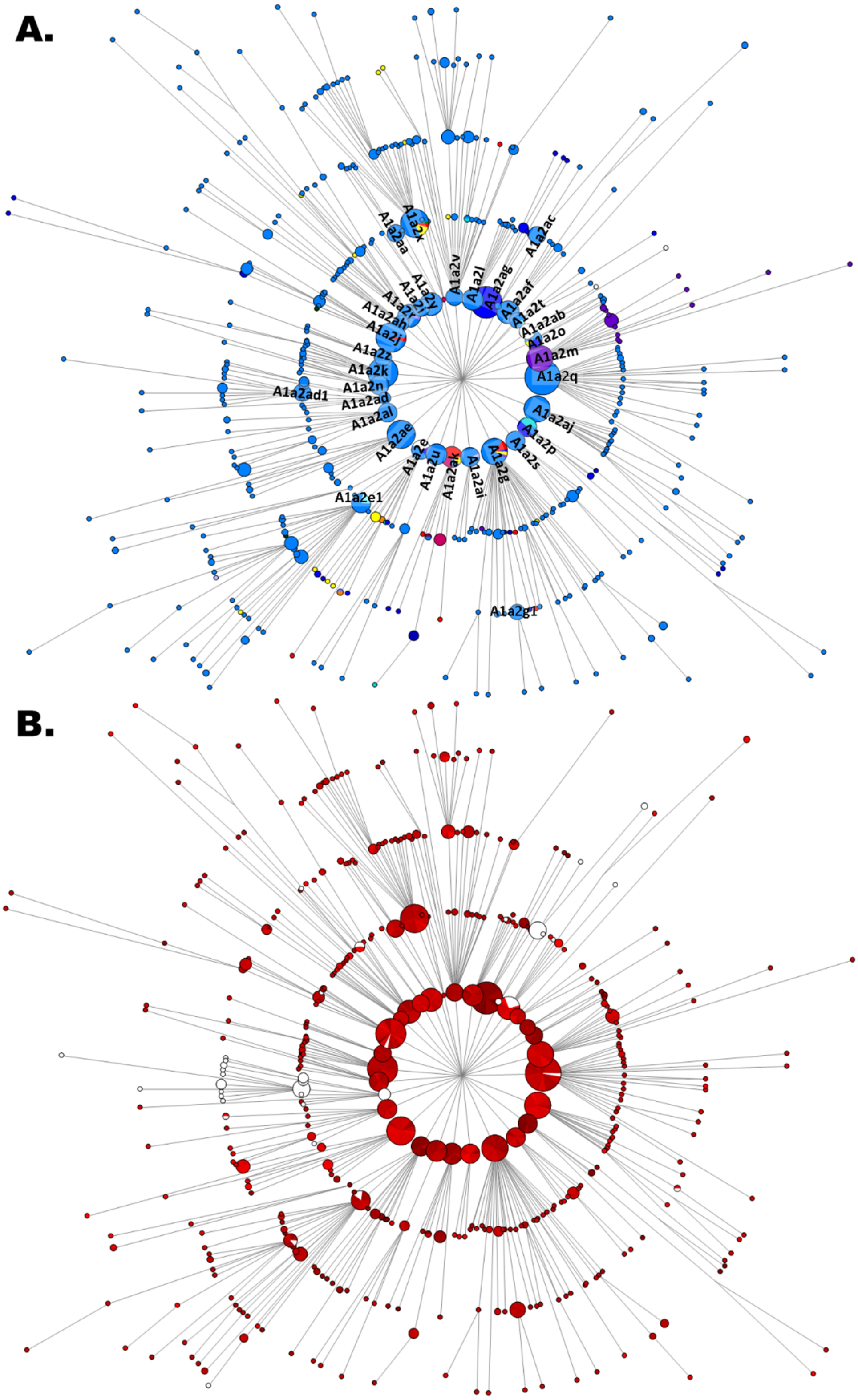

Figure S37. Reduced median network of subclades of A1a2. Samples are colored according to geography (A) and time of collection (B) following the legend in Figure S4.

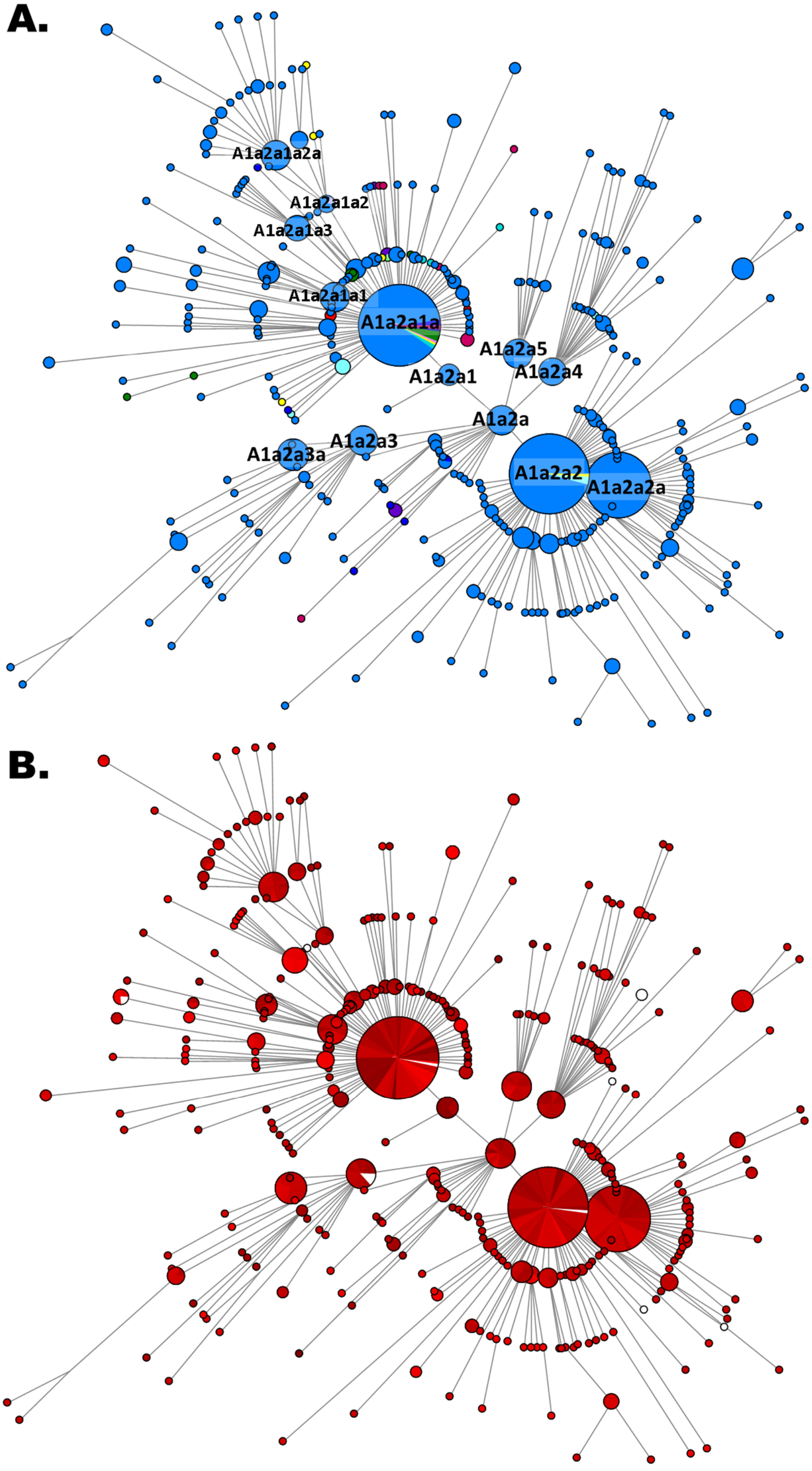

Figure S38. Reduced median network of clades of A1a2a. Samples are colored according to geography (A) and time of collection (B) following the legend in Figure S4.

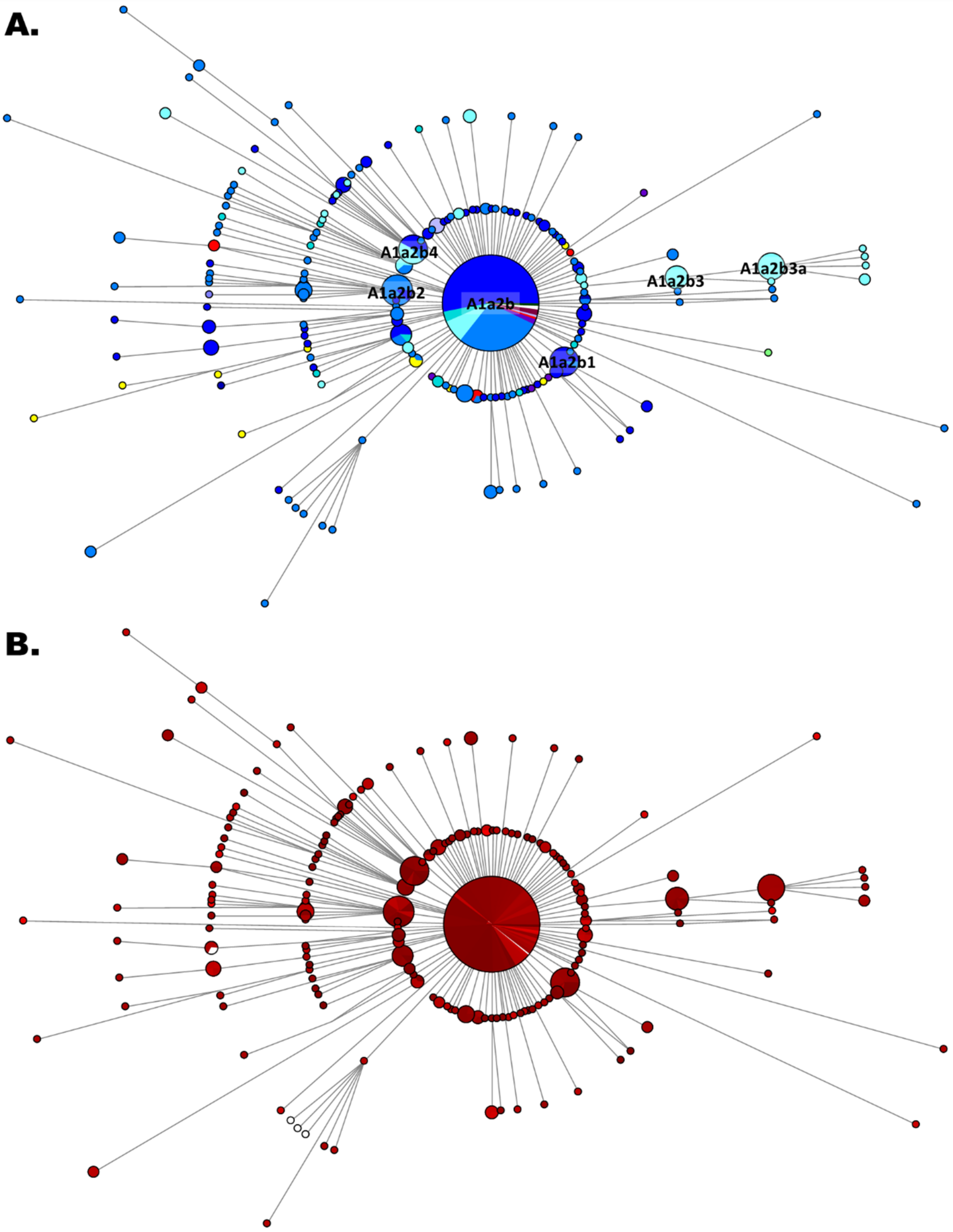

Figure S39. Reduced median network of clades of A1a2b. Samples are colored according to geography (A) and time of collection (B) following the legend in Figure S4.

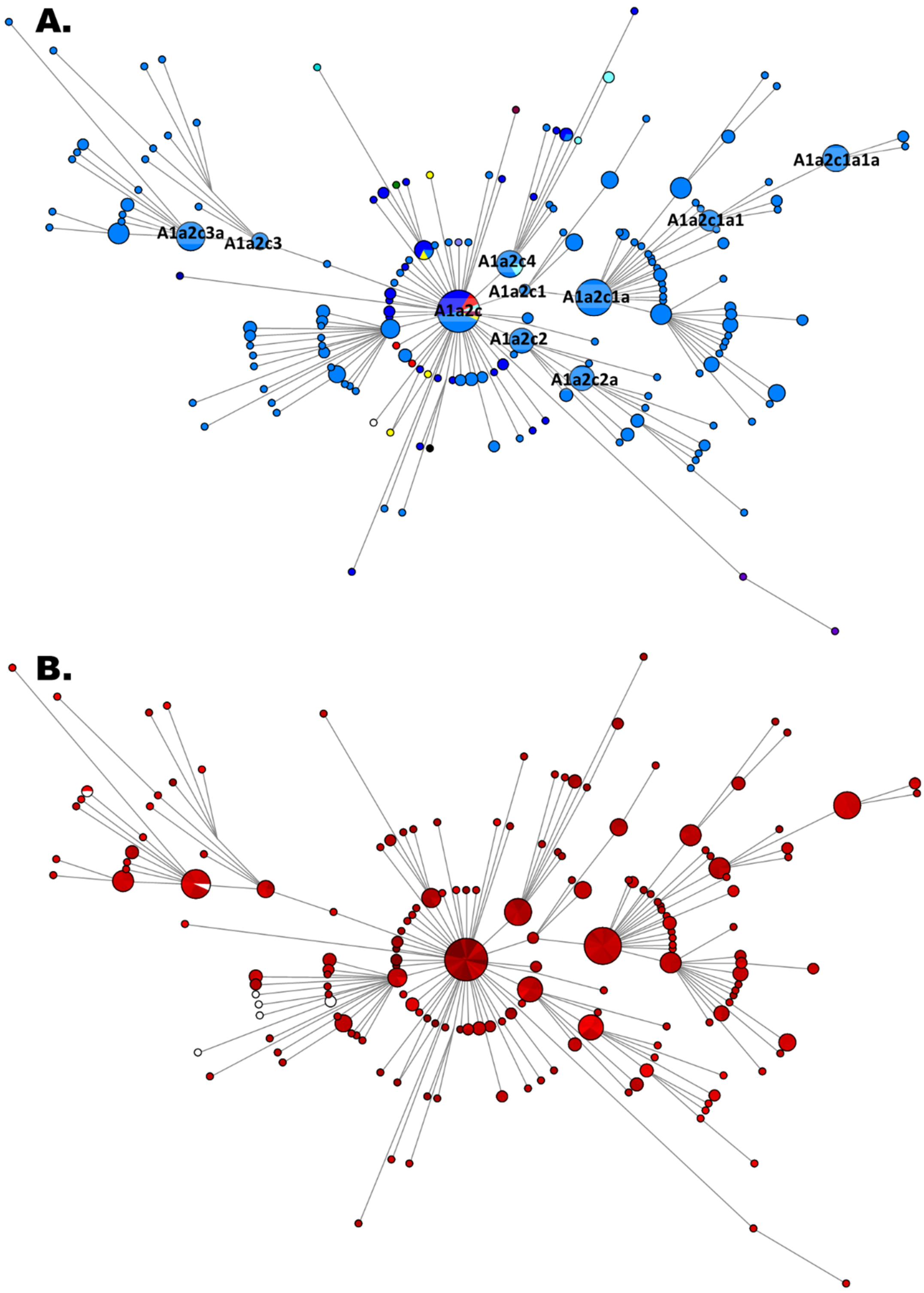

Figure S40. Reduced median network of clades of A1a2c. Samples are colored according to geography (A) and time of collection (B) following the legend in Figure S4.

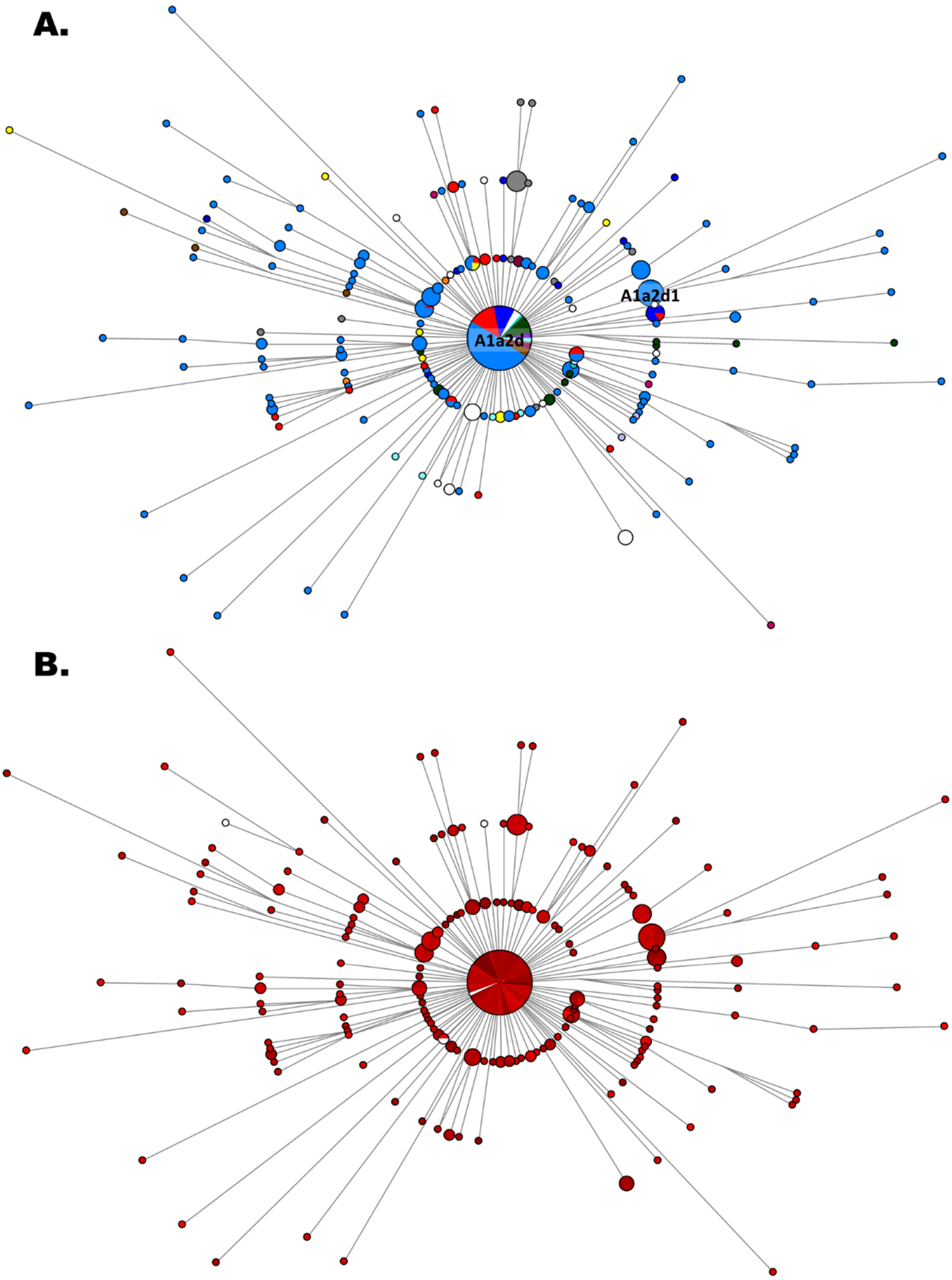

Figure S41. Reduced median network of clades of A1a2d. Samples are colored according to geography (A) and time of collection (B) following the legend in Figure S4.

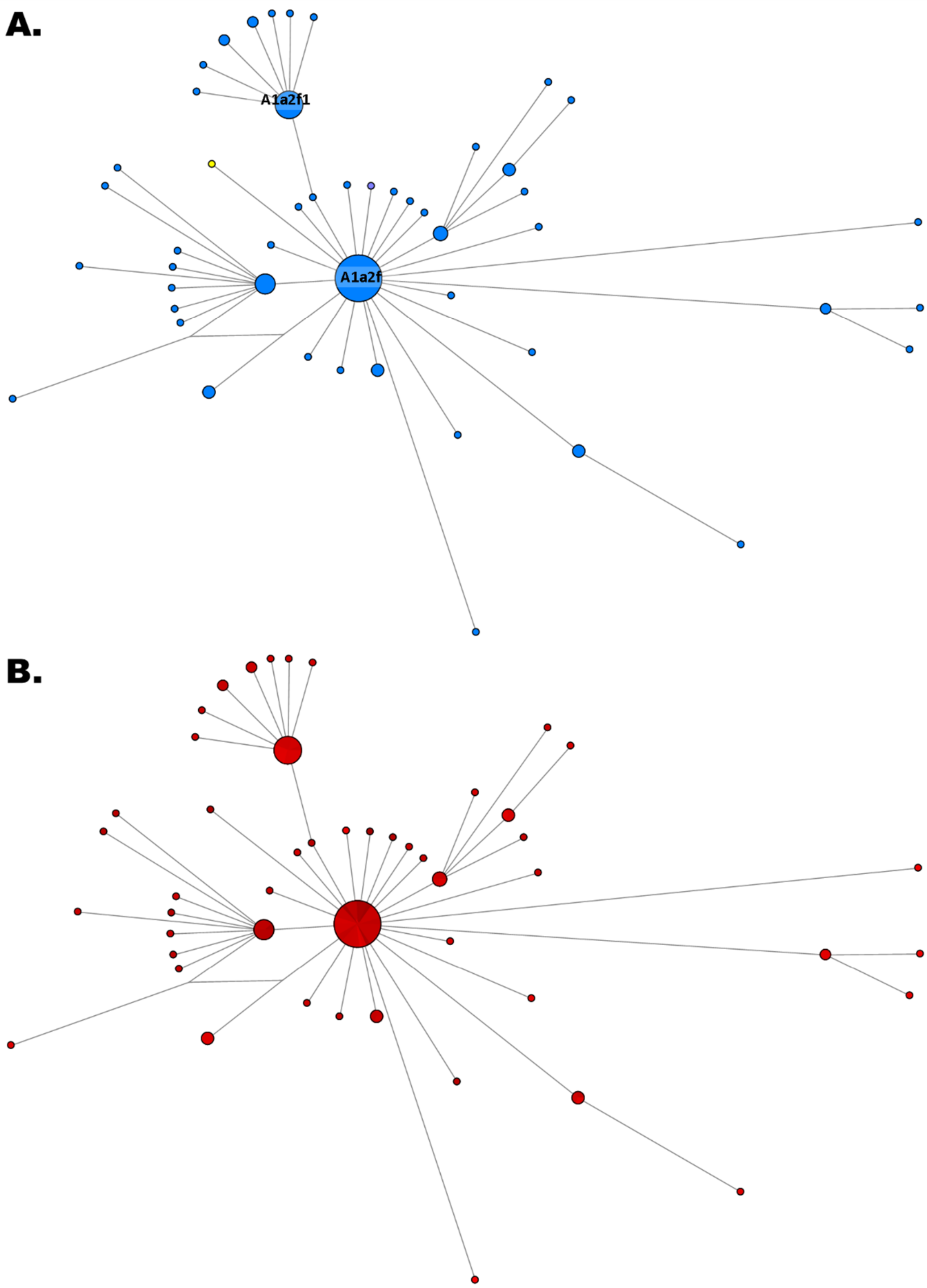

Figure S42. Reduced median network of clades of A1a2f. Samples are colored according to geography (A) and time of collection (B) following the legend in Figure S4.

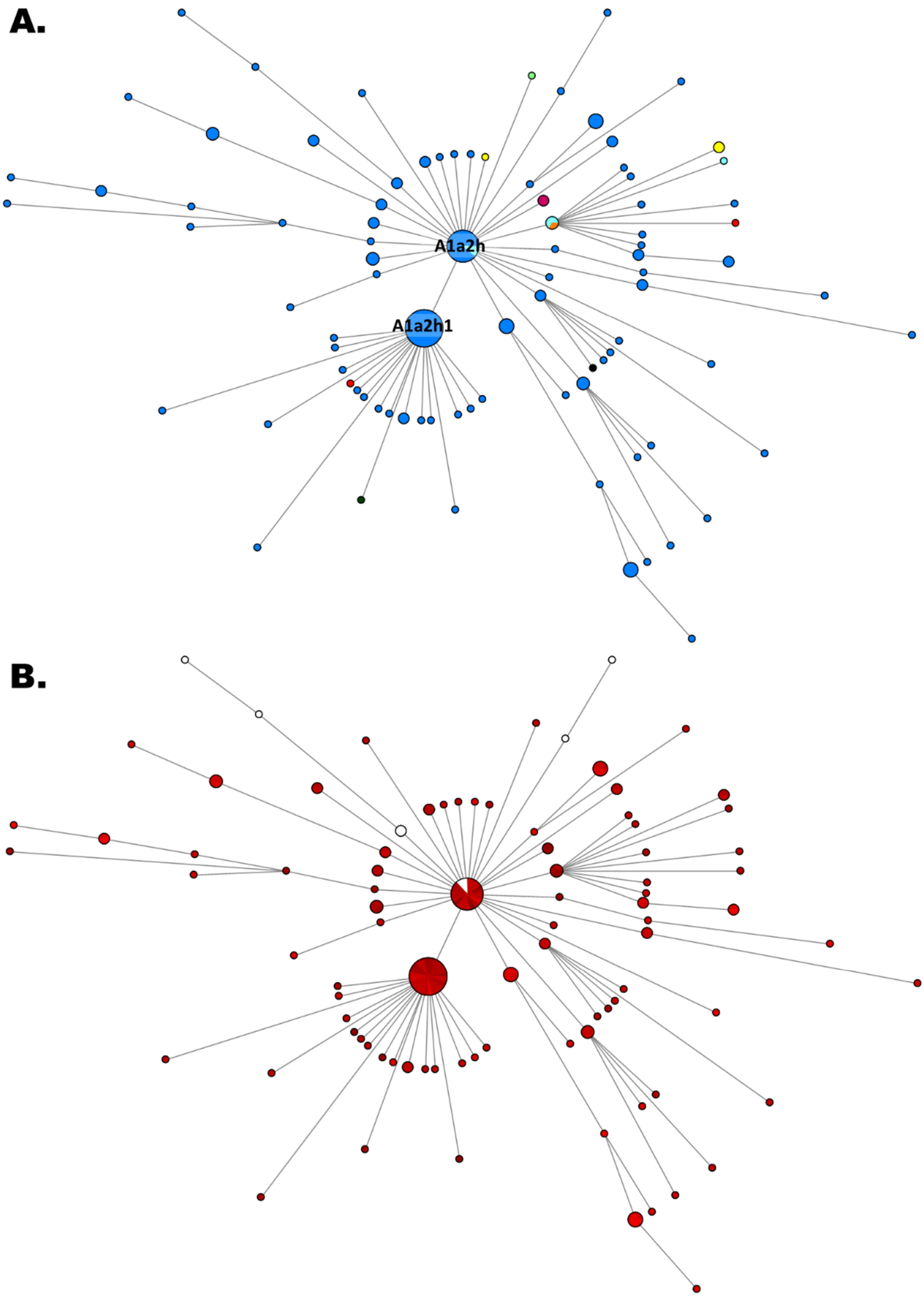

Figure S43. Reduced median network of clades of A1a2h. Samples are colored according to geography (A) and time of collection (B) following the legend in Figure S4.

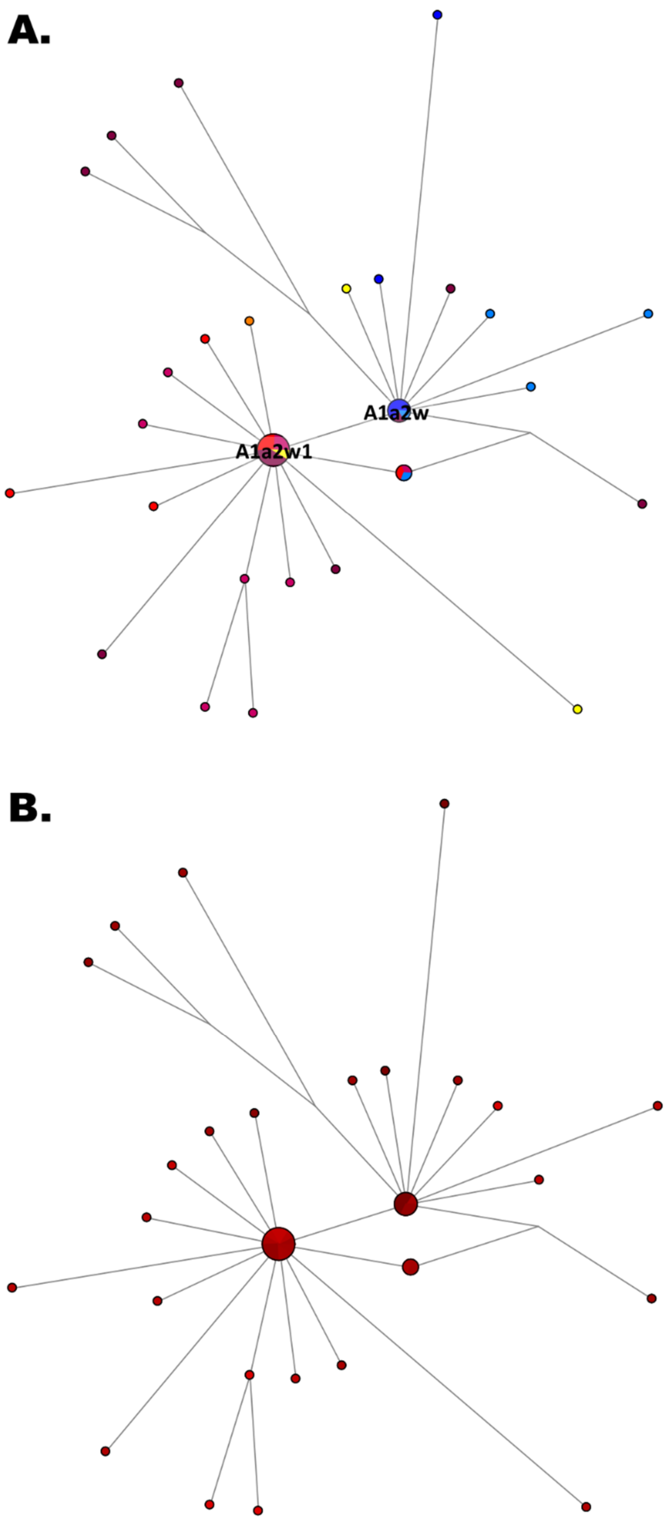

Figure S44. Reduced median network of clades of A1a2w. Samples are colored according to geography (A) and time of collection (B) following the legend in Figure S4.

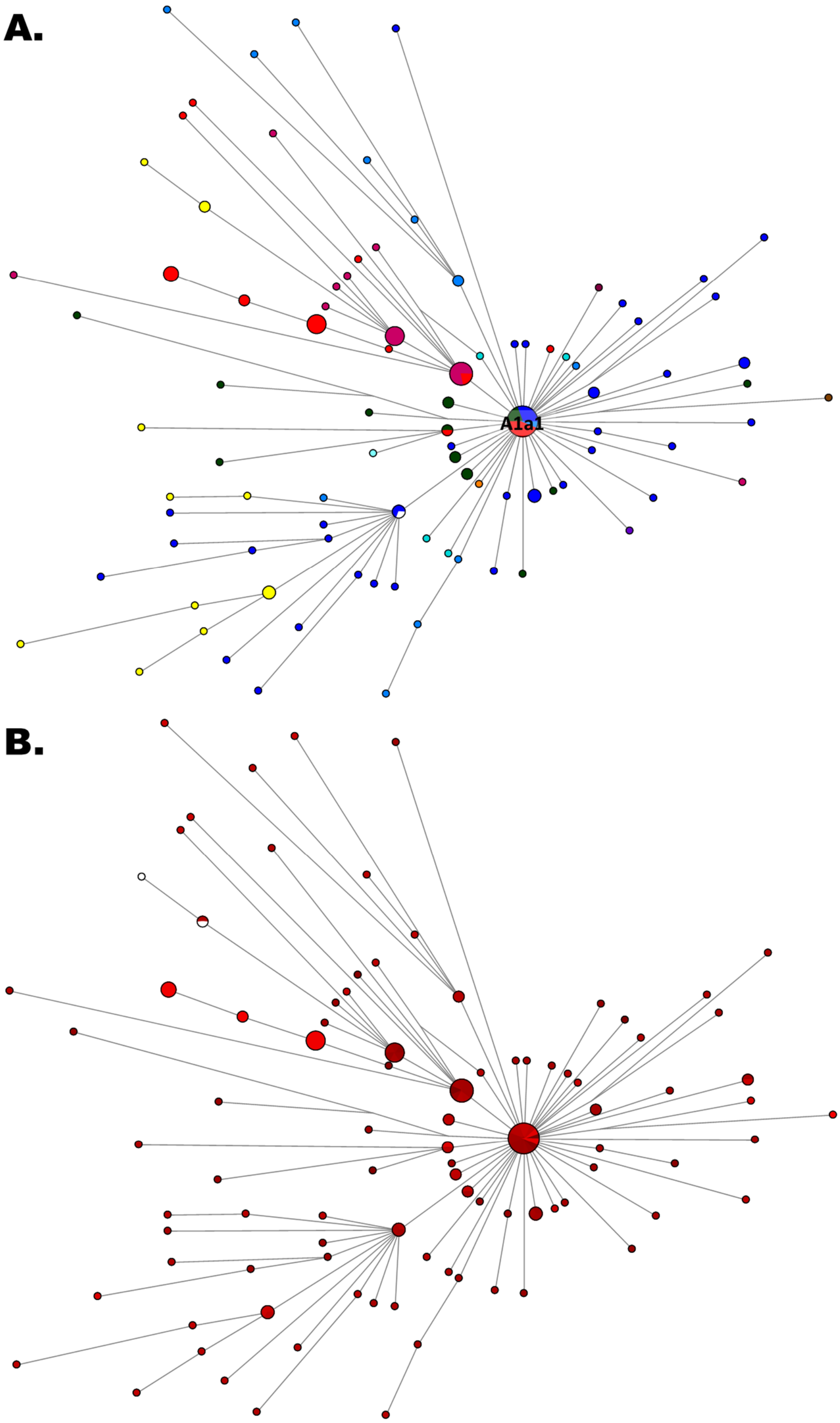

Figure S45. Reduced median network of clades of paragroup A1a1. Samples are colored according to geography (A) and time of collection (B) following the legend in Figure S4.

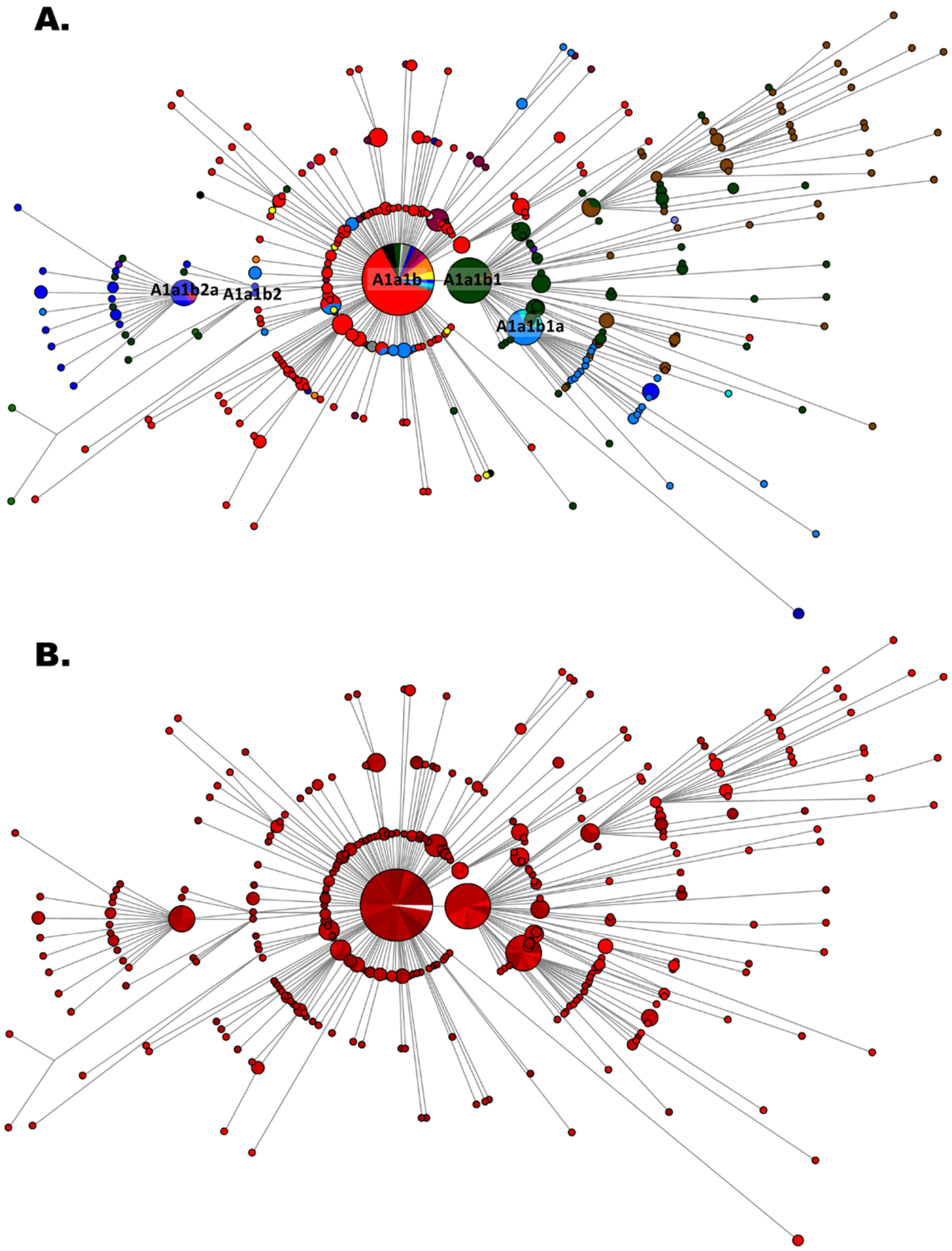

Figure S46. Reduced median network of clades of clade A1a1b. Samples are colored according to geography (A) and time of collection (B) following the legend in Figure S4.

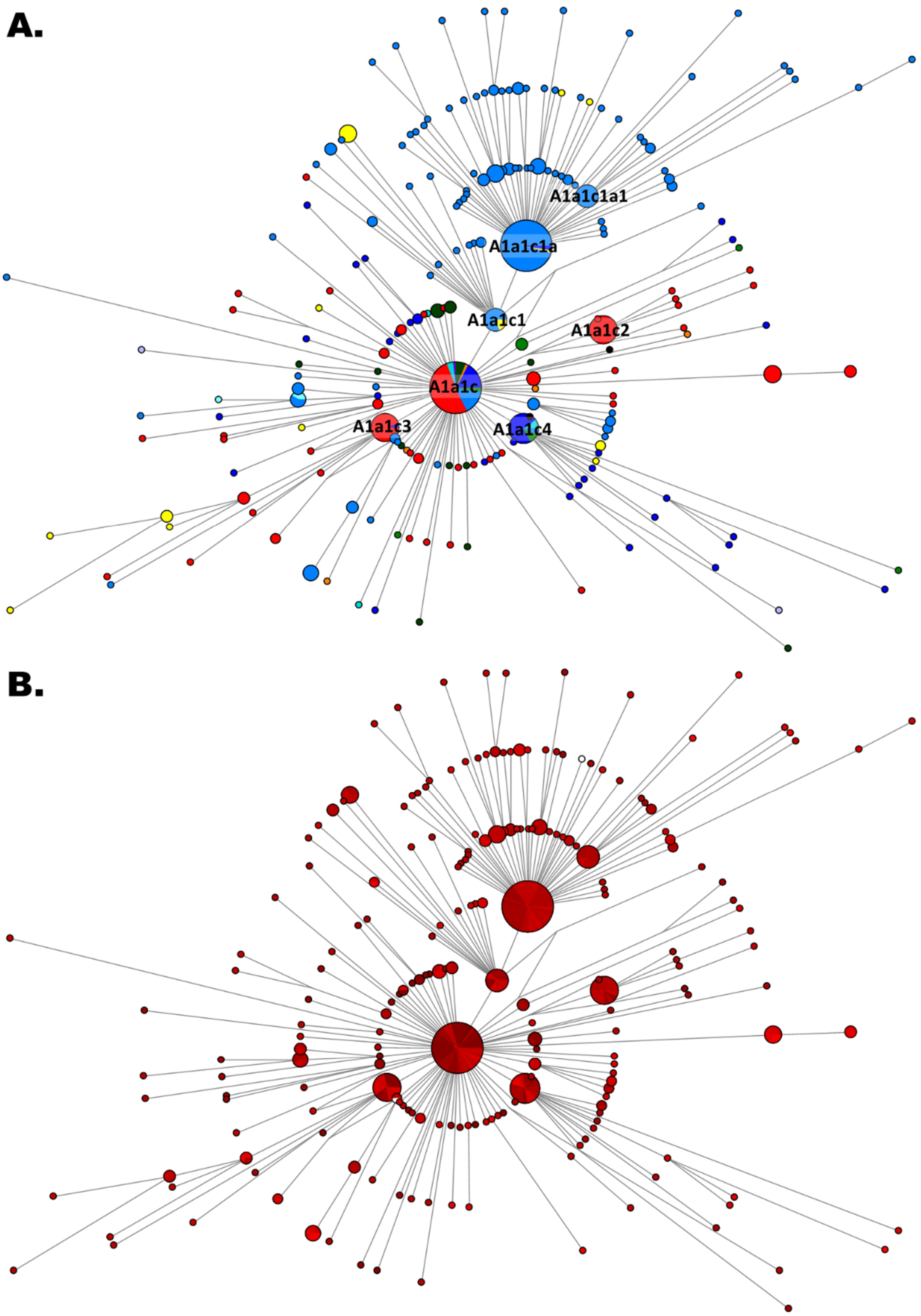

Figure S47. Reduced median network of clades of clade A1a1c. Samples are colored according to geography (A) and time of collection (B) following the legend in Figure S4.

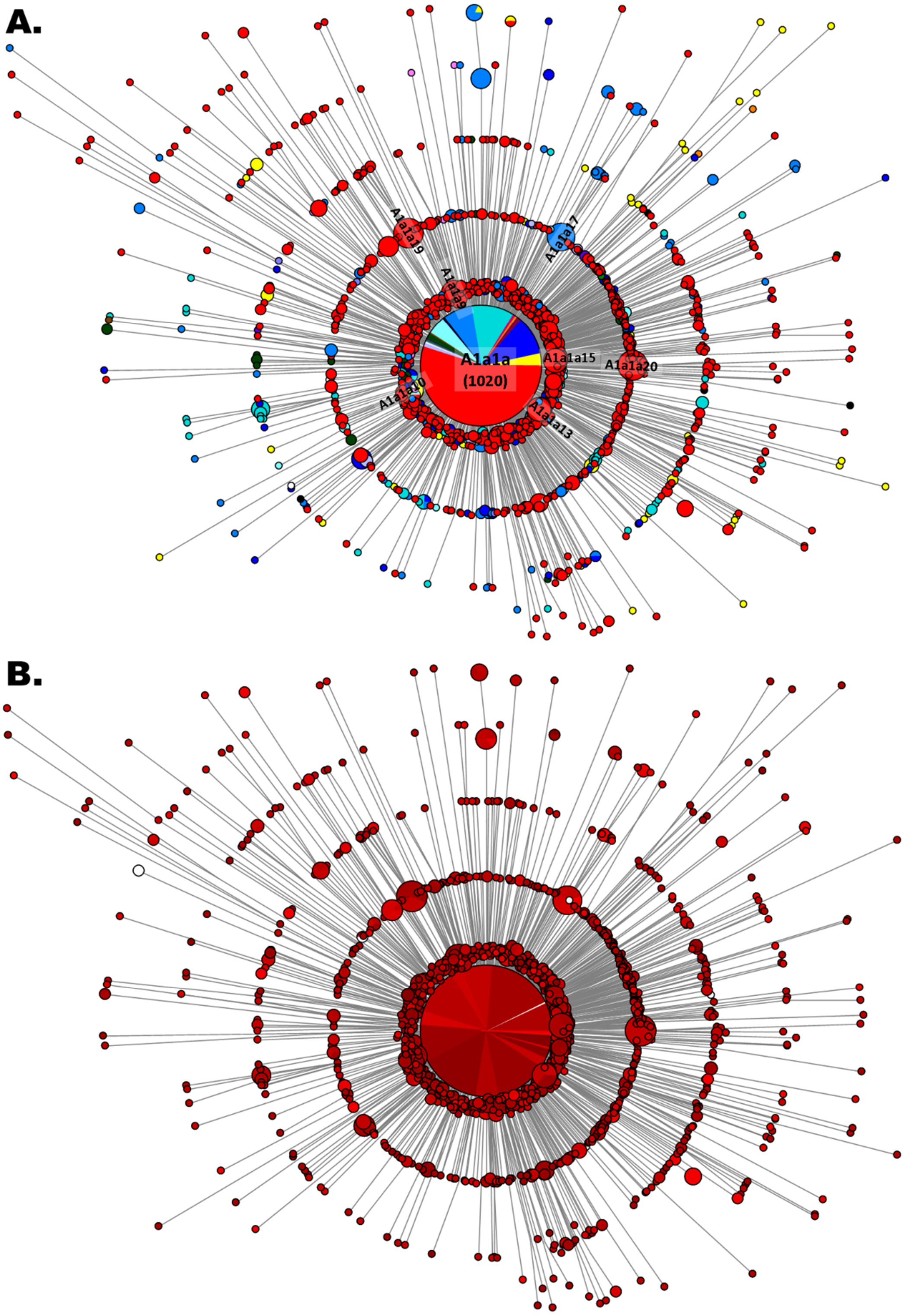

Figure S48. Reduced median network of paragroup A1a1a. The value between brackets refers to the total number of samples in the largest haplotype that is not proportional to the sample size. Samples are colored according to geography (A) and time of collection (B) following the legend in Figure S4.

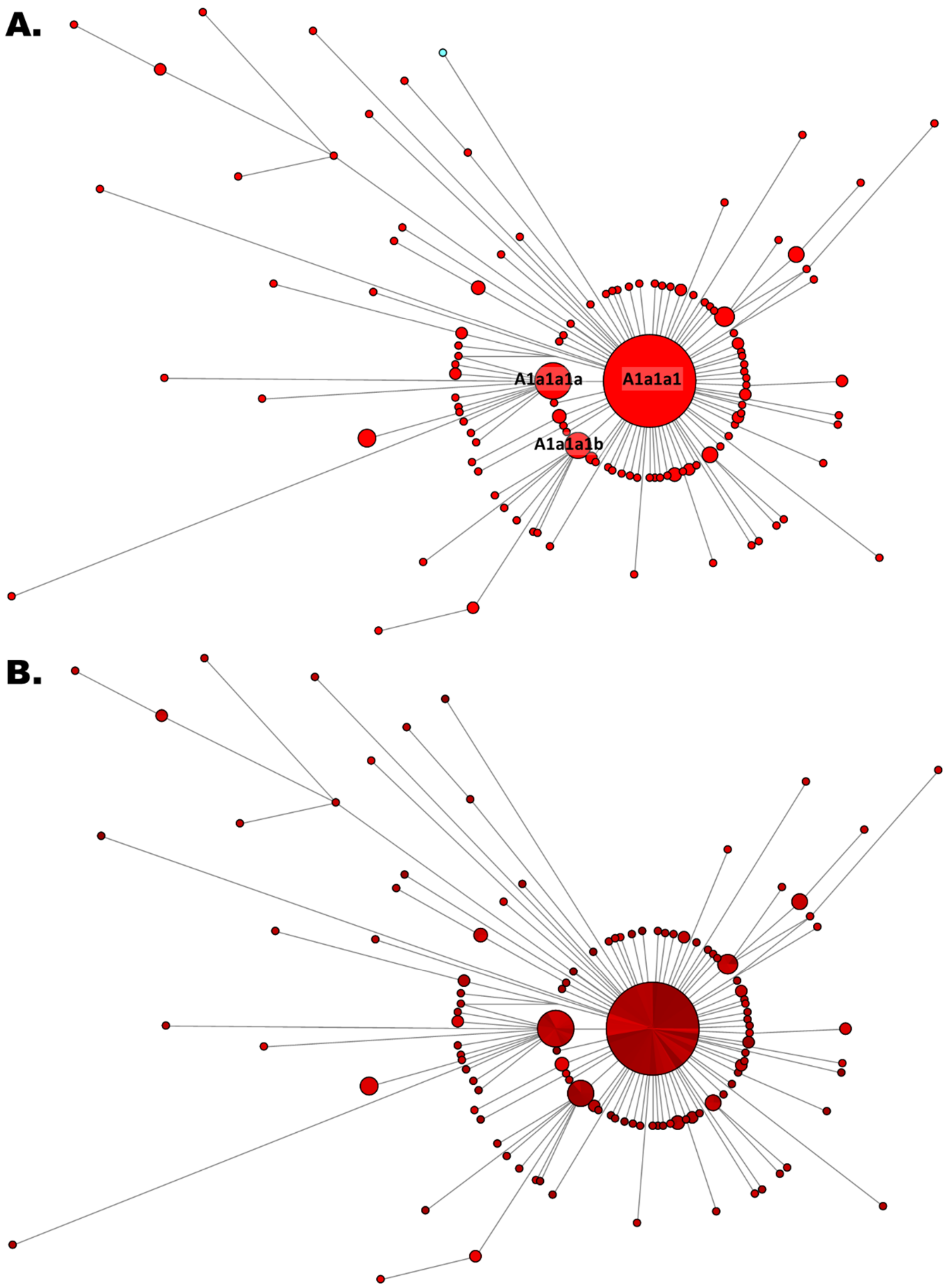

Figure S49. Reduced median network of clades of clade A1a1a1. Samples are colored according to geography (A) and time of collection (B) following the legend in Figure S4.

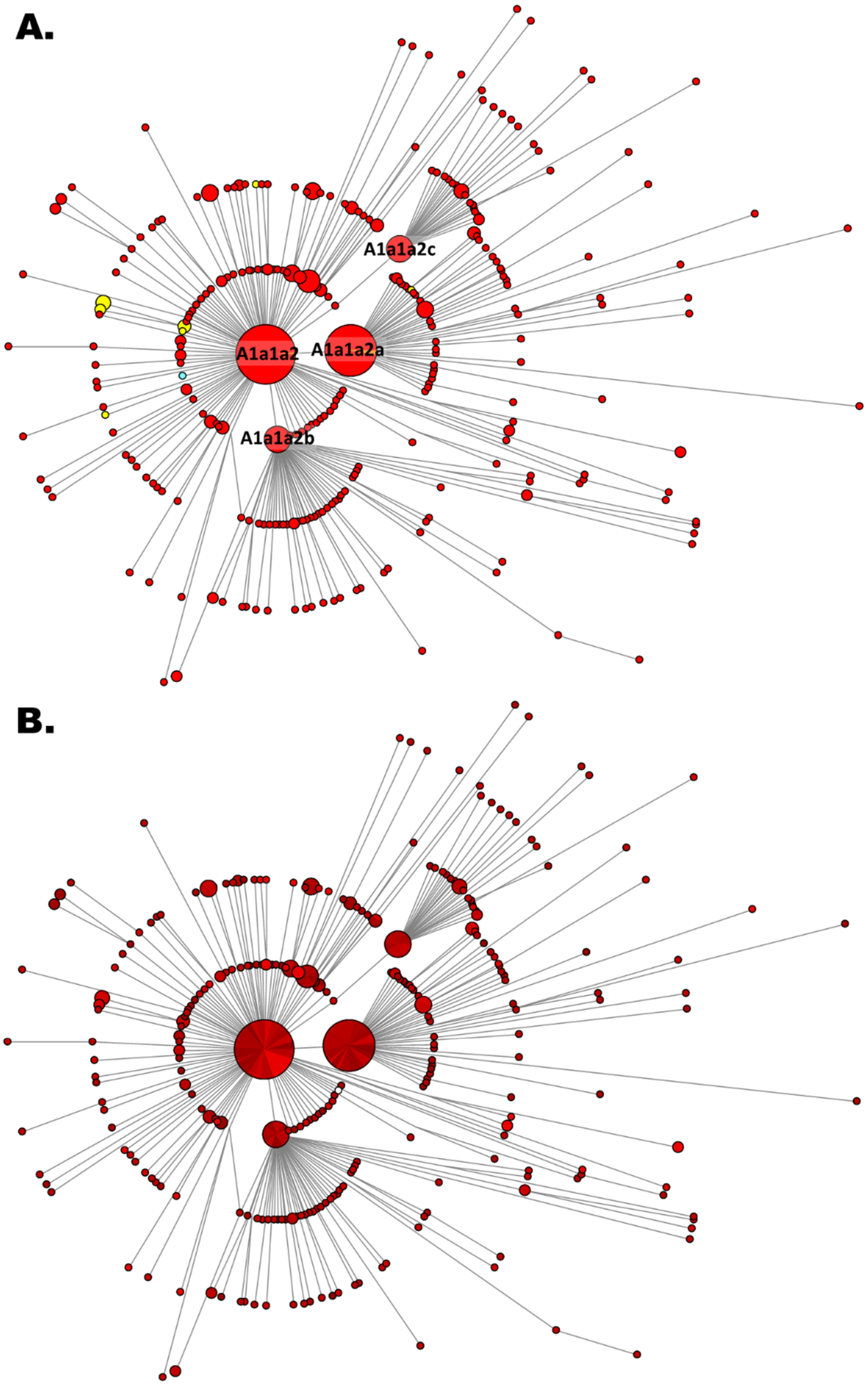

Figure S50. Reduced median network of clades of clade A1a1a2. Samples are colored according to geography (A) and time of collection (B) following the legend in Figure S4.

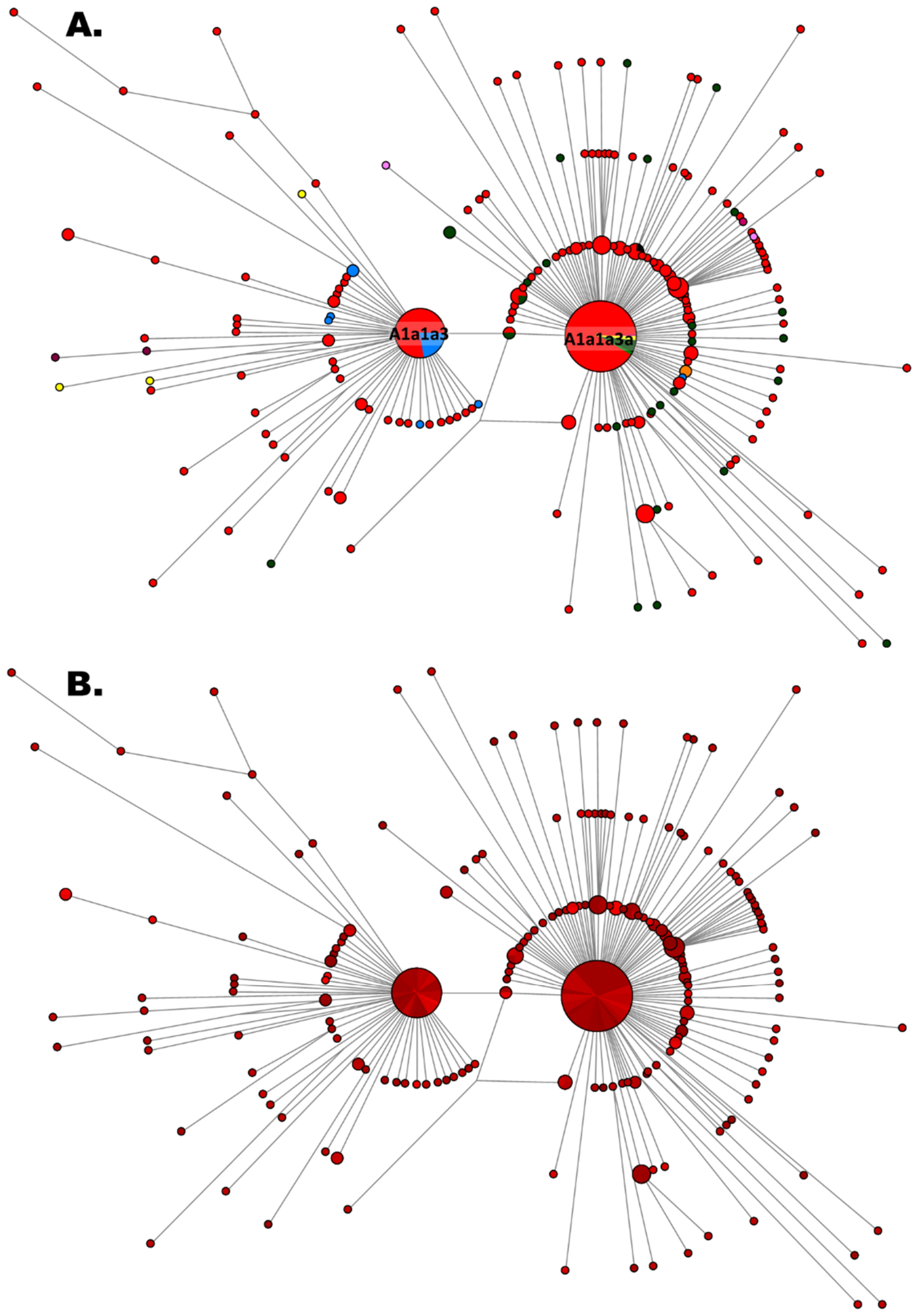

Figure S51. Reduced median network of clades of clade A1a1a3. Samples are colored according to geography (A) and time of collection (B) following the legend in Figure S4.

A.

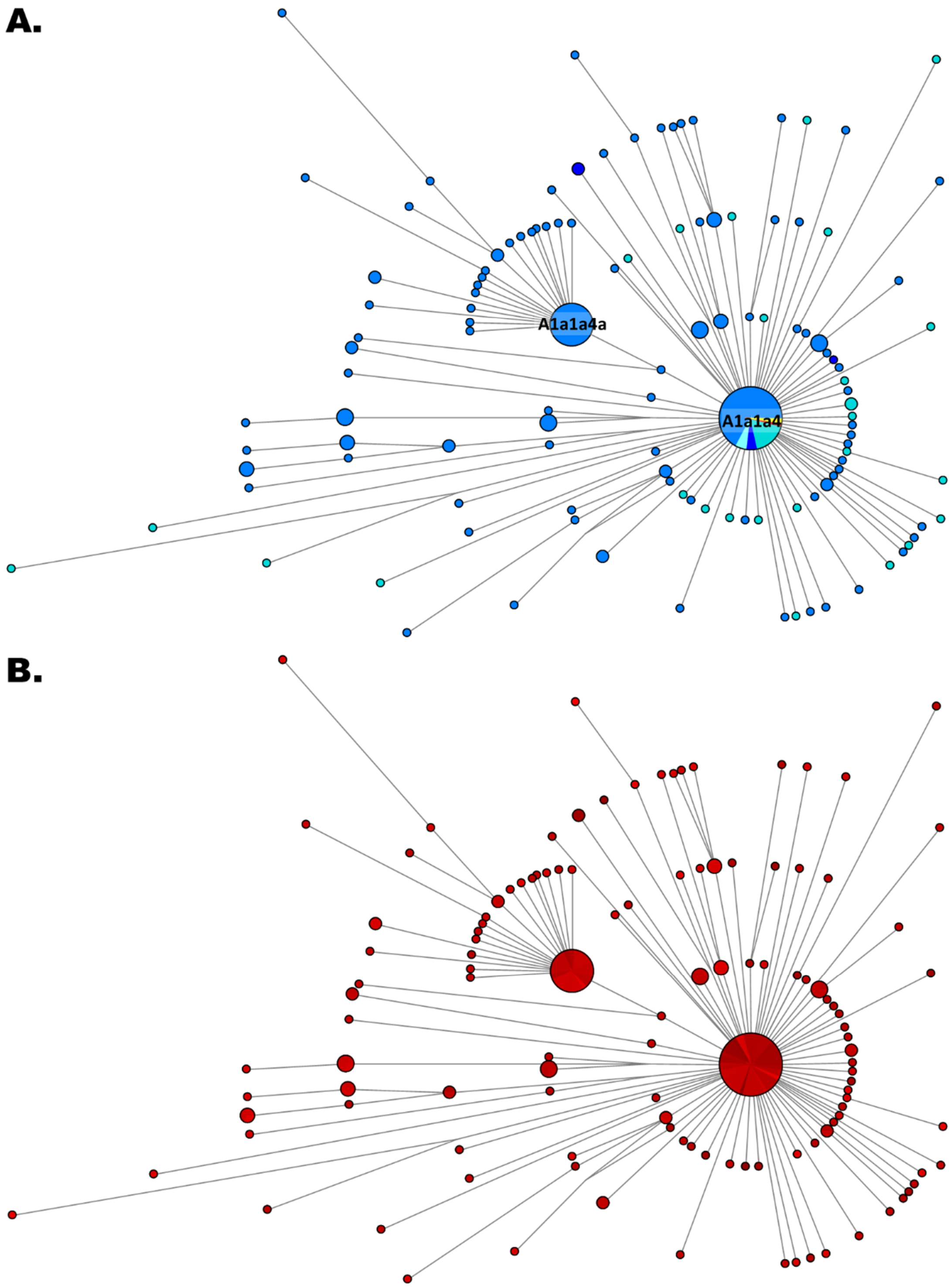

B.

Figure S52. Reduced median network of clades of clade A1a1a4. Samples are colored according to geography (A) and time of collection (B) following the legend in Figure S4.

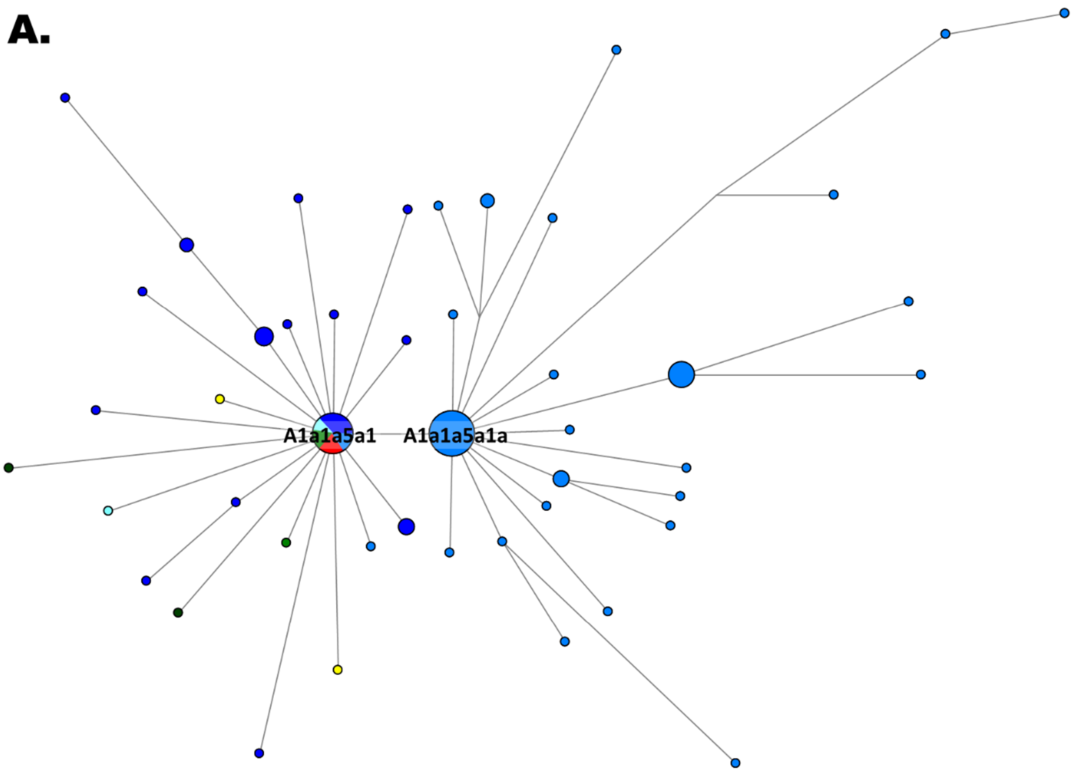

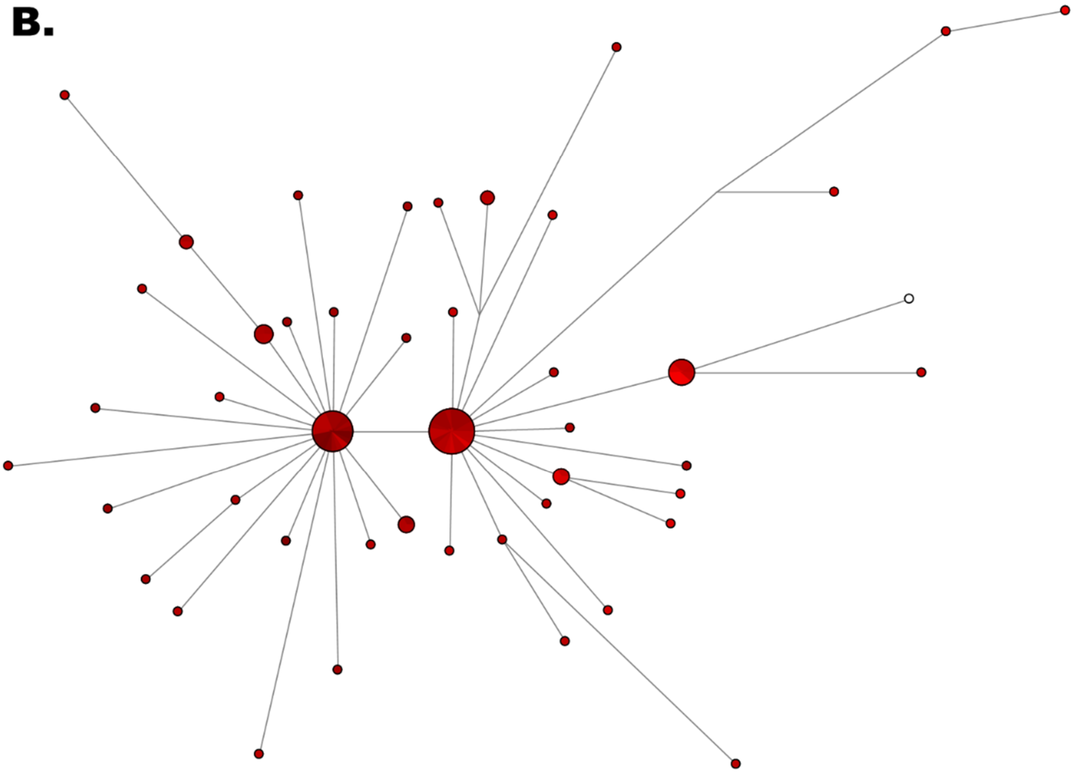

Figure S53. Reduced median network of clades of clade A1a1a5a1. Samples are colored according to geography (A) and time of collection (B) following the legend in Figure S4.

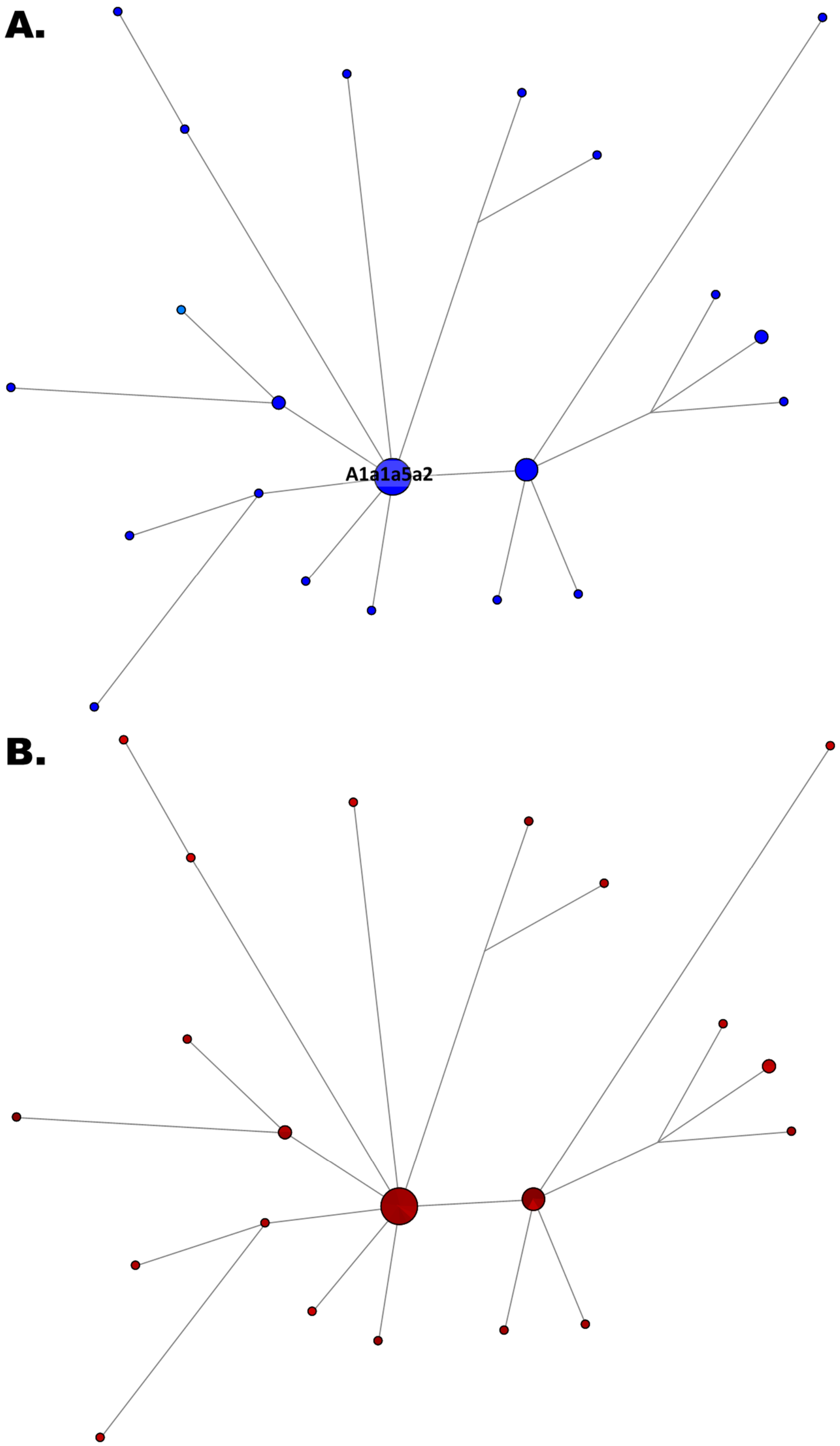

Figure S54. Reduced median network of clades of clade A1a1a5a2. Samples are colored according to geography (A) and time of collection (B) following the legend in Figure S4.

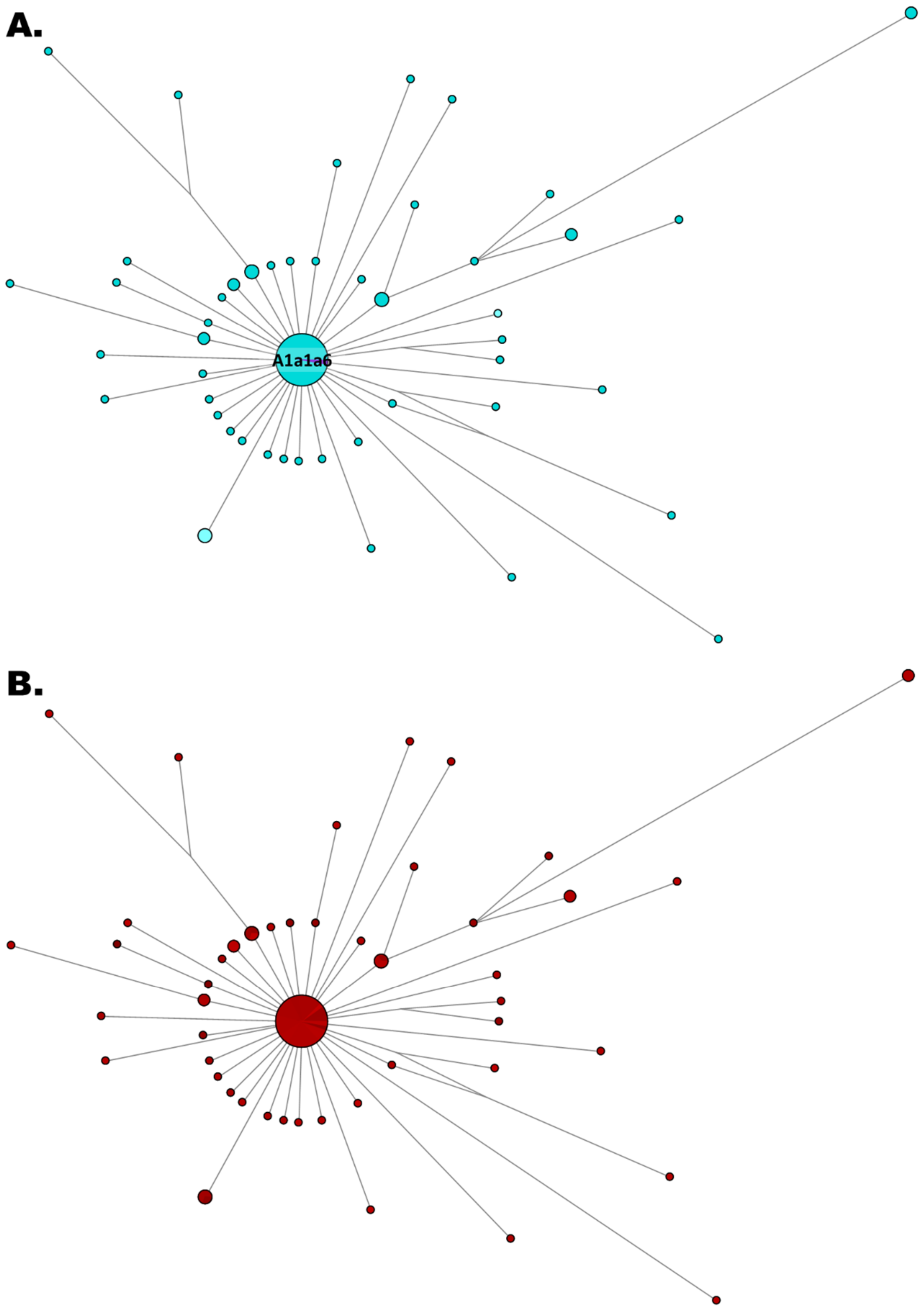

Figure S55. Reduced median network of clades of clade A1a1a6. Samples are colored according to geography (A) and time of collection (B) following the legend in Figure S4.

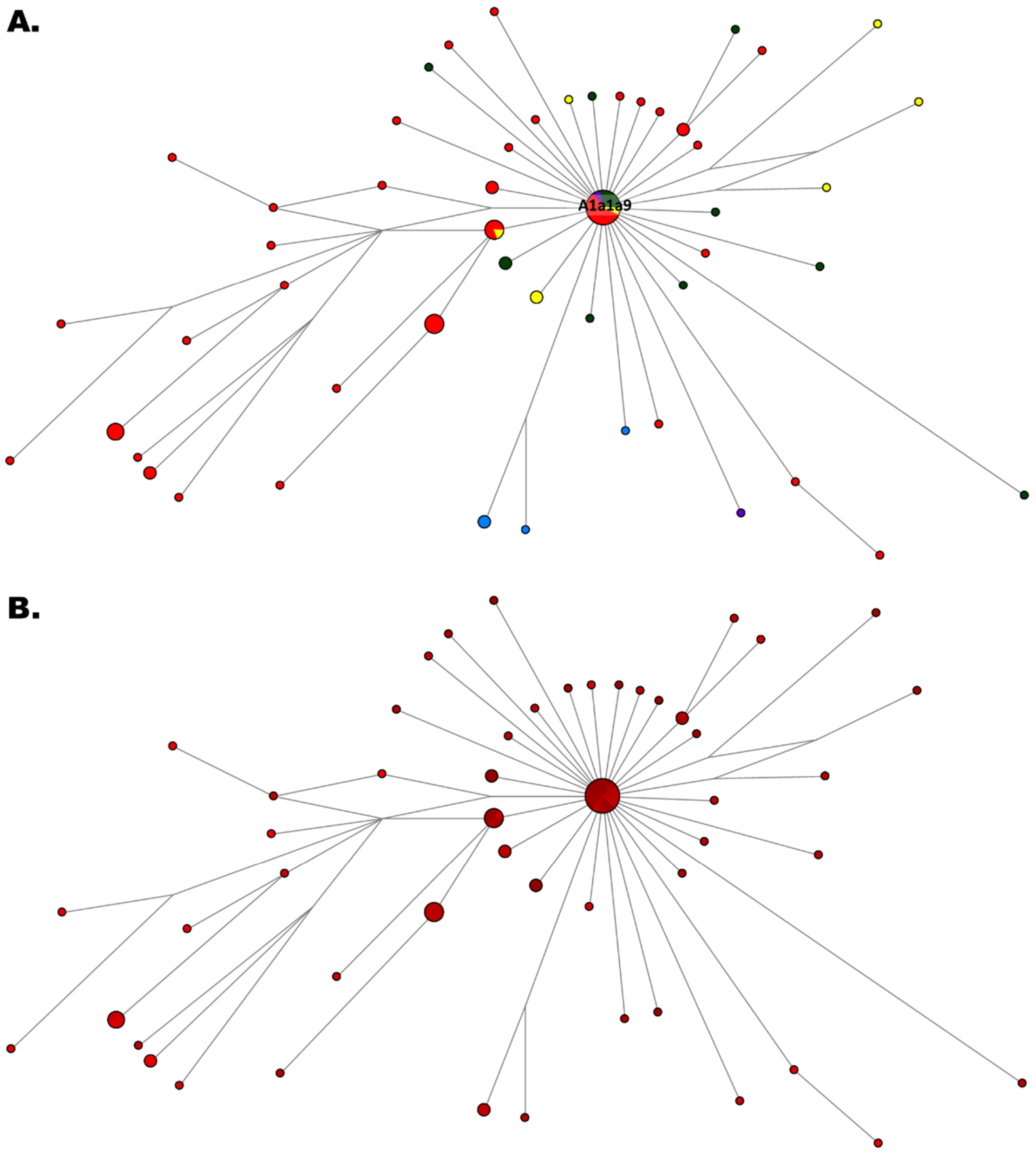

Figure S56. Reduced median network of clades of clade A1a1a9. Samples are colored according to geography (A) and time of collection (B) following the legend in Figure S4.

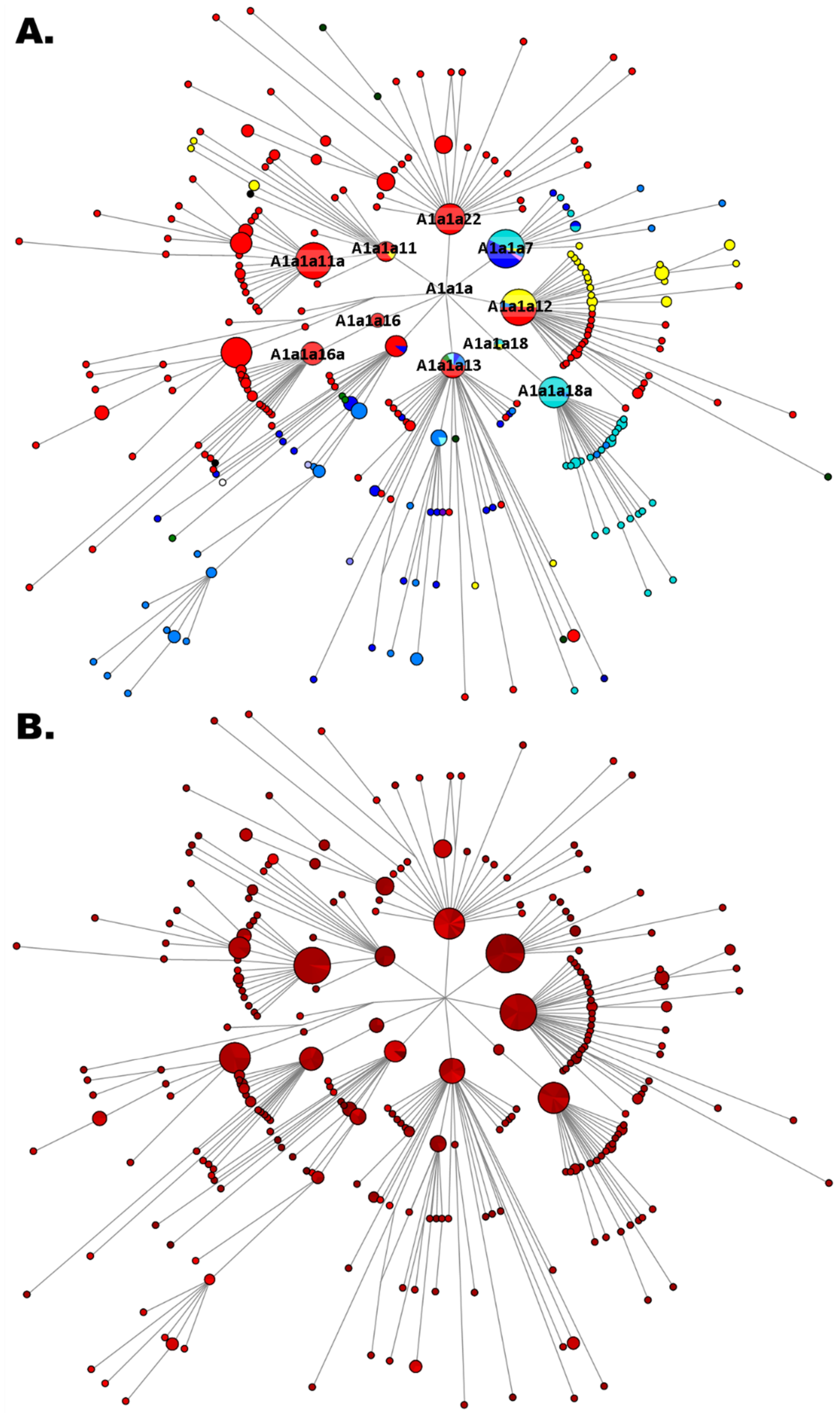

Figure S57. Reduced median network of subclades of clade A1a1a. Samples are colored according to geography (A) and time of collection (B) following the legend in Figure S4.
